# Combinative Protein Expression of Immediate Early Genes c‐Fos, Arc, and Npas4 Along Aversive and Appetitive Experience‐Related Neural Networks

**DOI:** 10.1002/hipo.70030

**Published:** 2025-08-08

**Authors:** Mary Arai, Hisayuki Osanai, Chris C. Snell, Kaylea E. Gawf, Takashi Kitamura, Sachie K. Ogawa

**Affiliations:** ^1^ Department of Psychiatry University of Texas Southwestern Medical Center Dallas Texas USA; ^2^ Peter O'Donnell Jr. Brain Institute University of Texas Southwestern Medical Center Dallas Texas USA; ^3^ Department of Neuroscience University of Texas Southwestern Medical Center Dallas Texas USA

**Keywords:** co‐expression, context conditioning, immediate early genes, immunohistochemistry, memory engram cell

## Abstract

Expression of immediate early genes (IEGs) is critical for memory formation and has been widely used to identify the neural substrate of memory traces, termed memory engram cells. Functions of IEGs have been known to be different depending on their types. However, there is limited knowledge about the extent to which different types of IEGs are selectively or concurrently involved in the formation of memory engram. To address this question, we investigated the combinative expression of c‐Fos, Arc, and Npas4 proteins using immunohistochemistry following aversive and rewarding experiences across subregions in the prefrontal cortex (PFC), basolateral amygdala (BLA), hippocampal dentate gyrus (DG), and retrosplenial cortex (RSC). Using an automated cell detection algorithm, we found that expression patterns of c‐Fos, Npas4, and Arc varied across different brain areas, with a higher increase of IEG expressing cells in the PFC and posterior BLA than in the DG. The combinative expression patterns, along with their experience‐induced changes, also differed across brain areas; the co‐expression of IEGs increased in the PFC and BLA following experience, whereas the increase was less pronounced in the DG and RSC. Furthermore, we demonstrate that different area‐to‐area functional connectivity networks were extracted by different IEGs. These findings provide insights into how different IEGs and their combinations identify engram cells, which will contribute to a deeper understanding of the functional significance of IEG‐tagged memory engram cells.

## 1 Introduction

Formation of long‐term memory requires transcription and protein synthesis (Asok et al. 2019; Barondes and Jarvik 1964; Dash et al. 1990; Davis and Squire 1984; Flexner et al. 1963; Kandel 2001; Silva et al. 1998). Immediate‐early genes (IEGs) have rapid and transient transcription upon extracellular stimulation (Greenberg and Ziff 1984; Gu et al. 2023; Sheng and Greenberg 1990). IEGs are involved in long‐term synaptic plasticity and memory, and are widely used as endogenous markers of neuronal activity, learning‐induced plasticity, and pharmacological activation (Barth et al. 2004; Fuentes‐Ramos and Barco 2024; Guzowski et al. 1999; Hoffman et al. 1993; Minatohara et al. 2015; Okuno 2011; Salery et al. 2021; Yokose et al. 2024; Yokose et al. 2023). Furthermore, IEGs have been utilized for the identification of memory engram cells, which are subpopulations of neurons that are activated by a salient experience and subsequently undergo biological changes to encode a specific memory episode (Josselyn et al. 2015; Josselyn and Tonegawa 2020; Kandel et al. 2014; Liu, Ramirez, et al. 2012; Silva et al. 2009). Transgenic approaches using IEGs have enabled the identification of memory engram cells by visualizing and optogenetically/chemogenetically manipulating IEG‐expressing cells, allowing the tracking of memory‐bearing cells and investigating their causal roles in learning and memory (Barth et al. 2004; Choi et al. 2018; Denny et al. 2014; Kitamura et al. 2017; Liu, Ramirez, et al. 2012; Marks et al. 2022; Ortega‐de San Luis and Ryan 2022; Reijmers et al. 2007; Tanaka et al. 2018; Terranova et al. 2022; Terranova et al. 2023; Tonegawa et al. 2015; Vetere et al. 2019; Wang et al. 2006; Yamamoto et al. 2021). Importantly, different types of IEG have different roles in synaptic plasticity and memory. For example, c‐Fos, a transcription factor IEG (Morgan et al. 1987; Sagar et al. 1988; Yap and Greenberg 2018), is essential for synaptic plasticity and memory consolidation (Fleischmann et al. 2003; Katche et al. 2010; Kemp et al. 2013), with increased dendritic spine density in expressing cells (Choi et al. 2018; Ryan et al. 2015; but see Uytiepo et al. 2025). Activity‐regulated cytoskeletal protein (Arc), an effector IEG (Guzowski et al. 1999; Nikolaienko et al. 2018), influences long‐term memory (Plath et al. 2006) by heterosynaptically weakening inactive synapses (El‐Boustani et al. 2018; Minatohara et al. 2015; Okuno et al. 2012; Yap and Greenberg 2018). Neuronal PAS domain protein 4 (Npas4), another transcription factor IEG (Lin et al. 2008), is crucial for memory consolidation (Ramamoorthi et al. 2011; Weng et al. 2018) and regulates excitatory‐inhibitory synaptic balance (Spiegel et al. 2014; Sun and Lin 2016). However, the difference in the type of IEG has not been relatively considered in identifying memory engram cells because there remains limited knowledge about the extent to which different IEGs are selectively or concurrently involved in engram cell formation.

Several studies have reported the co‐expression (Gonzales et al. 2020; Stone et al. 2011) and segregation (Sun et al. 2020; Ye et al. 2016) of different IEGs after learning. However, those investigations are limited to specific IEGs, behaviors, and brain regions. The aim of this study is to address the extent to which different types of IEGs are selectively or concurrently expressed in individual cells in multiple brain regions simultaneously. Using the automated cell detection method we previously proposed (Osanai et al. 2025), we investigated the combinative protein expression of c‐Fos, Arc, and Npas4 with IHC after aversive and reward experience across the mPFC, basolateral amygdala (BLA), hippocampal dentate gyrus (DG), and retrosplenial cortex (RSC), which are implicated in aversive and appetitive memory (Giustino and Maren 2015; Gore et al. 2015; Gourley and Taylor 2016; Kesner 2018; Kheirbek et al. 2013; Kirk et al. 2017; Kitamura et al. 2017; Kwapis et al. 2015; Redondo et al. 2014; Sierra‐Mercado et al. 2011; Sun et al. 2021; Terranova et al. 2022; Vedder et al. 2017).

## 2 Materials and Methods

### 2.1 Animals

All procedures relating to mouse care and experimental treatments conformed to NIH and Institutional guidelines, and were conducted with the approval of the UT Southwestern Institutional Animal Care and Use Committee (IACUC). A total of 18 male C57BL/6J mice between 8 and 16 weeks old were used. Mice were group housed with littermates (2–5 mice per cage) in a 12‐h light/dark cycle with light from 6 am to 6 pm until a day before experiments.

### 2.2 Sample Preparation and Imaging

We prepared brain section samples with three different conditions: home cage (HC), contextual fear conditioning (CFC), and reward experience (RE) groups (*n* = 6 for each group, three to six sections per animal). Mice had ad libitum access to food and water except for the RE group.

#### 2.2.1 Home Cage

Mice were separated into individual cages 1 day before the sampling, and then deeply anesthetized with a ketamine (75 mg/kg)/dexmedetomidine (1 mg/kg) (K/D) cocktail for transcardial perfusion with 4% paraformaldehyde (PFA) in PBS. Brains were removed and post‐fixed in 4% PFA in PBS at 4°C for 1–3 days.

#### 2.2.2 Contextual Fear Conditioning

Mice were separated into individual cages 1 day before CFC. Foot‐shock stimulation was provided based on the previous reports (Osanai et al. 2025; Osanai et al. 2023; Terranova et al. 2022). In this study, a fear apparatus with a 24 cm W × 20 cm D × 20 cm H chamber (Med Associates) was used, and a mouse was placed in the fear stimulation chamber for a 3‐min habituation period and for a subsequent 3‐min shock period. During the shock period, the mouse received three foot shocks (0.75 mA, 2‐s) with 58‐s inter‐shock intervals. After the stimulation, the mouse was returned to its home cage for 1 h. The mouse was then immediately anesthetized deeply with a K/D cocktail and transcardially perfused with 4% PFA in PBS. Brains were removed and post‐fixed in 4% PFA in PBS at 4°C for 1–3 days. The shock chamber was cleaned before starting each experiment. Freezing behavior during the fear conditioning experiments was recorded in the videos and analyzed by AnyMaze software (Stoelting, Wood Dale, IL). The freezing ratio in each period (habituation‐ or shock‐period) was calculated as the total freezing time during each period divided by the total time of each period (3 min).

#### 2.2.3 Reward Experience

Mice were separated into individual cages and subjected to 1 week of food restriction with access to a small amount of food daily, resulting in a reduction of their body weight to ~85% of the initial weight. The RE protocol was conducted in the same chamber as the CFC. After the food restriction period, each mouse was allowed to explore the chamber with food pellets for 30 min. Eating behavior during chamber exploration was confirmed through video recording and by measuring the weight reduction of the provided food pellets. The mouse was then returned to its home cage for 1 h. Following this, the mouse was immediately anesthetized deeply with a K/D cocktail and transcardially perfused with 4% PFA. Brains were removed and post‐fixed in 4% PFA in PBS at 4°C for 1–3 days. The chamber was cleaned before starting each experiment. Eating behavior was manually identified using recorded videos. The ratio of eating duration was calculated by dividing the total duration of eating behavior by the total duration of the experiment (30 min).

#### 2.2.4 Immunohistochemistry and Imaging

The fixed brains were sectioned using a vibratome (Leica VT100S) with a thickness of 60 μm. For immunohistochemistry (IHC), tissue sections were washed with PBS, blocked with 0.03% Triton‐X PBS (PBS‐T) with 5% normal donkey serum (NDS) (Jackson ImmunoResearch Labs; RRID: AB_2337258) for 30 min, and then incubated with primary antibodies diluted in the PBS‐T with 5% NDS for two nights at 4°C. After washing with PBS (3 × 5 min), tissue sections were subsequently incubated with secondary antibodies in the PBS‐T with 5% NDS for 2 h at room temperature. Primary antibodies were chicken anti‐NeuN (1/1000, Millipore Sigma, ABN91; RRID: AB_11205760), guinea pig anti‐Arc (1/500, synaptic systems, 156005; RRID: AB_2151848), rabbit anti‐Npas4 (1/1000, Activity signaling, AS‐AB18A‐300), and goat anti‐c‐Fos (1/1000, Santacruz, sc‐52‐G; RRID: AB_2629503). Secondary antibodies were donkey anti‐chicken DyLight 405 (1/500, Jackson ImmunoResearch Labs; RRID: AB_2340373), donkey anti‐guinea pig AlexaFluor488 (1/500, Jackson ImmunoResearch Labs; RRID: AB_2340472), donkey anti‐rabbit AlexaFluor546 (1/500, ThermoFisher Scientific; RRID: AB_2534016), and donkey anti‐goat AlexaFluor633 (1/500, Jackson ImmunoResearch Labs; RRID: AB_2535739). After incubating in the secondary antibody solution, the tissue sections were washed in PBS (2 × 5 min) and mounted in VECTASHIELD antifade mounting medium (Vector Laboratories) on glass slides.

All fluorescence images (0.624 μm/pixel) were acquired under the same imaging conditions using Zeiss LSM800, 10× objective lens (NA: 0.45), and Zen Blue software (Zeiss). Details of the imaging conditions were described previously (Osanai et al. 2025).

### 2.3 Analysis

IEG‐ and NeuN‐positive cells were detected in the prelimbic (PL) and infralimbic (IL) regions of PFC, anterior and posterior BLA (aBLA and pBLA), granule cell layers of dorsal and ventral DG (dDG and vDG), and dorsal/ventral parts of anterior and posterior RSC (dorsal aRSC, ventral aRSC, dorsal pRSC, and ventral pRSC), whose boundaries were determined based on the Allen Brain Reference Atlas (Allen Institute for Brain Science 2004). From bregma, PL and IL were determined in the coronal sections at +1.845 to +1.42 mm, aBLA was at −1.255 to −1.655 mm, pBLA was at −2.355 to −2.78 mm, dDG was at −1.655 to −2.255 mm, vDG was at −3.28 to −3.455 mm, aRSC was at −1.255 to −2.255 mm, and pRSC was at −2.78 to −3.78 mm. The regions of interest (ROI) for each brain region were manually drawn using ImageJ (Schindelin et al. 2012). The images of each channel (NeuN, c‐Fos, Npas4, Arc) and the ROI information were imported into MATLAB R2024b (Mathworks) for further analysis. For the analysis in the PL and dDG, we included the data used in the previous report (Osanai et al. 2025).

#### 2.3.1 Automated Cell Detection

In this study, we used the newly developed method, automated cell detection after background assumption (ADABA) algorithm, written in MATLAB that we proposed previously (Osanai et al. 2025). Briefly, the algorithm subtracted the background of the image and then the background‐cleaned image was used for cell detection. First, the images were converted into 8‐bit gray scale, and median filters were applied (c‐Fos, Npas4, and Arc images: 11 × 11 pixels; NeuN image: 7 × 7 pixels). For assuming background pattern, we first determined the intense signal pixels in the image by drawing 20 intensity contours using Otsu's method (Otsu 1979) which is implemented in MATLAB Image Processing Toolbox (*multithresh.m*). A contour which covers more than 80% (for Arc and NeuN images) or 95% (for c‐Fos and Npas4 images) of the image was selected for further calculation. The inside contour areas were filled with the neighboring intensity, and the background pattern was assumed by filtering with a spatial moving average filter (31 × 31 pixels). The assumed background was then subtracted from the median‐filtered image. Then, thresholding was conducted on the background‐subtracted image with *T* = *k* × *σ*, where *T* is the threshold, *σ* is the standard deviation intensity of the background‐subtracted image, and *k* is the coefficient parameter; *k* = 5 for c‐Fos, Npas4, and Arc, and *k* = 2 for NeuN positive cell detections. The thresholded signals were denoised and smoothed by morphological operations of erosion‐reconstruction with five pixels distance and closing with two pixels distance. Signals whose areas are smaller than 50 pixels were regarded as noise and removed for further processing. The smoothed thresholded signals were then segmented with a watershed algorithm to detect c‐Fos, Npas4, Arc, and NeuN positive cells in the image. IEGs were assessed as co‐localized within a cell if their detected areas share more than five pixels. Cell densities were obtained by calculating the number of positive cells per areas of the ROI. Expression levels of IEGs were measured by peak fluorescent intensity of each detected cell. The ratio of IEG single‐, double‐, and triple‐positive cells was calculated by dividing the number of single‐, double‐, and triple‐positive cells by the total number of c‐Fos, Npas4, or Arc positive cells in Figures 4, 5, 6, 7, or by the total number of IEG‐positive cells in Figure S14.

Similar to the previous report (Osanai et al. 2025), to evaluate the accuracy of the automated cell detection, manual cell detection was performed by an experimenter that was blinded to the result of the automated cell detection using ImageJ (Schindelin et al. 2012). For manual detection of IEG‐positive cells, random 77 images of HC, 129 images of CFC, and 128 images of RE of the PL, IL, aBLA, pBLA, dDG, and vDG (total 334 images) from six animals per each experimental group were used. Within these images, true positive and false positive were identified manually after the automated detection using random 7, 9, 7, and 9 images for NeuN, c‐Fos, Npas4, and Arc, respectively. Also, random 5 images were used for manual detection of NeuN‐positive cells. The Precision, or false positive detection rate, was calculated as Precision = TP/(TP + FP), where true positive (TP) and false positive (FP) were manually checked after the automated detection. To evaluate the ratio that cells detected manually were also detected in the automated algorithm, the Auto‐Manual match rate, or Sensitivity, was calculated as Match/(Match + Eye_Only), where Match indicates the number of cells detected both by the automated and manual approach and Eye_Only indicates the number of cells identified only by manually. To evaluate the ratio of cells overlooked in manual identification but detected in the automated algorithm, the Sensitivity‐increase rate was calculated as Auto_Only/(Match + Auto_Only) where Auto_Only indicates the number of cells that were not identified manually but detected in the automated algorithm. High sensitivity indicates that the automated method does not overlook manually detected cells, and high sensitivity‐increase rate indicates that the automated method detects more cells overlooked by manual detection. The custom code used in this study is available at https://github.com/HisayukiOsanai/CellDetection.

#### 2.3.2 Network Analysis

To calculate IEG‐based functional connectivity networks, correlation matrices were generated based on interregional correlations in the number of IEG‐positive cells (Silva et al. 2019; Takeuchi et al. 2022; Tanimizu et al. 2017; Vetere et al. 2017; Wheeler et al. 2013). Correlation matrices of Pearson r values were computed between all 10 brain regions with a vector of size 6 (i.e., 6 animals) within each experimental group (HC, CFC, RE) and each IEG group (c‐Fos, Npas4, Arc, and combinations of them). Hierarchical clustering of the correlation matrix was visualized by average‐linkage hierarchical clustering with dissimilarity index, or distance, calculated by distance = 1 − |*r*| (Liu, Zhu, et al. 2012; Takeuchi et al. 2022).

Functional connectivity networks were constructed by thresholding the correlation matrices and visualized using MATLAB. The graphs represent brain areas (nodes) and network connection lines (edges) which represent Pearson correlations |*r*| between brain areas. A correlation of |*r*| > 0.7 was considered strong and used for thresholding the network connections. Line thickness and node sizes are proportional to the |*r*| value between brain areas and to the number of connections each brain area has, individually. Complexity of networks was evaluated by connectivity per brain area which indicates the number of connections (edges) per brain area (node), and by connectivity per effective node which indicates the number of connections (edges) per brain area that has at least one connection (effective node). To evaluate dissimilarity between graphs, graph‐edit‐distance (GED) (Bai et al. 2019; Tantardini et al. 2019; Wills and Meyer 2020) and Sum of Differences in Edge‐Weight Values (SDEWV) (Wang et al. 2019) were calculated. GED is a measurement of the minimum number of operations required to transform one graph into another, which we calculated by the sum of all elements of the difference between two graph adjacency matrices: $\mathrm{GED}=\sum_{i,j}D_{i,j}$, where $D_{i,j}$ is the element of difference matrix **D** = **A** − **A′**, with **A** and **A′** being the adjacency matrices of the two graph **G** and **G′**. SDEWV is the sum of absolute differences in edge weights between two graphs, which we calculated as $\text{SDEWV}=\sum_{i,j}|e_{i,j}-e_{i,j}^{\prime }|$, where *e* and *e*′ are edge weight between nodes *i* and *j* in the two graphs.

#### 2.3.3 Statistics

**FIGURE 1:**
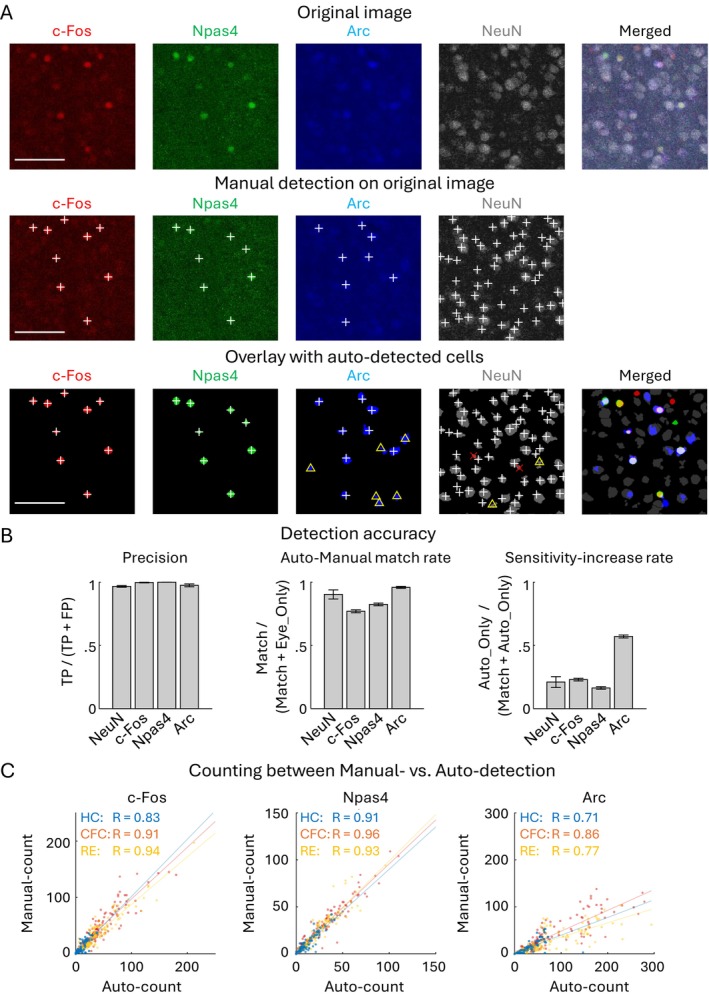
Automated detection of IEG positive cells and the detection accuracy. (A) Top: Original images of c‐Fos, Npas4, Arc, and NeuN expressed cells in aBLA. Middle: Overlay of the manually detected positive cells (white “+” marks) on the original images. Bottom: Auto‐detected cells (colored cells), overlay of the manually detected positive cells and manually verified false‐positive detections (red “×” marks). Yellow “△” marks indicate cells overlooked in the manual detection but detected in the automated algorithm. Scale bars, 100 μm. (B) Evaluation of auto‐detection accuracy with Precision, Auto‐Manual match rate, and Sensitivity increase rate. Precision indicates false positive ratio in the automated cell detection per total detected cells, that is Precision = TP/(TP + FP), where true positive (TP) and false positive (FP) were identified manually after the automated detection (*n* = 7, 9, 7, and 9 sections for NeuN, c‐Fos, Npas4, and Arc). Auto‐Manual match rate indicates the ratio of cells which were both manually and automatically detected, such that Auto‐Manual match rate = Match/(Match + Eye_Only), where Match indicates the number of cells which were both manually and automatically detected, and Eye_Only indicates the number of cells identified only by manual detection (*n* = 5 sections for NeuN, *n* = 334 sections for c‐Fos, Npas4, and Arc). Sensitivity increase rate indicates ratio that cells were not detected manually but detected automatically, that Sensitivity‐increase rate = Auto_Only/(Match + Auto_Only), where Auto_Only indicates the number of cells which were not identified manually but detected automatically (*n* = 5 sections for NeuN, *n* = 334 sections for c‐Fos, Npas4, and Arc). (C) Correlation of automatically and manually detected cell number, with *R* indicating Pearson correlation coefficient (*p* < 0.001 for all). HC: *N* = 77, CFC: *N* = 129, RE: *N* = 128 sections. The sections used for accuracy analysis were randomly selected from the PFC, BLA, and DG. Total counted cells were, for manual‐ vs. auto‐detection, for c‐Fos positive cells, HC: 786 vs. 751 cells, CFC: 5914 vs. 5653 cells, and RE: 4604 vs. 4922 cells; for Npas4 positive cells, HC: 800 vs. 875 cells, CFC: 3548 vs. 3476 cells, and RE: 2713 vs. 2696 cells; for Arc positive cells, HC: 893 vs. 1965 cells, CFC: 4983 vs. 10,101 cells, and RE: 3415 vs. 9136 cells.

Statistical analyses were performed using MATLAB with *n* = 6 animals for each experimental group except detection accuracy analysis in Figure 1 and intensity analyses in Figures 3B, S8, S9, and S18–S20. All bar plots and error bars represent mean ± standard error, and box plots in the violin plots display minimum, 25th percentile, median, 75th percentile, and maximum values. One‐way ANOVA followed by post hoc Tukey test was used to analyze differences between groups. For cell‐intensity analysis, we used the nonparametric Kruskal–Wallis test followed by the Dunn–Šidák test. Effect sizes were calculated using Cohen's d, where *d* = 0.2, 0.5, 0.8, and < 0.2 are considered as small, medium, large, and negligible effects (Cohen 1988; Thomas et al. 1991). For effect sizes in the cell‐intensity analysis, we calculated Cliff's *δ*, where *δ* = 0.147, 0.33, 0.474, and < 0.147 correspond to small, medium, large, and negligible effects (Cliff 1993; Macbeth et al. 2011; Meissel and Yao 2024; Romano et al. 2006). *p* < 0.05 was considered to be statistically significant. *, **, and *** indicate *p* < 0.05, 0.01, and 0.001, respectively. Due to the large sample size in the cell‐intensity analysis, we assessed a non‐negligible difference using effect size rather than relying solely on *p* values in the intensity analysis (Nakagawa and Cuthill 2007). #, ##, and ### indicate *δ* ≥ 0.147, 0.33, and 0.474, respectively, with all *p* < 0.05.

## 3 Results

### 3.1 Automated Detection of c‐Fos, Npas4, and Arc Expressing Cells

For the comprehensive investigation of IEG‐expressing cells in multiple brain regions, we used the automated cell detection method. Brain slice images were captured using the confocal microscope following the IHC staining with c‐Fos‐, Npas4‐, and Arc‐antibodies (Figure 1A). Consistent with our previous results (Osanai et al. 2025), the automated cell detection has high accuracy compared with manual detection; the false positive detection was lower than 5% (Precision > 0.96), and the automated detection identified more than 77% of the manually identified cells with increased sensitivity in the confocal images of the BLA, PFC, and DG (Figure 1B). Some IEG‐stained images contained both strongly and weakly labeled cells, making consistent manual cell counting difficult; cells with similar intensity were more likely to be counted manually in images with sparse cell distribution but were often overlooked in images with dense and variable‐intensity staining cells (Figure 1A yellow triangles) (Osanai et al. 2025). Such observation bias can be avoided by automated detection, resulting in higher sensitivity of Arc positive cell detection (Figure 1B, right). The number of detected cells was highly correlated with *R* > 0.7 between automated and manual cell detections for all c‐Fos, Npas4, and Arc positive cells (Figures 1C and S1). The results indicate that our automated cell detection algorithm has high precision and helps reduce observation bias in the manual detection of IEG‐expressing cells.

### 3.2 c‐Fos, Npas4, and Arc Expression in Various Brain Areas

**FIGURE 2:**
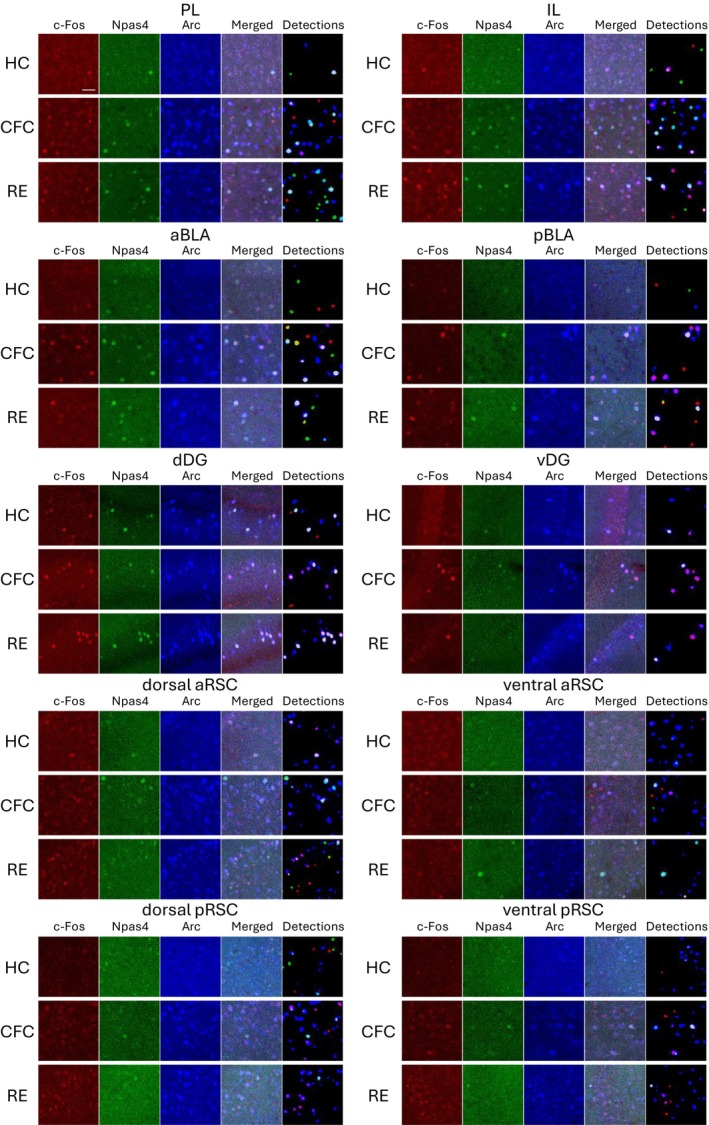
Expression of c‐Fos, Npas4, and Arc in multiple brain regions. Images of c‐Fos (red), Npas4 (green), Arc (blue), merged, and auto‐detected cells in the prelimbic (PL), infralimbic (IL), anterior basolateral amygdala (aBLA), posterior BLA (pBLA), dorsal dentate gyrus (dDG), and ventral DG (vDG), dorsal anterior RSC (aRSC), ventral aRSC, dorsal posterior RSC (pRSC), and ventral pRSC. Top: Home cage (HC); Middle: Contextual fear conditioning (CFC); Bottom: Reward experience (RE) groups. Scale bars, 40 μm. Larger field‐of‐view images are shown in Figures S2–S7.

**FIGURE 3:**
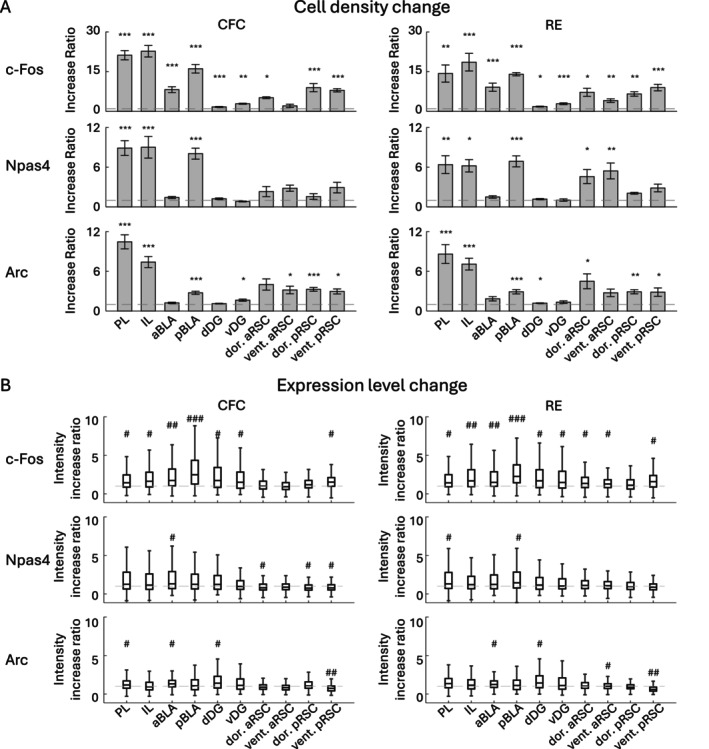
Changes of cell densities and expression levels of c‐Fos, Npas4, and Arc positive cells. (A) Fold changes of c‐Fos, Npas4, and Arc positive cell densities by CFC and RE in each brain region, compared with HC group. (B) Fold changes of c‐Fos, Npas4, and Arc expression levels of cells by CFC and RE in each brain region, compared with HC group. Statistical tests were conducted between groups, as shown in Figures S8 and S9.

Due to the reliability of automated cell detection, we applied this method to identify the positive cells for all following analyses. The cell detections for c‐Fos, Npas4, and Arc were performed in the prelimbic cortex (PL) (Figures 2 and S2A), infralimbic cortex (IL) (Figures 2 and S2B), anterior BLA (aBLA) (Figures 2 and S3A), posterior BLA (pBLA) (Figures 2 and S3B), dorsal DG (dDG) (Figures 2 and S4), ventral DG (vDG) (Figures 2 and S5), dorsal/ventral anterior RSC (aRSC) (Figures 2 and S6), and dorsal/ventral posterior RSC (pRSC) (Figures 2 and S7), individually 60 min following CFC or reward experience (RE) (see Section 2). After CFC, c‐Fos^+^ cell density was significantly increased in all brain areas except the ventral aRSC compared with the home‐cage (HC) group. On the other hand, Npas4^+^ cell density was increased in the PL, IL, and pBLA. The Arc^+^ cell density was increased in the PL, IL, pBLA, vDG, ventral aRSC, and dorsal/ventral pRSC (Figures 3A, S8, S9, and S10A). After RE, the c‐Fos^+^ cell densities were increased significantly in all brain areas; Npas4^+^ cell densities were increased in the PL, IL, pBLA, and dorsal/ventral aRSC; Arc^+^ cell densities were increased in the PL, IL, pBLA, dDG, dorsal aRSC, and dorsal/ventral pRSC (Figures 3A, S8, S9, and S10A). Furthermore, after CFC, the average expression level of c‐Fos was increased in the PL, IL, a/pBLA, d/vDG, and ventral pRSC; Npas4 expression level was increased in the aBLA and decreased in the dorsal aRSC and dorsal/ventral pRSC; Arc expression level increased in the PL, aBLA, and dDG and decreased in the ventral pRSC. After RE, the average c‐Fos expression level was increased in all brain areas but the ventral aRSC; Npas4 expression level was increased in the PL and pBLA; Arc expression level increased in the aBLA, dDG, ventral aRSC, and ventral pRSC (Figures 3B, S8, S9, and S10B). The decrease of average expression level after stimulation in RSC may indicate that the number of cells with weak IEG expression were increased whereas the maximum expression level is strictly controlled compared with other brain areas once IEG is expressed. In addition, the number of IEG positive cells was correlated with animal behaviors in several brain regions (Figures S11 and S12). In CFC, the freezing duration was significantly correlated to IEG‐positive cell densities; with c‐Fos^+^ cell density in the dDG and dorsal aRSC; with Npas4^+^ cell density in the PL, pBLA, and dDG; with Arc^+^ cell density in the PL (Figure S11E). In RE, the eating duration was significantly correlated with Arc^+^ cell density in the PL and IL (Figure S12E). Thus, the expressions of c‐Fos, Npas4, and Arc are differently increased after the conditioning experience depending on the brain areas (Figure 3A). However, overall, brain areas with a greater increase in the expression of one IEG type tended to show higher expression of other types of IEGs with a correlation coefficient larger than 0.7 (Figure S13).

### 3.3 Combinative Expression of c‐Fos, Npas4, and Arc

**FIGURE 4:**
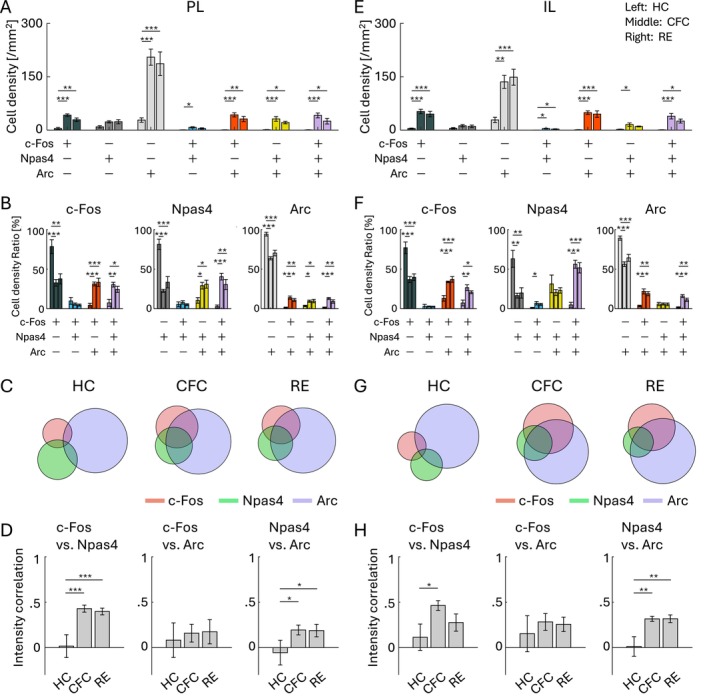
Co‐expression of IEGs in PL and IL. (A, E) Cell densities of each cell group with selective or combinative IEG expression in the PL (A) and IL (E). (B, F) Ratio of cell densities per all c‐Fos cells (left), Npas4 cells (middle), and Arc cells (right), in the PL (B) and IL (F). (C, G) Venn diagrams of c‐Fos, Npas4, and Arc positive cells in HC, CFC, and RE groups, in the PL (C) and IL (G). The size of the circles corresponds to cell densities, normalized by Arc cell density. (D, H) Average correlation of IEG expression in single cells, between c‐Fos versus Npas4 (left), c‐Fos versus Arc (middle), and Npas4 versus Arc (right), in the PL (D) and IL (H). For each group of bars, the left bar indicates HC, the middle indicates CFC, and the right indicates RE.

Next, we investigated the extent to which the different types of IEGs are selectively or co‐expressed in individual neurons. In both PL and IL, cell densities of the double‐positive cells of different IEGs and c‐Fos/Npas4/Arc triple‐positive cells tended to be increased by mouse experience (Figure 4A,E). The ratio of IEG single‐positive cells was decreased in both PL and IL by CFC or RE, but instead the ratio of the c‐Fos/Npas4/Arc triple‐positive cells and double‐positive cells of c‐Fos/Arc and Npas4/Arc were increased (Figures 4B,C,F,G and S14A,B). The expression levels of c‐Fos, Npas4, and Arc in single cells in both PL and IL were not correlated in the HC group, but the correlation between c‐Fos and Npas4 and between Npas4 and Arc increased after CFC and RE (Figure 4D,H). These indicate that c‐Fos, Npas4, and Arc tend to be independently expressed in the HC condition but become co‐expressed after CFC and RE in the PL and IL.

**FIGURE 5:**
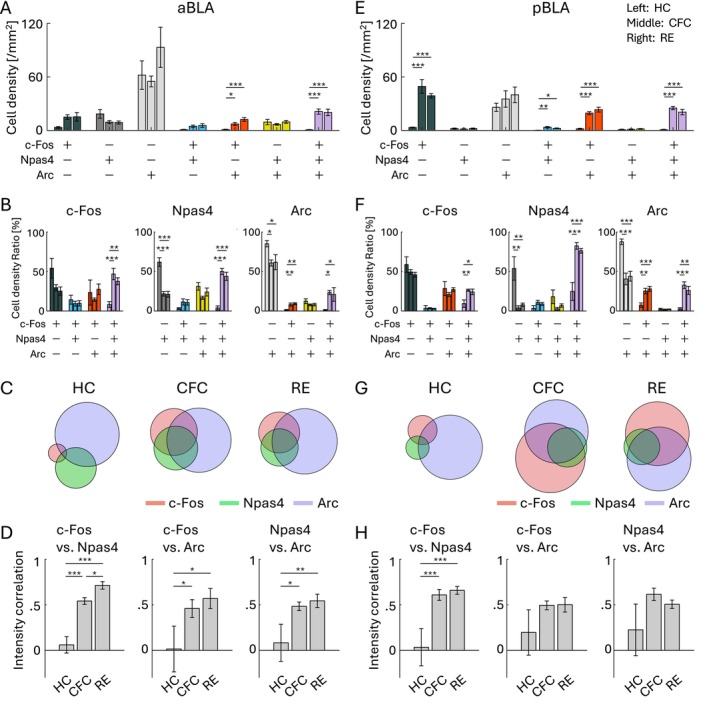
Co‐expression of IEGs in anterior and posterior BLA. (A, E) Cell densities of each cell group with selective or combinative IEG expression in aBLA (A) and pBLA (E). (B, F) Ratio of cell densities per all c‐Fos cells (left), Npas4 cells (middle), and Arc cells (right), in the aBLA (B) and pBLA (F). (C, G) Venn diagrams of c‐Fos, Npas4, and Arc positive cells in HC, CFC, and RE groups, in the aBLA (C) and pBLA (G). The size of the circles corresponds to cell densities, normalized by Arc cell density. (D, H) Average correlation of IEG expression in single cells, between c‐Fos versus Npas4 (left), c‐Fos versus Arc (middle), and Npas4 versus Arc (right), in the aBLA (D) and pBLA (H). For each group of bars, the left bar indicates HC, the middle indicates CFC, and the right indicates RE.

In the aBLA, cell densities of the c‐Fos/Npas4/Arc triple‐positive cells and c‐Fos/Arc double‐positive cells were increased by CFC and RE (Figure 5A). Also, the ratio of the c‐Fos/Npas4/Arc triple‐positive cells was increased by CFC and RE (Figures 5B,C and S14C). The expression levels of IEGs were correlated after CFC and RE but not in HC (Figure 5D). Similarly, in the pBLA, the cell density of the c‐Fos/Npas4/Arc triple‐positive cells was increased by CFC and RE, as well as c‐Fos/Npas4 and c‐Fos/Arc double‐positive cells and c‐Fos single‐positive cells (Figure 5E). The ratio of the c‐Fos/Npas4/Arc triple‐positive cells was increased by CFC and RE (Figures 5F,G and S14D). The correlation of the expression level between c‐Fos and Npas4 was significantly increased after CFC and RE compared with HC (Figure 5H). These results indicate that, similar to the results in the PFC, c‐Fos, Npas4, and Arc tend to be co‐localized after CFC and RE in the BLA.

**FIGURE 6:**
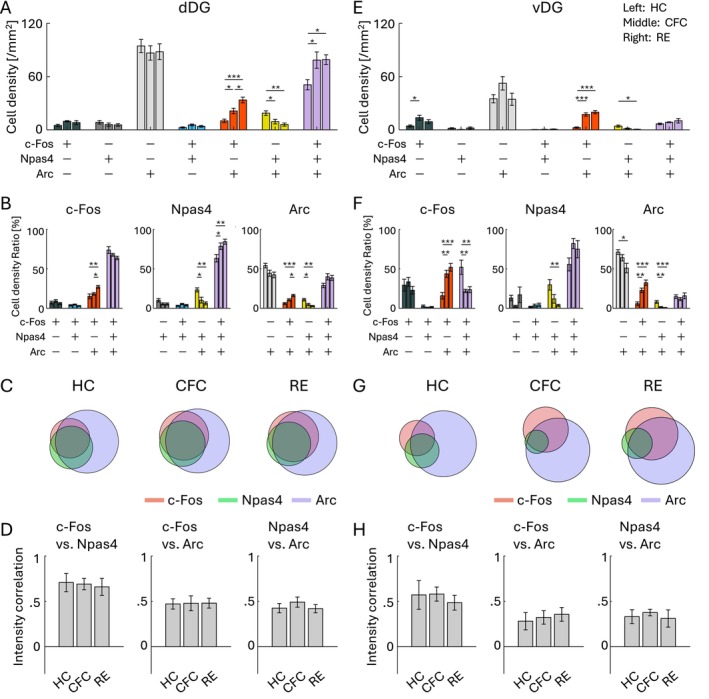
Co‐expression of IEGs in dorsal and ventral DG. (A, E) Cell densities of each cell group with selective or combinative IEG expression in the dDG (A) and vDG (E). (B, F) Ratio of cell densities per all c‐Fos cells (left), Npas4 cells (middle), and Arc cells (right), in the dDG (B) and vDG (F). (C, G) Venn diagrams of c‐Fos, Npas4, and Arc positive cells in HC, CFC, and RE groups, in the dDG (C) and vDG (G). The size of the circles corresponds to cell densities, normalized by Arc cell density. (D, H) Average correlation of IEG expression in single cells, between c‐Fos versus Npas4 (left), c‐Fos versus Arc (middle), and Npas4 versus Arc (right), in the dDG (D) and vDG (H). For each group of bars, the left bar indicates HC, the middle indicates CFC, and the right indicates RE.

On the other hand, in the DG, the cell density of the c‐Fos/Npas4/Arc triple‐positive cells was increased by CFC and RE in the dDG, but the change was not observed in the vDG (Figure 6A,E). Unlike the PFC and BLA, where the proportion of the triple‐positive cells was < 3% of the whole population in the HC group (Figure S14A–D), both dDG and vDG had a higher ratio of triple‐positive cells in HC (26.7% ± 3.0% in dDG and 12.3% ± 1.7% in vDG; Figure S14E,F) as well as in the CFC and RE groups (Figures 6B,C,F,G and S14E,F). The increase of the triple‐positive cell ratio after CFC and RE was only observed within Npas4^+^ cells but not within c‐Fos^+^ and Arc^+^ cells in the dDG (Figure 6B), and the increase was not observed in the vDG (Figure 6F,G). Unlike the PFC and BLA, CFC and RE groups did not show increased correlations of expression level in any pair between c‐Fos, Npas4, and Arc in the dDG and vDG compared with HC (Figure 6D,H). Thus, the combinative expression patterns of c‐Fos, Npas4, and Arc in the dDG and vDG were different from those in the PFC and BLA.

**FIGURE 7:**
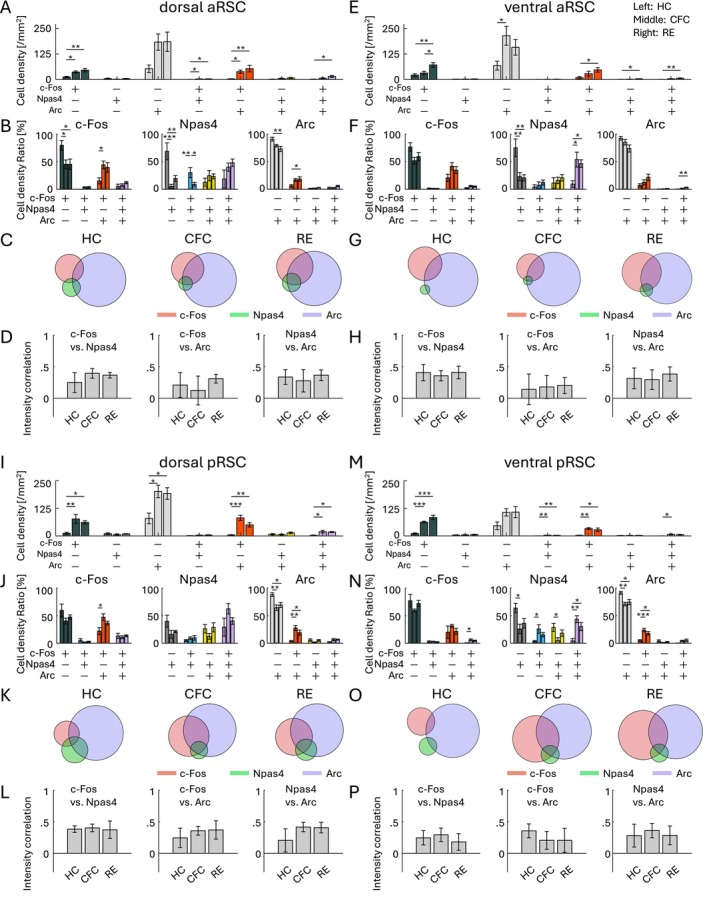
Co‐expression of IEGs in RSC. (A, E, I, M) Cell densities of each cell group with selective or combinative IEG expression in the dorsal aRSC (A), ventral aRSC (E), dorsal pRSC (I), and ventral pRSC (M). (B, F, J, N) Ratio of cell densities per all c‐Fos cells (left), Npas4 cells (middle), and Arc cells (right), in the dorsal aRSC (B), ventral aRSC (F), dorsal pRSC (J), and ventral pRSC (N). (C, G, K, O) Venn diagrams of c‐Fos, Npas4, and Arc positive cells in HC, CFC, and RE groups, in the dorsal aRSC (C), ventral aRSC (G), dorsal pRSC (K), and ventral pRSC (O). The size of the circles corresponds to cell densities, normalized by Arc cell density. (D, H, L, P) Average correlation of IEG expression in single cells, between c‐Fos versus Npas4 (left), c‐Fos versus Arc (middle), and Npas4 versus Arc (right), in the dorsal aRSC (D), ventral aRSC (H), dorsal pRSC (L), and ventral pRSC (P). For each group of bars, the left bar indicates HC, the middle indicates CFC, and the right indicates RE.

Finally, we investigated IEG colocalization in the aRSC and pRSC. In the aRSC, the cell density of the c‐Fos/Npas4/Arc triple‐positive cells was significantly increased by RE but not clearly observed by CFC, both in the dorsal and ventral aRSC (Figure 7A,E). The increase in the ratio of the triple‐positive cells was not clear in the dorsal aRSC (Figures 7B,C and S14G), and was only significant within Npas4^+^ and Arc^+^ cells in the ventral aRSC (Figures 7F,G and S14H). The increase in the correlation of c‐Fos, Npas4, and Arc expression levels was not clear in dorsal/ventral aRSC (Figure 7D,H). In the pRSC, the cell density of the c‐Fos/Npas4/Arc triple‐positive cells was significantly increased by CFC and RE in the dorsal pRSC (Figure 7I), and by CFC in the ventral pRSC (Figure 7M). The increase in the ratio of the triple‐positive cells was not significant in the dorsal pRSC (Figures 7J,K and S14I), and was significant within c‐Fos^+^ and Npas4^+^ cells in the ventral pRSC (Figures 7N,O and S14J). The increase of the correlation of c‐Fos, Npas4, and Arc expression levels was not clear in dorsal/ventral pRSC (Figure 7L,P). Therefore, the increase of the c‐Fos/Npas4/Arc triple‐positive cells in the aRSC/pRSC was less clear than in the PFC and BLA, but was distinct from the DG.

We have also investigated expression levels of c‐Fos, Npas4, and Arc in each c‐Fos/Npas4/Arc positive/negative cell group (Figures S15–S17). The increases or decreases in population expression levels were observed, but we could not find systematic tendencies (Figures S18–S20). Also, we could not find systematic tendencies in correlation changes of IEG expression levels in different cell groups (Figures S21–S23). In addition, we investigated the correlation between the cell densities of the different cell groups and freezing duration in CFC (Figure S24) or eating duration in RE (Figure S25), showing positive or negative significant correlations in several brain regions (Figures S24B and S25B).

Altogether, we observed brain area‐specific changes of combinative expression of c‐Fos, Npas4, and Arc induced by CFC or RE (Tables 1 and 2). The degree of combinative IEGs expression increase varied across areas. Interestingly, the DG did not show a clear increase in the c‐Fos/Npas4/Arc triple‐positive ratio after CFC and RE in contrast to the PFC, BLA, and RSC.

**TABLE 1:** Changes in IEG overlapping cells.

Cell density															
CFC								RE							
cfos/npas4/arc	+/−/−	−/+/−	−/−/+	+/+/−	+/−/+	−/+/+	+/+/+	cfos/npas4/arc	+/−/−	−/+/−	−/−/+	+/+/−	+/−/+	−/+/+	+/+/+
PL	↑		↑	↑	↑	↑	↑	PL	↑		↑	↑	↑		↑
IL	↑		↑	↑	↑	↑	↑	IL	↑		↑		↑	↑	↑
aBLA					↑		↑	aBLA					↑		↑
pBLA	↑			↑	↑		↑	pBLA	↑			↑	↑		↑
dDG					↑	↓	↑	dDG					↑	↓	↑
vDG	↑				↑			vDG					↑	↓	
d. aRSC	↑			↑				d. aRSC				↑	↑		↑
v. aRSC			↑					v. aRSC	↑				↑	↑	↑
d. pRSC	↑		↑		↑		↑	d. pRSC	↑		↑		↑		↑
v. pRSC	↑			↑	↑		↑	v. pRSC	↑			↑	↑		

Ratio in each IEG+ population															
cfos+ cell population															
CFC								RC							
cfos/npas4/arc	+/−/−	−/+/−	−/−/+	+/+/−	+/−/+	−/+/+	+/+/+	cfos/npas4/arc	+/−/−	−/+/−	−/−/+	+/+/−	+/−/+	−/+/+	+/+/+
PL	↓	N/A	N/A		↑	N/A	↑	PL	↓	N/A	N/A		↑	N/A	↑
IL	↓	N/A	N/A		↑	N/A	↑	IL	↓	N/A	N/A		↑	N/A	↑
aBLA		N/A	N/A			N/A	↑	aBLA		N/A	N/A			N/A	↑
pBLA		N/A	N/A			N/A	↑	pBLA		N/A	N/A			N/A	↑
dDG		N/A	N/A			N/A		dDG		N/A	N/A		↑	N/A	
vDG		N/A	N/A		↑	N/A	↓	vDG		N/A	N/A		↑	N/A	↓
d. aRSC	↓	N/A	N/A		↑	N/A		d. aRSC	↓	N/A	N/A			N/A	
v. aRSC		N/A	N/A			N/A		v. aRSC		N/A	N/A			N/A	
d. pRSC		N/A	N/A		↑	N/A		d. pRSC		N/A	N/A			N/A	
v. pRSC		N/A	N/A			N/A	↑	v. pRSC		N/A	N/A			N/A	

Npas4+ cell population															
CFC								RC							
cfos/npas4/arc	+/−/−	−/+/−	−/−/+	+/+/−	+/−/+	−/+/+	+/+/+	cfos/npas4/arc	+/−/−	−/+/−	−/−/+	+/+/−	+/−/+	−/+/+	+/+/+
PL	N/A	↓	N/A		N/A	↑	↑	PL	N/A	↓	N/A		N/A	↑	↑
IL	N/A	↓	N/A	↑	N/A		↑	IL	N/A	↓	N/A		N/A		↑
aBLA	N/A	↓	N/A		N/A		↑	aBLA	N/A	↓	N/A		N/A		↑
pBLA	N/A	↓	N/A		N/A		↑	pBLA	N/A	↓	N/A		N/A		↑
dDG	N/A		N/A		N/A	↓	↑	dDG	N/A		N/A		N/A	↓	↑
vDG	N/A		N/A		N/A			vDG	N/A		N/A		N/A	↓	
d. aRSC	N/A	↓	N/A	↑	N/A			d. aRSC	N/A	↓	N/A		N/A		
v. aRSC	N/A	↓	N/A		N/A		↑	v. aRSC	N/A	↓	N/A		N/A		↑
d. pRSC	N/A		N/A		N/A			d. pRSC	N/A		N/A		N/A		
v. pRSC	N/A	↓	N/A	↑	N/A	↓	↑	v. pRSC	N/A		N/A		N/A		↑

Arc+ cell population															
CFC								RC							
cfos/npas4/arc	+/−/−	−/+/−	−/−/+	+/+/−	+/−/+	−/+/+	+/+/+	cfos/npas4/arc	+/−/−	−/+/−	−/−/+	+/+/−	+/−/+	−/+/+	+/+/+
PL	N/A	N/A	↓	N/A	↑	↑	↑	PL	N/A	N/A	↓	N/A	↑	↑	↑
IL	N/A	N/A	↓	N/A	↑		↑	IL	N/A	N/A	↓	N/A	↑		↑
aBLA	N/A	N/A	↓	N/A	↑		↑	aBLA	N/A	N/A	↓	N/A	↑		↑
pBLA	N/A	N/A	↓	N/A	↑		↑	pBLA	N/A	N/A	↓	N/A	↑		↑
dDG	N/A	N/A		N/A		↓		dDG	N/A	N/A		N/A	↑	↓	
vDG	N/A	N/A		N/A	↑	↓		vDG	N/A	N/A	↓	N/A	↑	↓	
d. aRSC	N/A	N/A		N/A				d. aRSC	N/A	N/A	↓	N/A	↑		
v. aRSC	N/A	N/A		N/A				v. aRSC	N/A	N/A		N/A			↑
d. pRSC	N/A	N/A	↓	N/A	↑			d. pRSC	N/A	N/A	↓	N/A	↑		
v. pRSC	N/A	N/A	↓	N/A	↑			v. pRSC	N/A	N/A	↓	N/A	↑		

*Note:* Summary of changes in cell densities of each cell group and cell density ratio per each IEG type, induced by CFC and RE. Arrows indicate *p* < 0.05. Cohen's *d* were > 0.8 for all.

**TABLE 2:** Changes in expression level correlation between IEGs.

Intensity correlation							
CFC				RE			
	c‐Fos vs. Npas4	c‐Fos vs. Arc	Npas4 vs. Arc		c‐Fos vs. Npas4	c‐Fos vs. Arc	Npas4 vs. Arc
PL	↑		↑	PL	↑		↑
IL	↑		↑	IL			↑
aBLA	↑	↑	↑	aBLA	↑	↑	↑
pBLA	↑			pBLA	↑		
dDG				dDG			
vDG				vDG			
d. aRSC				d. aRSC			
v. aRSC				v. aRSC			
d. pRSC				d. pRSC			
v. pRSC				v. pRSC			

*Note:* Summary of changes in expression level correlation between c‐Fos, Npas4, and Arc in individual cells, induced by CFC and RE. Arrows indicate *p* < 0.05. Cohen's *d* were > 0.8 for all.

### 3.4 IEG Expression‐Based Area‐Area Connectivity

**FIGURE 8:**
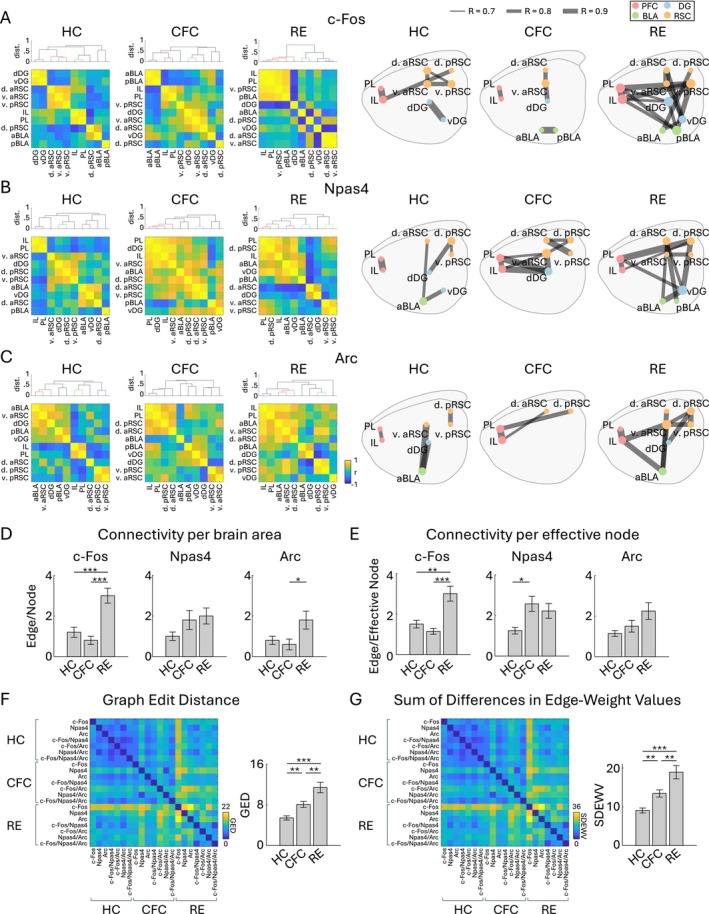
Functional connectivity network of each IEG. (A, B, C) Left, inter‐regional correlation matrices for c‐Fos‐ (A), Npas4‐ (B), and Arc‐ (C) positive cell densities. Dendrograms above the correlation matrices are calculated using dissimilarity index 1 − |*r*|, with colors indicating |*r*| > 0.7 (dissimilarity index < 0.3). Right, connectivity network graphs of c‐Fos (A), Npas4 (B), and Arc (C) generated by connecting each brain region (node) based on the strong correlations (Pearson's |*r*| > 0.7) (right), for HC, CFC, and RE groups. The size of node circles in the network graphs corresponds to the number of connections (edges) the node has. (D, E) Quantification of network complexity. (D) Average number of edges per node across the 10 brain regions. (E) Average number of edges per effective node, the brain region which has at least one connection to another node, across brain regions. (F, G) Quantification of network dissimilarity. (F) Matrix of graph edit distance (GED) across the graphs of different IEG groups. Bar plot indicates average GED within HC, CFC, and RE groups. (G) Matrix of Sum of Differences in Edge‐Weight Values (SDEWV) across the graphs of different IEG groups. Bar plot indicates average SDEWV within HC, CFC, and RE groups (*n* = 7 graphs for each).

Since IEGs have also been employed to examine the functional connectivity between brain areas based on the correlation of IEG expression (Franceschini et al. 2023; Silva et al. 2019; Takeuchi et al. 2022; Tanimizu et al. 2017; Vetere et al. 2017; Wheeler et al. 2013), in this study, we investigated whether the connectivity varies depending on the expression of a given IEG. We calculated Pearson's correlation coefficient of the expression density of c‐Fos, Npas4, Arc positive cells for the brain regions (see Section 2) and generated correlated matrices (Figure 8A–C, left). The connectivity networks were then visualized by applying a threshold (|*R*| > 0.7) to the correlation matrix (Figure 8A–C, right). The connectivity per brain area, or the number of edges per node, was higher in RE than HC in the c‐Fos based network (Figure 8D). Also, the connectivity per effective node, or the number of edges per non‐zero node, was higher in RE and in CFC than HC in the c‐Fos and Npas4 based networks, respectively (Figure 8E). Overall, the connectivity network tended to be more complex by CFC and RE compared with HC (Figure 8A–E). The double−/triple‐IEG‐based networks showed a similar tendency (Figure S26); the connectivity per brain area was larger in CFC in c‐Fos^+^/Npas4^+^ and in RE Npas4^+^/Arc^+^ networks than HC (Figure S26E), and the connectivity per effective node was larger in CFC in c‐Fos^+^/Arc^+^ and Npas4^+^/Arc^+^ networks than HC (Figure S26F). The significant increases in the average correlation were observed in Npas4 and c‐Fos^+^/Arc^+^ based networks (Figure S26G). Comparing the graphs obtained from each cell group, the networks were more dissimilar across each cell group in CFC and RE than HC (Figure 8F,G). Thus, networks of IEG‐based functional connectivity tended to become more complex after experience, but they were not identical between the types of IEG.

## 4 Discussion

In this study, we investigated the expression patterns of three IEGs, c‐Fos, Npas4, and Arc, along with their experience‐induced changes in various brain areas (Figures 2 and 3). The pattern of the combinative expression of those IEGs and their experience‐induced changes was also evaluated (Figures 4, 5, 6, 7). We also demonstrated that different IEG expressions provide different information on area‐area connectivity networks (Figure 8).

We used the method of automated cell detection after background assumption (ADABA) that we proposed previously (Figure 1) (Osanai et al. 2025). This method provides unbiased detection of the immunolabeled cells after reducing the effect of uneven background arising from biological structures in a brain tissue; for example, we often observed uneven background in the DG granule cell layer (Figures S4 and S5). Consistent with our previous report (Osanai et al. 2025), our method provided highly precise cell detection, which aided the subsequent IEG co‐expression analysis.

While several techniques for brain‐wide neural activity mapping using IEGs have been developed in recent years (DeNardo et al. 2019; Franceschini et al. 2025; Guenthner et al. 2013; Nagahama et al. 2025; Renier et al. 2016; Roy et al. 2022; Wheeler et al. 2013; Ye et al. 2016; Zhang and Roy 2024), investigations of differences in IEG types are still limited (Chiaruttini et al. 2025; Heroux et al. 2018; Kawashima et al. 2014; Ons et al. 2004). Also, IEG expression in brain subregions has rarely been investigated comprehensively. In this study, we investigated c‐Fos, Npas4, and Arc expressing cell densities in subregions of the PFC, BLA, DG, and RSC (Figures 2 and 3).

In the PFC, PL and IL subregions have distinct functions on fear expression/extinction and reward‐seeking behaviors (Giustino and Maren 2015; Gourley and Taylor 2016; Sierra‐Mercado et al. 2011). The PL and IL have different c‐Fos protein expression during fear renewal and retrieval of extinguished fear memories (Knapska and Maren 2009), and Arc RNA expression level is higher in the IL in the fear‐extinguished rats but not different in the PL in the fear renewal (Orsini et al. 2013). In contrast, both PL and IL show increased c‐Fos protein expression after fear conditioning (Herry and Mons 2004) and cocaine conditioning (Zavala et al. 2007) as well as increased Arc RNA expression after food conditioning (Schiltz et al. 2007). In this study, we found that the number of c‐Fos, Npas4, and Arc protein positive cells is similarly increased in both PL and IL in response to both fear and reward experience (Figures 3 and S7A,D).

In the BLA, c‐Fos expression is induced by both aversive and appetitive stimuli, which are critical for valence‐related behavior, observed using IHC and in situ hybridization (ISH) (Gore et al. 2015; Redondo et al. 2014). The aBLA and pBLA have neurons with different molecular identities and projections encoding negative and positive valence, respectively (Beyeler et al. 2018; Kim et al. 2016; O'Neill et al. 2018; Pi et al. 2020; Yang and Wang 2017; Zhang et al. 2020, 2021). However, we did not observe such negative and positive valence differences in IEG expression changes between CFC and RE in the BLA, although there were anterior–posterior differences in the type of IEGs whose expression increased; after both CFC and RE, c‐Fos was significantly increased in the aBLA, whereas c‐Fos, Npas4, and Arc were increased in the pBLA (Figures 3 and S7G,J). It has a possibility that our behavior procedure could interfere with distinguishing the effects of aversive and appetitive experiences by novel context exposure. Also, further cell‐type‐specific investigations (Zhang et al. 2021, Zhang et al. 2020) are needed to understand how valence‐specific learning induces multiple IEG expression.

In the DG, dDG supports spatial memory while vDG is involved in fear and reward related behaviors (Kesner 2018; Kheirbek et al. 2013; Kirk et al. 2017). c‐Fos in the DG has been shown to increase by conditioning to fear and reward in IHC (Beck and Fibiger 1995; Rademacher et al. 2006), as well as Arc is increased by fear conditioning in ISH and transgene approach (Bal et al. 2025; Rao‐Ruiz et al. 2019). Also, an increase of Npas4 has been observed with the RAM system (Sun et al. 2020) and RNA level (Bal et al. 2025) after fear conditioning. In this study, we observed an increase of c‐Fos^+^ cells in both the dDG and vDG after fear conditioning and reward experience, and Arc increase in the dDG by reward experience and in the vDG by fear conditioning (Figures 3A and S9A,C). In contrast, we did not observe significant changes in the number of Npas4^+^ cells, which is similar to previous reports exploring Npas4 protein expression using fear conditioning (Chiaruttini et al. 2025; Ramamoorthi et al. 2011) and social interaction (Coutellier et al. 2012). The discrepancy between our Npas4 expression results and the results from the RAM system and RNA level experiments implies that innate expression of Npas4 protein is strictly controlled unlike the RAM system or RNA level, but further investigations are needed.

Finally, the RSC has several subregions with distinct cytoarchitectures, projections, and functions for episodic memory (Alexander et al. 2023; Burwell and Amaral 1998; Cheng et al. 2024; Sugar et al. 2011; Sullivan et al. 2023; Tsai et al. 2022; Vann et al. 2009; Vogt et al. 2004). For example, the anterior part of the RSC is needed for object (de Landeta et al. 2020) and trace‐fear memory (Trask et al. 2021), while the posterior RSC is important for spatial memory (de Landeta et al. 2020; Trask et al. 2021; Vann et al. 2003). Dorsal (dysgranular) and ventral (granular) RSC are suggested to differentially encode allocentric and egocentric information (Alexander and Nitz 2015; Alexander et al. 2023; Jacob et al. 2017; Pothuizen et al. 2009). IEGs including c‐Fos and Arc are increased in the RSC after CFC in IHC (Robinson et al. 2012). In subregional studies, c‐Fos in both aRSC and pRSC is increased by acquisition of CFC memory and recall, whereas another IEG, zif268, is increased only in the aRSC during the recall phase in IHC (Trask and Helmstetter 2022). c‐Fos expression in both dorsal and ventral RSC is increased by fear conditioning in IHC (Radwanska et al. 2010), but has differential expression in a spatial working memory task; c‐Fos increase is observed both in the dorsal (dysgranular) and ventral (granular) RSC with a visual cue, although more clearly in the posterior part, whereas the increase is observed only in the ventral RSC in the dark in IHC (Pothuizen et al. 2009). Supporting subregional differences of the RSC, we found IEG expression differences in the RSC subregions (Figures 3 and S9E,G,I,K). For instance, while c‐Fos was increased by CFC in the dorsal aRSC and in the dorsal/ventral pRSC, the increase was not significant in the ventral aRSC. Also, Npas4 was increased by RE in the dorsal/ventral aRSC, but it was not clear in the dorsal/ventral pRSC. Our multi‐region multi‐IEG analysis supports that the induction of each IEG differs depending on the subregion and may contribute to the distinct memory functions.

We observed the brain region‐dependent enhancement of combinative expression of c‐Fos, Npas4, and Arc following aversive or rewarding experiences (Figures 4, 5, 6, 7). While there are several studies investigating colocalization of different IEGs (Chan et al. 1993; Gonzales et al. 2020; Guldenaar et al. 1994; Guzowski et al. 1999, 2001; Hrvatin et al. 2018; Lonergan et al. 2010; Nakagami et al. 2013; Sheng et al. 1995; Stone et al. 2011; Thompson et al. 2010; Zuniga et al. 2024), comprehensive analysis across brain areas has rarely been conducted. Recently, Chiaruttini et al. developed a pipeline to investigate brain‐wide IEG expression and demonstrated the colocalization of c‐Fos/Npas4 and c‐Fos/Arc in HC, novel context, and CFC using IHC (Chiaruttini et al. 2025). They observed basal co‐expression level in HC varies across brain areas, with high abundance of c‐Fos/Arc colocalization in the DG. They also observed co‐expression of c‐Fos/Arc increased in the DG, BLA, and some cortical areas by novel context exposure and CFC, which is consistent with our results. In DG area analysis, we found that the ratio of triple co‐expressed neurons was different between the dorsal and ventral DG (Figures 6 and S14E,F). In addition, we found that the increase of c‐Fos/Npas4/Arc triple‐expressing cell ratio was less clear in d/vDG compared with the PFC and in BLA unlike the increase of c‐Fos/Arc double co‐expression. This indicates that correlation of different expression varies depending on the combination of co‐expression pattern of IEG types. Thus, although different types of IEGs tend to express in a neuron collaboratively (Figures 4, 5, 6, 7), the degree of co‐expression might depend on the animal's states, brain areas, and IEG types.

Do engram cells tagged by different IEGs or co‐expressed IEGs play different roles in memory? By viral vector approaches, Sun et al. recently found c‐Fos^+^ and Npas4^+^ cell populations labeled by the RAM system in DG have distinct roles in memory generalization and discrimination (Sun et al. 2020). Ye et al. found that Arc^+^/Npas4^+^ cells in PFC are involved in positive‐valence experience but not the entire Arc^+^ or Npas4^+^ populations, as well as the involvement of c‐Fos^+^/Npas4^+^ cells than the entire Fos^+^ population using Npas4‐IHC and Arc‐dependent TRAP mouse or fosCreER virus (Ye et al. 2016). However, this question remains largely open. Although neural activity and IEG expression are strongly coupled, the expression does not simply reflect the level of average neural activity. During new context exploring, only fractions of CA1 place cells are tagged by c‐Fos (Tanaka et al. 2018). In hippocampal culture, c‐Fos expression is induced by synchronized input activity with a preference at 0.1 and 50 Hz, but not solely by raising cAMP, suggesting a relationship with sharp‐wave‐ripples and gamma oscillations (Anisimova et al. 2023; Gee et al. 2024; but see Yang et al. 2024). In contrast, Arc transcription peaks with 10 Hz stimulation, suggesting a relationship with theta oscillations (Kim et al. 2024). Also, the correlation between physiological neural activity and IEG expression is not constant across IEG types: using the FosGFP (Barth et al. 2004) and EGFP‐Arc mice (Okuno et al. 2012), the correlation of neural calcium activity with c‐Fos expression is higher than with Arc in the CA1 (Mahringer et al. 2019) and visual cortex (Mahringer et al. 2022). These suggest that IEG expression may reflect cellular functions including synaptic plasticity than merely indicating neural activity, while c‐Fos and Arc are not always required for the induction of long‐term potentiation (Douglas et al. 1988; Kyrke‐Smith et al. 2021; Wisden et al. 1990). Given that different IEGs play different roles in synaptic plasticity, neurons expressing multiple IEGs could be influenced by the animal's experience more than neurons expressing a single IEG, which may play an important role in contributing to diverse forms of synaptic plasticity in learning. In hippocampal culture, c‐Fos^+^/Arc^+^ cells identified by IHC increase correlated cell firing following chemically induced long‐term potentiation, whereas c‐Fos^−^/Arc^+^ cells decrease correlated cell firing, suggesting that different IEGs and their combination perform distinct functions (Jiang and VanDongen 2021). Neurons in CA1 with high c‐Fos induction show higher correlated activities than neurons with low c‐Fos induction during the spatial learning in the Fos‐GFP mouse (Pettit et al. 2022). Also, Arc positive cells are more likely to participate in sharp‐wave‐ripples than the negative cells in the CA1 acute slices of Arc‐dVenus mice (Norimoto et al. 2018). Conditional knockout of Scg2, the gene activated by c‐Fos, lowers fast‐gamma oscillation power and shifts the preferred theta phase of spikes in CA1 (Yap et al. 2021). Correspondingly, the spikes of CA1 c‐Fos^+^ cells occur during fast gamma events than c‐Fos^−^ cells, and the preferred theta phase of theta‐burst spikes of c‐Fos^+^ cells differs from c‐Fos^−^ spikes (Tanaka et al. 2018). The IEG‐expressing cell assembles form spatially defined clusters in the striatum in ISH (Gonzales et al. 2020). Together, IEG expression and their combinations may reflect ongoing synaptic plasticity which leads to local neural activity synchrony, with the specific form depending on the types of expressing IEGs.

Finally, we analyzed aspects of the functional connectivity network across brain regions based on different IEGs (Figure 8). Since a subpopulation of neurons activated in memory acquisition overlaps with those activated in recall (Josselyn and Tonegawa 2020; Roy et al. 2022), we considered the possibility that the area‐to‐area network between these subpopulations is critical for memory recall and such subnetwork can be visualized by the co‐expression of IEGs. However, we did not observe such correspondence of subnetwork in the double/triple IEG‐based networks, compared with the c‐Fos, Npas4, and Arc single IEG‐based networks (Figures 8A–C and S26A–D). On the other hand, although our correlation analysis used a relatively small sample size (*n* = 6 animals for each group), we found systematic tendencies for the IEG‐based functional connectivity networks to become more complex and more dissimilar between IEGs. It is reported that similar region‐ and IEG type‐dependency in IEG‐expression correlation was observed between the hippocampus, entorhinal cortex, and visual cortex, and between RNA levels of c‐Fos, Arc, and zif268 (Guzowski et al. 2001). These suggest the possibility that the different factors of functional connectivity are coded by the expression of different IEG or their co‐expression. Consistent with our hypothesis, whisker association training does not alter c‐Fos expression in the barrel cortex of the FosGFP mouse, suggesting that the c‐Fos^+^ cells in the sensory cortex can be involved in other functions (Lee et al. 2021). However, although IEG‐based functional connectivity between brain areas has often been estimated (Franceschini et al. 2023; Silva et al. 2019; Takeuchi et al. 2022; Tanimizu et al. 2017; Vetere et al. 2017; Wheeler et al. 2013), direct evidence that supports the link between physiological and IEG‐based connectivity remains scarce and requires a more detailed interpretation of IEG‐based networks, for example investigating whether a specific IEG‐expressing neuron preferentially connects to neurons expressing the same type of IEG.

In this study, we examined the differential or concurrent expression across three IEGs induced by differential experience (CFC or RE). CFC and RE stimulation enhanced those IEG expressions and prompted their connectivity networks (Figures 2, 3, 4, 5, 6, 7, 8). We found that there were some differences in IEG expression after CFC or RE; for example, the increase in the number of Npas4‐positive cells was significant by RE but less clear by CFC in the dorsal and ventral aRSC (Figures 3A and S8E,G), and the functional connectivity tended to be more complex after reward experience than fear conditioning, and the networks were more dissimilar across each cell group after reward experience (Figure 8). These results suggest that those effects are induced by different component stimuli which contain fear‐ or reward‐experience, individually. On the other hand, we observed that the effects of CFC and RE on most IEG expressions and co‐expressions were similar in many cases. We speculate this similarity may be due to the effects of novel environment exposure. Since animals were exposed to a novel context and sensory cues during CFC and RE, the IEGs can be activated by not only aversive and reward stimulations but also exposure to a novel environment, which could interfere with distinguishing the effects of aversive and appetitive experiences. Other behavioral paradigms, for example, novel context exposure or experiments in a familiar environment, are needed to dissociate context and emotional components (Chiaruttini et al. 2025) when we discuss the change of IEGs based on pure fear or reward value. Our one‐trial food rewarding experience procedure is different from previous reports showing rodents rapidly learn associations, for example, associations between context and food with two 30‐min training sessions (Stern et al. 2020) and between flavor and water with one‐trial rewarding procedures (Ackroff et al. 2009). The intensity or type of unconditioned stimulus may also affect IEG expression because different IEGs have different transcription induction thresholds (Abraham et al. 1993; Worley et al. 1993). Also, the co‐expression pattern can differ by observation time: the protein levels of c‐Fos and Arc peak at 60–90 min, and Npas4 peaks at 30–60 min after stimulation, while the mRNA levels of c‐Fos and Arc peak at 30 min and Npas4 peaks at 5 min (Guzowski et al. 2001; Lonergan et al. 2010; Ramamoorthi et al. 2011; Skar et al. 1994; Sun and Lin 2016). Time sensitivity and dynamics of IEG combinative expression need to be investigated. It is also possible that the degree of observed IEG co‐expression rate varies by the sensitivity of antibody probes.

While we investigated the co‐expression patterns of IEGs in multiple brain areas in this study, it would be interesting to investigate whether cells expressing different IEGs in the same subregion have different anatomical long‐range projections because the transcriptions and neural projections can differ depending on emotional valence (Fuentes‐Ramos and Barco 2024; Shpokayte et al. 2022; Ye et al. 2016). In addition, we did not examine layer‐specificity of IEG co‐expression in the current study. Recently, several tools were developed for automated brain atlas registration (Chiaruttini et al. 2025; Franceschini et al. 2025; Roy et al. 2022; Terstege et al. 2022). Development of automated registration techniques based on cytoarchitectures to identify brain subregions and layer structures will accelerate understanding of brain‐wide IEG‐expression patterns. Moreover, cell‐type dependencies for IEG‐expression have been reported (Gonzales et al. 2020; Hochgerner et al. 2023; Jaeger et al. 2018; Lucas et al. 2008; Yang et al. 2022). Single‐cell transcriptomes will help to reveal brain‐wide IEG co‐expression patterns with cell‐type specificity (Chen et al. 2019; Hrvatin et al. 2018; Jovic et al. 2022; Moffitt et al. 2018; Tyssowski et al. 2018; Wu et al. 2017; Yao et al. 2023). However, it should be noted that the levels of RNA and proteins can mismatch due to complex posttranscriptional processes (Alberini and Kandel 2014; Buccitelli and Selbach 2020; Guzowski 2006; Li et al. 2020). These suggest that the IEG‐RNA expression is more relevant to neural activity, while protein expression is more relevant to cellular functions. Further studies are needed to investigate simultaneous recording of neural activity and dynamics of IEG RNA (Lee et al. 2022) and protein synthesis (Meenakshi et al. 2021) to understand whether RNA and protein tag engram cells similarly or not. Also, it is notable that IEG proteins have wide‐range functions beyond affecting synaptic plasticity, including lipid synthesis (Caputto et al. 2014; Rodriguez‐Berdini et al. 2020; Vaughen et al. 2023), DNA repair (Pollina et al. 2023), protection against neuronal death (Rawat et al. 2016), and β‐amyloid generation (Wu et al. 2011). Given this background, it could be considered that various cellular functions can be interpreted from IEG‐tagged engram cells.

In conclusion, we found that basal and experience‐induced expression of c‐Fos, Npas4, Arc, and their combinations vary across different brain areas. The results of IEG‐based connectivity analysis suggest that different functional connectivity is coded by the expression of different IEGs or their co‐expression. These findings provide insights that engram cells also can be differently identified depending on the types and the combinations of IEGs. Further investigations are needed to understand whether interactions between different IEGs contribute unique roles in memory, to gain a more detailed functional understanding and interpretation of IEG‐tagged engram cells.

## Author Contributions

H.O., T.K., and S.K.O. contributed to the study design. M.A. and H.O. conducted experiments. H.O. conducted all analyses, M.A. conducted manual cell detection, K.E.G. conducted behavioral analysis, and C.C.S. visualized the network graph. H.O., T.K., and S.K.O. wrote the manuscript. All authors approved the final manuscript.

## Conflicts of Interest

The authors declare no conflicts of interest.

## Supporting information


**Figure S1:** Correlation of automatically and manually detected cell number. Correlation of automatically and manually detected cell number, same with Figure 1C but shown separately.
**Figure S2:** IEG expression in PFC. (A, B) Larger field‐of‐view images of the PL (A) and IL (B). White dashed line indicates the region‐of‐interest (ROI) of each subregion used for automated cell detection analysis. Scale bars, 400 μm.
**Figure S3:** IEG expression in BLA. (A, B) Larger field‐of‐view images of the aBLA (A) and pBLA (B). White dashed line indicates the ROI of each subregion. Scale bars, 200 μm.
**Figure S4:** IEG expression in dDG. Larger field‐of‐view images of the dDG. White dashed line indicates the ROI. Scale bars, 200 μm. Uneven background was observed as the darker background level around subgranular zone of the granule cell layer in the Npas4 and Arc images.
**Figure S5:** IEG expression in vDG. Larger field‐of‐view images of the vDG. White dashed line indicates the ROI. Scale bars, 400 μm. Uneven background was observed in the c‐Fos images of HC and CFC, and the Arc image of RE, as the increased autofluorescence along the granule cell layer.
**Figure S6:** IEG expression in aRSC. (A) Positions of the dorsal and ventral aRSC in the brain atlas (Allen Institute for Brain Science 2004). (B, C) Larger field‐of‐view images of the dorsal aRSC (B) and ventral aRSC (C). White dashed line indicates the ROI of each subregion. Scale bars, 400 μm.
**Figure S7:** IEG expression in pRSC. (A) Positions of the dorsal and ventral pRSC in the brain atlas (Allen Institute for Brain Science 2004). (B, C) Larger field‐of‐view images of the dorsal pRSC (B) and ventral pRSC (C). White dashed line indicates the ROI of each subregion. Scale bars, 400 μm.
**Figure S8:** Cell density and expression level of IEG‐positive cells in PFC and BLA. (A–C), Analysis in the PL. (A) Cell density of c‐Fos, Npas4, and Arc positive cells in HC, CFC, and RE in the PL. (B) Expression level of c‐Fos, Npas4, and Arc positive cells in HC, CFC, and RE. *n* = 962, 4462, and 3897 cells for HC, CFC, and RE. (C) Percentage of c‐Fos, Npas4, and Arc positive neurons per all NeuN^+^ cells. (D–F), Analysis in the IL. (D) Cell density of c‐Fos, Npas4, and Arc positive cells in HC, CFC, and RE in IL. (E) Expression level of c‐Fos, Npas4, and Arc positive cells in HC, CFC, and RE. *n* = 1228, 5058, and 4935 cells for HC, CFC, and RE. (F) Percentage of c‐Fos, Npas4, and Arc positive neurons per all NeuN^+^ cells. (G–I), Analysis in the aBLA. (G) Cell density of c‐Fos, Npas4, and Arc positive cells in HC, CFC, and RE in the aBLA. (H) Expression level of c‐Fos, Npas4, and Arc positive cells in HC, CFC, and RE. *n* = 585, 987, and 1031 cells for HC, CFC, and RE. (I) Percentage of c‐Fos, Npas4, and Arc positive neurons per all NeuN^+^ cells. (J–L), Analysis in the pBLA. (J) Cell density of c‐Fos, Npas4, and Arc positive cells in HC, CFC, and RE in pBLA. (K) Expression level of c‐Fos, Npas4, and Arc positive cells in HC, CFC, and RE. *n* = 437, 1110, and 1133 cells for HC, CFC, and RE. (L) Percentage of c‐Fos, Npas4, and Arc positive neurons per all NeuN^+^ cells.
**Figure S9:** Cell density and expression of IEG‐positive cells in DG and RSC. (A, B), Analysis in the dDG. (A) Cell density of c‐Fos, Npas4, and Arc positive cells in HC, CFC, and RE in the dDG. (B) Expression level of c‐Fos, Npas4, and Arc positive cells in HC, CFC, and RE. *n* = 1413, 1542, and 1628 cells for HC, CFC, and RE. (C, D), Analysis in the vDG. (C) Cell density of c‐Fos, Npas4, and Arc positive cells in HC, CFC, and RE in vDG. (D) Expression level of c‐Fos, Npas4, and Arc positive cells in HC, CFC, and RE. *n* = 749, 1406, and 986 cells for HC, CFC, and RE. (E, F), Analysis in the dorsal aRSC. (E) Cell density of c‐Fos, Npas4, and Arc positive cells in HC, CFC, and RE in the dorsal aRSC. (F) Expression level of c‐Fos, Npas4, and Arc positive cells in HC, CFC, and RE. *n* = 783, 2103, and 2552 cells for HC, CFC, and RE. (G, H), Analysis in the ventral aRSC. (G) Cell density of c‐Fos, Npas4, and Arc positive cells in HC, CFC, and RE in the ventral aRSC. (H) Expression level of c‐Fos, Npas4, and Arc positive cells in HC, CFC, and REC. *n* = 1276, 2888, and 3068 cells for HC, CFC, and RE. (I, J), Analysis in dorsal pRSC. (I) Cell density of c‐Fos, Npas4, and Arc positive cells in HC, CFC, and RE in the dorsal pRSC. (J) Expression level of c‐Fos, Npas4, and Arc positive cells in HC, CFC, and RE. *n* = 1458, 2724, and 2102 cells for HC, CFC, and RE. (K, L), Analysis in the ventral pRSC. (K) Cell density of c‐Fos, Npas4, and Arc positive cells in HC, CFC, and RE in the ventral pRSC. (L) Expression level of c‐Fos, Npas4, and Arc positive cells in HC, CFC, and RE. *n* = 1955, 3931, and 4149 cells for HC, CFC, and RE.
**Figure S10:** Effect size of cell density and intensity. (A) Bar plots of Cohen's *d* of cell densities across brain regions, calculated from the data shown in Figures S8A,D,G,J and S9A,C,E,G,I,K. Gray dashed lines indicate the effects are small (*d* = ±0.2), medium (*d* = ±0.5), and large (*d* = ±0.8). (B) Bar plots of Cliff's delta of IEG intensities across brain regions, calculated from the data shown in Figures S8B,E,H,K and S9B,D,F,H,J,L. Gray dashed lines indicate the effects are small (*δ* = ±0.147), medium (*δ* = ±0.33), and large (*δ* = ±0.474).
**Figure S11:** Correlation between c‐Fos‐, Npas4‐, and Arc‐positive cells and freezing behavior in CFC. (A) Schema of CFC. (B) A trace of freezing behavior during the habituation period (1–3 min) and the shock period (3–6 min). The 0.75 mA, 2‐s foot shocks occurred at the 3‐, 4‐, and 5‐min time points, as indicated by vertical orange lines. (C) Freezing rate during habituation and shock periods, with significant test using paired *t*‐test. (D) Scatter plots showing the relationship between freezing ratio during the shock period and c‐Fos, Npas4, and Arc cell densities in each brain region. Each dot represents data from an individual mouse. (E) Pearson's correlation coefficients between c‐Fos, Npas4, and Arc cell densities and freezing ratio during the shock period. The asterisk indicates significance of Pearson's *R*.
**Figure S12:** Correlation between c‐Fos‐, Npas4‐, and Arc‐positive cells and eating behavior in RE. (A) Schema of RE. (B) A trace of eating behavior during the 30‐min RE session. (C) Total eating duration within the 30‐min RE session. (D) Scatter plots showing the relationship between eating duration and c‐Fos, Npas4, and Arc cell densities in each brain region. Each dot represents data from an individual mouse. (E) Pearson's correlation coefficients between c‐Fos, Npas4, and Arc cell densities and eating duration. ** indicates significance of Pearson's *R*.
**Figure S13:** Cell density changes in each IEG in different brain regions. (A) Scatter plots of fold‐changes of c‐Fos, Npas4, and Arc positive cell densities by CFC across 10 brain regions, obtained from Figure 3A. (B) Similarly, scatter plots of fold‐changes of c‐Fos, Npas4, and Arc positive cell densities by RE, obtained from Figure 3A. Gray dashed lines indicate linear regression line. R and p indicate values of Pearson correlation.
**Figure S14:** Cell density ratio per all IEG positive cells in each cell group. Ratio of cell densities per all IEG positive cells. For each group of bars, the left bar indicates HC, the middle bar indicates CFC, and the right bar indicates RE groups. (A) PL, (B) IL, (C) aBLA, (D) pBLA, (E) dDG, (F) vDG, (G) dorsal aRSC, (H) ventral aRSC, (I) dorsal pRSC, and (J) ventral pRSC.
**Figure S15:** Intensities of IEGs in individual cells in PFC and BLA. Scatter plots showing the intensities of c‐Fos, Npas4, and Arc in single cells. (A) PL, (B) IL, (C) aBLA, and (D) pBLA. Colors represent cells with selective or concurrent expression of c‐Fos, Npas4, and Arc. Gray dashed lines indicate correlations of cells across all groups. Intensities within each cell group are shown in Figure S18. Correlation coefficients in each cell group are shown in Figure S21.
**Figure S16:** Intensities of IEGs in individual cells in DG. Scatter plots showing the intensities of c‐Fos, Npas4, and Arc in single cells. (A) dDG and (B) vDG. Colors represent cells with selective or concurrent expression of c‐Fos, Npas4, and Arc. Gray dashed lines indicate correlations of cells across all groups. Intensities within each cell group are shown in Figure S19. Correlation coefficients in each cell group are shown in Figure S22.
**Figure S17:** Intensities of IEGs in individual cells in RSC. Scatter plots showing the intensities of c‐Fos, Npas4, and Arc in single cells. (A) dorsal aRSC, (B) ventral aRSC, (C) dorsal pRSC, and (D) ventral pRSC. Colors represent cells with selective or concurrent expression of c‐Fos, Npas4, and Arc. Gray dashed lines indicate correlations of cells across all groups. Intensities within each cell group are shown in Figure S20. Correlation coefficients in each cell group are shown in Figure S23.
**Figure S18:** Intensities of IEGs in each cell group in PFC and BLA. Intensities of c‐Fos, Npas4, and Arc in individual cells in HC, CFC, and RE, in the PL (A), IL (B), aBLA (C), and pBLA (D).
**Figure S19:** Intensities of IEGs in each cell group in DG. Intensities of c‐Fos, Npas4, and Arc in individual cells in HC, CFC, and RE, in the dDG (A) and vDG (B).
**Figure S20:** Intensities of IEGs in each cell group in RSC. Intensities of c‐Fos, Npas4, and Arc in individual cells in HC, CFC, and RE, in the dorsal aRSC (A), ventral aRSC (B), dorsal pRSC (C), and ventral pRSC (D).
**Figure S21:** Correlation of IEG intensities in each cell group in PFC and BLA. Average correlation of IEG intensities of c‐Fos, Npas4, and Arc in individual cells in HC, CFC, and RE, in the PL (A), IL (B), aBLA (C), and pBLA (D).
**Figure S22:** Correlation of IEG intensities in each cell group in DG. Average correlation of IEG intensities of c‐Fos, Npas4, and Arc in individual cells in HC, CFC, and RE, in the dDG (A) and vDG (B).
**Figure S23:** Correlation of IEG intensities in each cell group in RSC. Average correlation of IEG intensities of c‐Fos, Npas4, and Arc in individual cells in HC, CFC, and RE, in the dorsal aRSC (A), ventral aRSC (B), dorsal pRSC (C), and ventral pRSC (D).
**Figure S24:** Correlation between each cell group and freezing behavior. (A) Scatter plots showing the relationship between freezing ratio during the shock period of CFC (see Figure S11) and the cell density of each cell group in each brain region. Each dot represents data from an individual mouse. (B) Pearson's correlation coefficients between the cell density of each cell group and freezing ratio during the shock period. * indicates significance of Pearson's *R*.
**Figure S25:** Correlation between each cell group and eating behavior. (A) Scatter plots showing the relationship between eating duration during RE sessions (see Figure S12) and the cell density of each cell group in each brain region. Each dot represents data from an individual mouse. (B) Pearson's correlation coefficients between the cell density of each cell group and eating duration. * and ** indicate significance of Pearson's *R*.
**Figure S26:** Functional connectivity network of IEG overlapping cells. (A–D) Similarly to Figure 8, Inter‐regional correlation matrices and connectivity networks based on c‐Fos^+^/Npas4^+^ (A), c‐Fos^+^/Arc^+^ (B), Npas4^+^/Arc^+^ (C), and c‐Fos^+^/Npas4^+^/Arc^+^ cells (D). (E, F) Quantification of network complexity: Average number of edges per node (E) and Average number of edges per effective node (F). (G) Average of absolute correlation values in the correlation matrices for each cell group which are shown in Figures 8A–C and S26A–D.

## Data Availability

The data that support the findings of this study are available from the corresponding author upon reasonable request.

## References

[hipo70030-bib-0001] Abraham, W. C. , S. E. Mason , J. Demmer , et al. 1993. “Correlations Between Immediate‐Early Gene Induction and the Persistence of Long‐Term Potentiation.” Neuroscience 56: 717–727. 8255430 10.1016/0306-4522(93)90369-q

[hipo70030-bib-0002] Ackroff, K. , C. Dym , Y. M. Yiin , and A. Sclafani . 2009. “Rapid Acquisition of Conditioned Flavor Preferences in Rats.” Physiology & Behavior 97: 406–413. 19303888 10.1016/j.physbeh.2009.03.014 PMC2683915

[hipo70030-bib-0003] Alberini, C. M. , and E. R. Kandel . 2014. “The Regulation of Transcription in Memory Consolidation.” Cold Spring Harbor Perspectives in Biology 7: a021741. 25475090 10.1101/cshperspect.a021741 PMC4292167

[hipo70030-bib-0004] Alexander, A. S. , and D. A. Nitz . 2015. “Retrosplenial Cortex Maps the Conjunction of Internal and External Spaces.” Nature Neuroscience 18: 1143–1151. 26147532 10.1038/nn.4058

[hipo70030-bib-0005] Alexander, A. S. , R. Place , M. J. Starrett , E. R. Chrastil , and D. A. Nitz . 2023. “Rethinking Retrosplenial Cortex: Perspectives and Predictions.” Neuron 111: 150–175. 36460006 10.1016/j.neuron.2022.11.006 PMC11709228

[hipo70030-bib-0006] Allen Institute for Brain Science . 2004. Allen Mouse Brain Atlas. https://mouse.brain‐map.org/static/atlas. Allen Institute for Brain Science.

[hipo70030-bib-0007] Anisimova, M. , P. J. Lamothe‐Molina , A. Franzelin , et al. 2023. “Neuronal FOS Reports Synchronized Activity of Presynaptic Neurons.” bioRxiv Preprints. 2023.09.04.556168.

[hipo70030-bib-0008] Asok, A. , F. Leroy , J. B. Rayman , and E. R. Kandel . 2019. “Molecular Mechanisms of the Memory Trace.” Trends in Neurosciences 42: 14–22. 30391015 10.1016/j.tins.2018.10.005 PMC6312491

[hipo70030-bib-0009] Bai, Y. S. , H. Ding , S. Bian , T. Chen , Y. Z. Sun , and W. Wang . 2019. “SimGNN: A Neural Network Approach to Fast Graph Similarity Computation.” In Proceedings of the Twelfth ACM International Conference on Web Search and Data Mining (WSDM'19), 384–392. ACM Digital Library.

[hipo70030-bib-0010] Bal, N. , A. Beletskiy , M. Volobueva , V. Ivanova , and A. Shvadchenko . 2025. “Changes in Gene Expression in Dorsal and Ventral CA1 Areas and Dentate Gyrus in Male Mice Hippocampus due to Context Fear Conditioning.” Neuroscience and Behavioral Physiology 55: 74–85.

[hipo70030-bib-0011] Barondes, S. H. , and M. E. Jarvik . 1964. “The Influence of Actinomycin‐D on Brain RNA Synthesis and on Memory.” Journal of Neurochemistry 11: 187–195. 14166283 10.1111/j.1471-4159.1964.tb06128.x

[hipo70030-bib-0012] Barth, A. L. , R. C. Gerkin , and K. L. Dean . 2004. “Alteration of Neuronal Firing Properties After In Vivo Experience in a FosGFP Transgenic Mouse.” Journal of Neuroscience 24: 6466–6475. 15269256 10.1523/JNEUROSCI.4737-03.2004 PMC6729874

[hipo70030-bib-0013] Beck, C. H. M. , and H. C. Fibiger . 1995. “Conditioned Fear‐Induced Changes in Behavior and in the Expression of the Immediate‐Early Gene C‐Fos – With and Without Diazepam Pretreatment.” Journal of Neuroscience 15: 709–720. 7823174 10.1523/JNEUROSCI.15-01-00709.1995 PMC6578289

[hipo70030-bib-0014] Beyeler, A. , C. J. Chang , M. Silvestre , C. Leveque , P. Namburi , et al. 2018. “Organization of Valence‐Encoding and Projection‐Defined Neurons in the Basolateral Amygdala.” Cell Reports 22: 905–918. 29386133 10.1016/j.celrep.2017.12.097 PMC5891824

[hipo70030-bib-0015] Buccitelli, C. , and M. Selbach . 2020. “mRNAs, Proteins and the Emerging Principles of Gene Expression Control.” Nature Reviews. Genetics 21: 630–644. 10.1038/s41576-020-0258-4 32709985

[hipo70030-bib-0016] Burwell, R. D. , and D. G. Amaral . 1998. “Cortical Afferents of the Perirhinal, Postrhinal, and Entorhinal Cortices of the Rat.” Journal of Comparative Neurology 398: 179–205. 9700566 10.1002/(sici)1096-9861(19980824)398:2<179::aid-cne3>3.0.co;2-y

[hipo70030-bib-0017] Caputto, B. L. , A. M. Cardozo Gizzi , and G. A. Gil . 2014. “C‐Fos: An AP‐1 Transcription Factor With an Additional Cytoplasmic, Non‐Genomic Lipid Synthesis Activation Capacity.” Biochimica et Biophysica Acta 1841: 1241–1246. 24886961 10.1016/j.bbalip.2014.05.007

[hipo70030-bib-0018] Chan, R. K. , E. R. Brown , A. Ericsson , K. J. Kovacs , and P. E. Sawchenko . 1993. “A Comparison of Two Immediate‐Early Genes, c‐Fos and NGFI‐B, as Markers for Functional Activation in Stress‐Related Neuroendocrine Circuitry.” Journal of Neuroscience 13: 5126–5138. 8254363 10.1523/JNEUROSCI.13-12-05126.1993 PMC6576398

[hipo70030-bib-0019] Chen, X. , Y. C. Sun , H. Zhan , J. M. Kebschull , S. Fischer , et al. 2019. “High‐Throughput Mapping of Long‐Range Neuronal Projection Using In Situ Sequencing.” Cell 179: 772–786. 31626774 10.1016/j.cell.2019.09.023 PMC7836778

[hipo70030-bib-0020] Cheng, H. Y. , D. I. Fournier , and T. P. Todd . 2024. “Retrosplenial Cortex and Aversive Conditioning.” Frontiers in Behavioral Neuroscience 18: 1341705. 38983870 10.3389/fnbeh.2024.1341705 PMC11232490

[hipo70030-bib-0021] Chiaruttini, N. , C. Castoldi , L. M. Requie , et al. 2025. “ABBA+ BraiAn, An Integrated Suite for Whole‐Brain Mapping, Reveals Brain‐Wide Differences in Immediate‐Early Genes Induction Upon Learning.” Cell Reports 44: 115876. 40553651 10.1016/j.celrep.2025.115876

[hipo70030-bib-0022] Choi, J. H. , S. E. Sim , J. I. Kim , et al. 2018. “Interregional Synaptic Maps Among Engram Cells Underlie Memory Formation.” Science 360: 430–435. 29700265 10.1126/science.aas9204

[hipo70030-bib-0023] Cliff, N. 1993. “Dominance Statistics: Ordinal Analyses to Answer Ordinal Questions.” Psychological Bulletin 114: 494–509.

[hipo70030-bib-0024] Cohen, J. 1988. Statistical Power Analysis for the Behavioral Sciences. Routledge.

[hipo70030-bib-0025] Coutellier, L. , S. Beraki , P. M. Ardestani , N. L. Saw , and M. Shamloo . 2012. “Npas4: A Neuronal Transcription Factor With a Key Role in Social and Cognitive Functions Relevant to Developmental Disorders.” PLoS One 7: e46604. 23029555 10.1371/journal.pone.0046604 PMC3460929

[hipo70030-bib-0026] Dash, P. K. , B. Hochner , and E. R. Kandel . 1990. “Injection of the Camp‐Responsive Element Into the Nucleus of Aplysia Sensory Neurons Blocks Long‐Term Facilitation.” Nature 345: 718–721. 2141668 10.1038/345718a0

[hipo70030-bib-0027] Davis, H. P. , and L. R. Squire . 1984. “Protein Synthesis and Memory: A Review.” Psychological Bulletin 96: 518–559. 6096908

[hipo70030-bib-0028] de Landeta, A. B. , M. Pereyra , J. H. Medina , and C. Katche . 2020. “Anterior Retrosplenial Cortex Is Required for Long‐Term Object Recognition Memory.” Scientific Reports 10: 4002. 32152383 10.1038/s41598-020-60937-z PMC7062718

[hipo70030-bib-0029] DeNardo, L. A. , C. D. Liu , W. E. Allen , et al. 2019. “Temporal Evolution of Cortical Ensembles Promoting Remote Memory Retrieval.” Nature Neuroscience 22: 460–469. 30692687 10.1038/s41593-018-0318-7 PMC6387639

[hipo70030-bib-0030] Denny, C. A. , M. A. Kheirbek , E. L. Alba , et al. 2014. “Hippocampal Memory Traces Are Differentially Modulated by Experience, Time, and Adult Neurogenesis.” Neuron 83: 189–201. 24991962 10.1016/j.neuron.2014.05.018 PMC4169172

[hipo70030-bib-0031] Douglas, R. M. , M. Dragunow , and H. A. Robertson . 1988. “High‐Frequency Discharge of Dentate Granule Cells, but Not Long‐Term Potentiation, Induces c‐Fos Protein.” Brain Research 464: 259–262. 3145095 10.1016/0169-328x(88)90033-2

[hipo70030-bib-0032] El‐Boustani, S. , J. P. K. Ip , V. Breton‐Provencher , G. W. Knott , H. Okuno , et al. 2018. “Locally Coordinated Synaptic Plasticity of Visual Cortex Neurons In Vivo.” Science 360: 1349–1354. 29930137 10.1126/science.aao0862 PMC6366621

[hipo70030-bib-0033] Fleischmann, A. , O. Hvalby , V. Jensen , et al. 2003. “Impaired Long‐Term Memory and NR2A‐Type NMDA Receptor‐Dependent Synaptic Plasticity in Mice Lacking c‐Fos in the CNS.” Journal of Neuroscience 23: 9116–9122. 14534245 10.1523/JNEUROSCI.23-27-09116.2003 PMC6740829

[hipo70030-bib-0034] Flexner, J. B. , L. B. Flexner , and E. Stellar . 1963. “Memory in Mice as Affected by Intracerebral Puromycin.” Science 141: 57–59. 13945541 10.1126/science.141.3575.57

[hipo70030-bib-0035] Franceschini, A. , M. Jin , C. W. Chen , L. Silvestri , A. Mastrodonato , and C. A. Denny . 2025. “Brain‐Wide Immunolabeling and Tissue Clearing Applications for Engram Research.” Neurobiology of Learning and Memory 218: 108032. 39922482 10.1016/j.nlm.2025.108032 PMC13202242

[hipo70030-bib-0036] Franceschini, A. , G. Mazzamuto , C. Checcucci , et al. 2023. “Brain‐Wide Neuron Quantification Toolkit Reveals Strong Sexual Dimorphism in the Evolution of Fear Memory.” Cell Reports 42: 112908. 37516963 10.1016/j.celrep.2023.112908

[hipo70030-bib-0037] Fuentes‐Ramos, M. , and Á. Barco . 2024. “Unveiling Transcriptional and Epigenetic Mechanisms Within Engram Cells: Insights Into Memory Formation and Stability.” In Engrams: A Window Into the Memory Trace, edited by J. Gräff and S. Ramirez , 111–129. Springer International Publishing. 10.1007/978-3-031-62983-9_7 39008013

[hipo70030-bib-0038] Gee, C. E. , O. M. Constantin , A. Franzelin , and T. G. Oertner . 2024. “Optogenetic Approaches to Study IEG Activation.” In Transcriptional Regulation by Neuronal Activity: To the Nucleus and Back, edited by R. N. Saha and S. M. Dudek , 551–559. Springer Nature Switzerland.

[hipo70030-bib-0039] Giustino, T. F. , and S. Maren . 2015. “The Role of the Medial Prefrontal Cortex in the Conditioning and Extinction of Fear.” Frontiers in Behavioral Neuroscience 9: 298. 26617500 10.3389/fnbeh.2015.00298 PMC4637424

[hipo70030-bib-0040] Gonzales, B. J. , D. Mukherjee , R. Ashwal‐Fluss , Y. Loewenstein , and A. Citri . 2020. “Subregion‐Specific Rules Govern the Distribution of Neuronal Immediate‐Early Gene Induction.” Proceedings of the National Academy of Sciences of the United States of America 117: 23304–23310. 31636216 10.1073/pnas.1913658116 PMC7519336

[hipo70030-bib-0041] Gore, F. , E. C. Schwartz , B. C. Brangers , et al. 2015. “Neural Representations of Unconditioned Stimuli in Basolateral Amygdala Mediate Innate and Learned Responses.” Cell 162: 134–145. 26140594 10.1016/j.cell.2015.06.027 PMC4526462

[hipo70030-bib-0042] Gourley, S. L. , and J. R. Taylor . 2016. “Going and Stopping: Dichotomies in Behavioral Control by the Prefrontal Cortex.” Nature Neuroscience 19: 656–664. 29162973 10.1038/nn.4275 PMC5087107

[hipo70030-bib-0043] Greenberg, M. E. , and E. B. Ziff . 1984. “Stimulation of 3T3 Cells Induces Transcription of the c‐Fos Proto‐Oncogene.” Nature 311: 433–438. 6090941 10.1038/311433a0

[hipo70030-bib-0044] Gu, X. , C. Nardone , N. Kamitaki , A. Mao , S. J. Elledge , and M. E. Greenberg . 2023. “The Midnolin‐Proteasome Pathway Catches Proteins for Ubiquitination‐Independent Degradation.” Science 381: eadh5021. 37616343 10.1126/science.adh5021 PMC10617673

[hipo70030-bib-0045] Guenthner, C. J. , K. Miyamichi , H. H. Yang , H. C. Heller , and L. Luo . 2013. “Permanent Genetic Access to Transiently Active Neurons via TRAP: Targeted Recombination in Active Populations.” Neuron 78: 773–784. 23764283 10.1016/j.neuron.2013.03.025 PMC3782391

[hipo70030-bib-0046] Guldenaar, S. E. , K. Wang , and J. T. McCabe . 1994. “Double Immunofluorescence Staining of Fos and Jun in the Hypothalamus of the Rat.” Cell and Tissue Research 276: 1–6. 8187153 10.1007/BF00354777

[hipo70030-bib-0047] Guzowski, J. F. 2006. “Immediate Early Genes and the Mapping of Environmental Representations in Hippocampal Neural Networks.” In Immediate Early Genes in Sensory Processing, Cognitive Performance and Neurological Disorders, edited by R. Pinaud and L. A. Tremere , 159–176. Springer US.

[hipo70030-bib-0048] Guzowski, J. F. , B. L. McNaughton , C. A. Barnes , and P. F. Worley . 1999. “Environment‐Specific Expression of the Immediate‐Early Gene Arc in Hippocampal Neuronal Ensembles.” Nature Neuroscience 2: 1120–1124. 10570490 10.1038/16046

[hipo70030-bib-0049] Guzowski, J. F. , B. Setlow , E. K. Wagner , and J. L. McGaugh . 2001. “Experience‐Dependent Gene Expression in the Rat Hippocampus After Spatial Learning: A Comparison of the Immediate‐Early Genes Arc, c‐Fos, and zif268.” Journal of Neuroscience 21: 5089–5098. 11438584 10.1523/JNEUROSCI.21-14-05089.2001 PMC6762831

[hipo70030-bib-0050] Heroux, N. A. , B. F. Osborne , L. A. Miller , et al. 2018. “Differential Expression of the Immediate Early Genes c‐Fos, Arc, Egr‐1, and Npas4 During Long‐Term Memory Formation in the Context Preexposure Facilitation Effect (CPFE).” Neurobiology of Learning and Memory 147: 128–138. 29222058 10.1016/j.nlm.2017.11.016 PMC6314028

[hipo70030-bib-0051] Herry, C. , and N. Mons . 2004. “Resistance to Extinction Is Associated With Impaired Immediate Early Gene Induction in Medial Prefrontal Cortex and Amygdala.” European Journal of Neuroscience 20: 781–790. 15255988 10.1111/j.1460-9568.2004.03542.x

[hipo70030-bib-0052] Hochgerner, H. , S. Singh , M. Tibi , et al. 2023. “Neuronal Types in the Mouse Amygdala and Their Transcriptional Response to Fear Conditioning.” Nature Neuroscience 26: 2237–2249. 37884748 10.1038/s41593-023-01469-3 PMC10689239

[hipo70030-bib-0053] Hoffman, G. E. , M. S. Smith , and J. G. Verbalis . 1993. “C‐Fos and Related Immediate‐Early Gene‐Products as Markers of Activity in Neuroendocrine Systems.” Frontiers in Neuroendocrinology 14: 173–213. 8349003 10.1006/frne.1993.1006

[hipo70030-bib-0054] Hrvatin, S. , D. R. Hochbaum , M. A. Nagy , et al. 2018. “Single‐Cell Analysis of Experience‐Dependent Transcriptomic States in the Mouse Visual Cortex.” Nature Neuroscience 21: 120–129. 29230054 10.1038/s41593-017-0029-5 PMC5742025

[hipo70030-bib-0055] Jacob, P. Y. , G. Casali , L. Spieser , H. Page , D. Overington , and K. Jeffery . 2017. “An Independent, Landmark‐Dominated Head‐Direction Signal in Dysgranular Retrosplenial Cortex.” Nature Neuroscience 20: 173–175. 27991898 10.1038/nn.4465 PMC5274535

[hipo70030-bib-0056] Jaeger, B. N. , S. B. Linker , S. L. Parylak , et al. 2018. “A Novel Environment‐Evoked Transcriptional Signature Predicts Reactivity in Single Dentate Granule Neurons.” Nature Communications 9: 3084. 10.1038/s41467-018-05418-8 PMC6079101 30082781

[hipo70030-bib-0057] Jiang, Y. , and A. M. J. VanDongen . 2021. “Selective Increase of Correlated Activity in Arc‐Positive Neurons After Chemically Induced Long‐Term Potentiation in Cultured Hippocampal Neurons.” eNeuro 8: ENEURO.0540‐20.2021. 10.1523/ENEURO.0540-20.2021 PMC8658543 34782348

[hipo70030-bib-0058] Josselyn, S. A. , S. Kohler , and P. W. Frankland . 2015. “Finding the Engram.” Nature Reviews. Neuroscience 16: 521–534. 26289572 10.1038/nrn4000

[hipo70030-bib-0059] Josselyn, S. A. , and S. Tonegawa . 2020. “Memory Engrams: Recalling the Past and Imagining the Future.” Science 367: eaaw4325. 31896692 10.1126/science.aaw4325 PMC7577560

[hipo70030-bib-0060] Jovic, D. , X. Liang , H. Zeng , L. Lin , F. Xu , and Y. Luo . 2022. “Single‐Cell RNA Sequencing Technologies and Applications: A Brief Overview.” Clinical and Translational Medicine 12: e694. 35352511 10.1002/ctm2.694 PMC8964935

[hipo70030-bib-0061] Kandel, E. R. 2001. “The Molecular Biology of Memory Storage: A Dialogue Between Genes and Synapses.” Science 294: 1030–1038. 11691980 10.1126/science.1067020

[hipo70030-bib-0062] Kandel, E. R. , Y. Dudai , and M. R. Mayford . 2014. “The Molecular and Systems Biology of Memory.” Cell 157: 163–186. 24679534 10.1016/j.cell.2014.03.001

[hipo70030-bib-0063] Katche, C. , P. Bekinschtein , L. Slipczuk , et al. 2010. “Delayed Wave of c‐Fos Expression in the Dorsal Hippocampus Involved Specifically in Persistence of Long‐Term Memory Storage.” Proceedings of the National Academy of Sciences of the United States of America 107: 349–354. 20018662 10.1073/pnas.0912931107 PMC2806699

[hipo70030-bib-0064] Kawashima, T. , H. Okuno , and H. Bito . 2014. “A New Era for Functional Labeling of Neurons: Activity‐Dependent Promoters Have Come of Age.” Frontiers in Neural Circuits 8: 37. 24795570 10.3389/fncir.2014.00037 PMC4005930

[hipo70030-bib-0065] Kemp, A. , W. Tischmeyer , and D. Manahan‐Vaughan . 2013. “Learning‐Facilitated Long‐Term Depression Requires Activation of the Immediate Early Gene, c‐, and is Transcription Dependent.” Behavioural Brain Research 254: 83–91. 23644186 10.1016/j.bbr.2013.04.036

[hipo70030-bib-0066] Kesner, R. P. 2018. “An Analysis of Dentate Gyrus Function (An Update).” Behavioural Brain Research 354: 84–91. 28756212 10.1016/j.bbr.2017.07.033

[hipo70030-bib-0067] Kheirbek, M. A. , L. J. Drew , N. S. Burghardt , et al. 2013. “Differential Control of Learning and Anxiety Along the Dorsoventral Axis of the Dentate Gyrus.” Neuron 77: 955–968. 23473324 10.1016/j.neuron.2012.12.038 PMC3595120

[hipo70030-bib-0068] Kim, D. W. , H. C. Moon , B. H. Lee , and H. Y. Park . 2024. “Decoding Arc Transcription: A Live‐Cell Study of Stimulation Patterns and Transcriptional Output.” Learning & Memory 31: a054024. 39260877 10.1101/lm.054024.124 PMC11407692

[hipo70030-bib-0069] Kim, J. , M. Pignatelli , S. Xu , S. Itohara , and S. Tonegawa . 2016. “Antagonistic Negative and Positive Neurons of the Basolateral Amygdala.” Nature Neuroscience 19: 1636–1646. 27749826 10.1038/nn.4414 PMC5493320

[hipo70030-bib-0070] Kirk, R. A. , S. N. Redmon , and R. P. Kesner . 2017. “The Ventral Dentate Gyrus Mediates Pattern Separation for Reward Value.” Behavioral Neuroscience 131: 42–45. 28004952 10.1037/bne0000172

[hipo70030-bib-0071] Kitamura, T. , S. K. Ogawa , D. S. Roy , et al. 2017. “Engrams and Circuits Crucial for Systems Consolidation of a Memory.” Science 356: 73–78. 28386011 10.1126/science.aam6808 PMC5493329

[hipo70030-bib-0072] Knapska, E. , and S. Maren . 2009. “Reciprocal Patterns of c‐Fos Expression in the Medial Prefrontal Cortex and Amygdala After Extinction and Renewal of Conditioned Fear.” Learning & Memory 16: 486–493. 19633138 10.1101/lm.1463909 PMC2726014

[hipo70030-bib-0073] Kwapis, J. L. , T. J. Jarome , J. L. Lee , and F. J. Helmstetter . 2015. “The Retrosplenial Cortex is Involved in the Formation of Memory for Context and Trace Fear Conditioning.” Neurobiology of Learning and Memory 123: 110–116. 26079095 10.1016/j.nlm.2015.06.007 PMC4754129

[hipo70030-bib-0074] Kyrke‐Smith, M. , L. J. Volk , S. F. Cooke , M. F. Bear , R. L. Huganir , and J. D. Shepherd . 2021. “The Immediate Early Gene Arc is Not Required for Hippocampal Long‐Term Potentiation.” Journal of Neuroscience 41: 4202–4211. 33833081 10.1523/JNEUROSCI.0008-20.2021 PMC8143205

[hipo70030-bib-0075] Lee, B. H. , J. Y. Shim , H. C. Moon , et al. 2022. “Real‐Time Visualization of mRNA Synthesis During Memory Formation in Live Mice.” Proceedings of the National Academy of Sciences of the United States of America 119: e2117076119. 35776545 10.1073/pnas.2117076119 PMC9271212

[hipo70030-bib-0076] Lee, J. , J. Urban‐Ciecko , E. Park , et al. 2021. “FosGFP Expression Does Not Capture a Sensory Learning‐Related Engram in Superficial Layers of Mouse Barrel Cortex.” Proceedings of the National Academy of Sciences 118: e2112212118. 10.1073/pnas.2112212118 PMC8719899 34930843

[hipo70030-bib-0077] Li, J. , Y. Zhang , C. Yang , and R. Rong . 2020. “Discrepant mRNA and Protein Expression in Immune Cells.” Current Genomics 21: 560–563. 33414677 10.2174/1389202921999200716103758 PMC7770634

[hipo70030-bib-0078] Lin, Y. , B. L. Bloodgood , J. L. Hauser , et al. 2008. “Activity‐Dependent Regulation of Inhibitory Synapse Development by Npas4.” Nature 455: 1198–1204. 18815592 10.1038/nature07319 PMC2637532

[hipo70030-bib-0079] Liu, X. , S. Ramirez , P. T. Pang , et al. 2012. “Optogenetic Stimulation of a Hippocampal Engram Activates Fear Memory Recall.” Nature 484: 381–385. 22441246 10.1038/nature11028 PMC3331914

[hipo70030-bib-0080] Liu, X. , X. H. Zhu , P. Qiu , and W. Chen . 2012. “A Correlation‐Matrix‐Based Hierarchical Clustering Method for Functional Connectivity Analysis.” Journal of Neuroscience Methods 211: 94–102. 22939920 10.1016/j.jneumeth.2012.08.016 PMC3477851

[hipo70030-bib-0081] Lonergan, M. E. , G. M. Gafford , T. J. Jarome , and F. J. Helmstetter . 2010. “Time‐Dependent Expression of Arc and zif268 After Acquisition of Fear Conditioning.” Neural Plasticity 2010: 139891. 20592749 10.1155/2010/139891 PMC2877205

[hipo70030-bib-0082] Lucas, M. , F. Frenois , C. Vouillac , L. Stinus , M. Cador , and C. Le Moine . 2008. “Reactivity and Plasticity in the Amygdala Nuclei During Opiate Withdrawal Conditioning: Differential Expression of c‐Fos and Arc Immediate Early Genes.” Neuroscience 154: 1021–1033. 18501523 10.1016/j.neuroscience.2008.04.006

[hipo70030-bib-0083] Macbeth, G. , E. Razumiejczyk , and R. D. Ledesma . 2011. “Cliff's Delta Calculator: A Non‐Parametric Effect Size Program for Two Groups of Observations.” Universitas Psychologica 10: 545–555.

[hipo70030-bib-0084] Mahringer, D. , A. V. Petersen , A. Fiser ,et al. 2019. “Expression of c‐Fos and Arc in Hippocampal Region CA1 Marks Neurons That Exhibit Learning‐Related Activity Changes.” bioRxiv Preprints: 644526.

[hipo70030-bib-0085] Mahringer, D. , P. Zmarz , H. Okuno , H. Bito , and G. B. Keller . 2022. “Functional Correlates of Immediate Early Gene Expression in Mouse Visual Cortex.” Peer Community Journal 2: e45. 37091727 10.24072/pcjournal.156 PMC7614465

[hipo70030-bib-0086] Marks, W. D. , J. Yokose , T. Kitamura , and S. K. Ogawa . 2022. “Neuronal Ensembles Organize Activity to Generate Contextual Memory.” Frontiers in Behavioral Neuroscience 16: 805132. 35368306 10.3389/fnbeh.2022.805132 PMC8965349

[hipo70030-bib-0087] Meenakshi, P. , S. Kumar , and J. Balaji . 2021. “In Vivo Imaging of Immediate Early Gene Expression Dynamics Segregates Neuronal Ensemble of Memories of Dual Events.” Molecular Brain 14: 102. 34187543 10.1186/s13041-021-00798-3 PMC8243579

[hipo70030-bib-0088] Meissel, K. , and E. Yao . 2024. “Using Cliff's Delta as a Non‐Parametric Effect Size Measure: An Accessible Web App and R Tutorial.” Practical Assessment, Research & Evaluation 29: 2.

[hipo70030-bib-0089] Minatohara, K. , M. Akiyoshi , and H. Okuno . 2015. “Role of Immediate‐Early Genes in Synaptic Plasticity and Neuronal Ensembles Underlying the Memory Trace.” Frontiers in Molecular Neuroscience 8: 78. 26778955 10.3389/fnmol.2015.00078 PMC4700275

[hipo70030-bib-0090] Moffitt, J. R. , D. Bambah‐Mukku , S. W. Eichhorn , et al. 2018. “Molecular, Spatial, and Functional Single‐Cell Profiling of the Hypothalamic Preoptic Region.” Science 362: eaau5324. 30385464 10.1126/science.aau5324 PMC6482113

[hipo70030-bib-0091] Morgan, J. I. , D. R. Cohen , J. L. Hempstead , and T. Curran . 1987. “Mapping Patterns of c‐Fos Expression in the Central Nervous System After Seizure.” Science 237: 192–197. 3037702 10.1126/science.3037702

[hipo70030-bib-0092] Nagahama, K. , V. H. Jung , and H. B. Kwon . 2025. “Cutting‐Edge Methodologies for Tagging and Tracing Active Neuronal Coding in the Brain.” Current Opinion in Neurobiology 92: 102997. 40056794 10.1016/j.conb.2025.102997 PMC12162242

[hipo70030-bib-0093] Nakagami, Y. , A. Watakabe , and T. Yamamori . 2013. “Monocular Inhibition Reveals Temporal and Spatial Changes in Gene Expression in the Primary Visual Cortex of Marmoset.” Frontiers in Neural Circuits 7: fncir.2013.00043. 10.3389/fncir.2013.00043 PMC3620563 23576954

[hipo70030-bib-0094] Nakagawa, S. , and I. C. Cuthill . 2007. “Effect Size, Confidence Interval and Statistical Significance: A Practical Guide for Biologists.” Biological Reviews of the Cambridge Philosophical Society 82: 591–605. 17944619 10.1111/j.1469-185X.2007.00027.x

[hipo70030-bib-0095] Nikolaienko, O. , S. Patil , M. S. Eriksen , and C. R. Bramham . 2018. “Arc Protein: A Flexible Hub for Synaptic Plasticity and Cognition.” Seminars in Cell & Developmental Biology 77: 33–42. 28890419 10.1016/j.semcdb.2017.09.006

[hipo70030-bib-0096] Norimoto, H. , K. Makino , M. Gao , et al. 2018. “Hippocampal Ripples Down‐Regulate Synapses.” Science 359: 1524–1527. 29439023 10.1126/science.aao0702

[hipo70030-bib-0097] Okuno, H. 2011. “Regulation and Function of Immediate‐Early Genes in the Brain: Beyond Neuronal Activity Markers.” Neuroscience Research 69: 175–186. 21163309 10.1016/j.neures.2010.12.007

[hipo70030-bib-0098] Okuno, H. , K. Akashi , Y. Ishii , N. Yagishita‐Kyo , K. Suzuki , et al. 2012. “Inverse Synaptic Tagging of Inactive Synapses via Dynamic Interaction of Arc/Arg3.1 With CaMKIIbeta.” Cell 149: 886–898. 22579289 10.1016/j.cell.2012.02.062 PMC4856149

[hipo70030-bib-0099] O'Neill, P. K. , F. Gore , and C. D. Salzman . 2018. “Basolateral Amygdala Circuitry in Positive and Negative Valence.” Current Opinion in Neurobiology 49: 175–183. 29525574 10.1016/j.conb.2018.02.012 PMC6138049

[hipo70030-bib-0100] Ons, S. , O. Martí , and A. Armario . 2004. “Stress‐Induced Activation of the Immediate Early Gene Arc (Activity‐Regulated Cytoskeleton‐Associated Protein) is Restricted to Telencephalic Areas in the Rat Brain: Relationship to c‐Fos mRNA.” Journal of Neurochemistry 89: 1111–1118. 15147503 10.1111/j.1471-4159.2004.02396.x

[hipo70030-bib-0101] Orsini, C. A. , C. Yan , and S. Maren . 2013. “Ensemble Coding of Context‐Dependent Fear Memory in the Amygdala.” Frontiers in Behavioral Neuroscience 7: 199. 24379767 10.3389/fnbeh.2013.00199 PMC3861741

[hipo70030-bib-0102] Ortega‐de San Luis, C. , and T. J. Ryan . 2022. “Understanding the Physical Basis of Memory: Molecular Mechanisms of the Engram.” Journal of Biological Chemistry 298: 101866. 35346687 10.1016/j.jbc.2022.101866 PMC9065729

[hipo70030-bib-0103] Osanai, H. , M. Arai , T. Kitamura , and S. K. Ogawa . 2025. “Automated Detection of c‐Fos‐Expressing Neurons Using Inhomogeneous Background Subtraction in Fluorescent Images.” Neurobiology of Learning and Memory 218: 108035. 39986434 10.1016/j.nlm.2025.108035 PMC12990386

[hipo70030-bib-0104] Osanai, H. , J. Yamamoto , and T. Kitamura . 2023. “Extracting Electromyographic Signals From Multi‐Channel LFPs Using Independent Component Analysis Without Direct Muscular Recording.” Cell Reports Methods 3: 100482. 37426755 10.1016/j.crmeth.2023.100482 PMC10326347

[hipo70030-bib-0105] Otsu, N. 1979. “A Threshold Selection Method From Gray‐Level Histograms.” IEEE Transactions on Systems, Man, and Cybernetics 9: 62–66.

[hipo70030-bib-0106] Pettit, N. L. , E. L. Yap , M. E. Greenberg , and C. D. Harvey . 2022. “Fos Ensembles Encode and Shape Stable Spatial Maps in the Hippocampus.” Nature 609: 327–334. 36002569 10.1038/s41586-022-05113-1 PMC9452297

[hipo70030-bib-0107] Pi, G. , D. Gao , D. Wu , et al. 2020. “Posterior Basolateral Amygdala to Ventral Hippocampal CA1 Drives Approach Behaviour to Exert an Anxiolytic Effect.” Nature Communications 11: 183. 10.1038/s41467-019-13919-3 PMC6954243 31924799

[hipo70030-bib-0108] Plath, N. , O. Ohana , B. Dammermann , et al. 2006. “Arc/Arg3.1 is Essential for the Consolidation of Synaptic Plasticity and Memories.” Neuron 52: 437–444. 17088210 10.1016/j.neuron.2006.08.024

[hipo70030-bib-0109] Pollina, E. A. , D. T. Gilliam , A. T. Landau , et al. 2023. “A NPAS4‐NuA4 Complex Couples Synaptic Activity to DNA Repair.” Nature 614: 732–741. 36792830 10.1038/s41586-023-05711-7 PMC9946837

[hipo70030-bib-0110] Pothuizen, H. H. , M. Davies , M. M. Albasser , J. P. Aggleton , and S. D. Vann . 2009. “Granular and Dysgranular Retrosplenial Cortices Provide Qualitatively Different Contributions to Spatial Working Memory: Evidence From Immediate‐Early Gene Imaging in Rats.” European Journal of Neuroscience 30: 877–888. 19712100 10.1111/j.1460-9568.2009.06881.x

[hipo70030-bib-0111] Rademacher, D. J. , B. Kovacs , F. Shen , T. C. Napier , and G. E. Meredith . 2006. “The Neural Substrates of Amphetamine Conditioned Place Preference: Implications for the Formation of Conditioned Stimulus‐Reward Associations.” European Journal of Neuroscience 24: 2089–2097. 17067306 10.1111/j.1460-9568.2006.05066.x

[hipo70030-bib-0112] Radwanska, A. , W. Debowska , M. Liguz‐Lecznar , A. Brzezicka , M. Kossut , and A. Cybulska‐Klosowicz . 2010. “Involvement of Retrosplenial Cortex in Classical Conditioning.” Behavioural Brain Research 214: 231–239. 20561962 10.1016/j.bbr.2010.05.042

[hipo70030-bib-0113] Ramamoorthi, K. , R. Fropf , G. M. Belfort , et al. 2011. “Npas4 Regulates a Transcriptional Program in CA3 Required for Contextual Memory Formation.” Science 334: 1669–1675. 22194569 10.1126/science.1208049 PMC4038289

[hipo70030-bib-0114] Rao‐Ruiz, P. , J. J. Couey , I. M. Marcelo , et al. 2019. “Engram‐Specific Transcriptome Profiling of Contextual Memory Consolidation.” Nature Communications 10: 2232. 10.1038/s41467-019-09960-x PMC6527697 31110186

[hipo70030-bib-0115] Rawat, V. , W. Goux , M. Piechaczyk , and S. R. D'Mello . 2016. “C‐Fos Protects Neurons Through a Noncanonical Mechanism Involving HDAC3 Interaction: Identification of a 21‐Amino Acid Fragment With Neuroprotective Activity.” Molecular Neurobiology 53: 1165–1180. 25592718 10.1007/s12035-014-9058-1 PMC4731312

[hipo70030-bib-0116] Redondo, R. L. , J. Kim , A. L. Arons , S. Ramirez , X. Liu , and S. Tonegawa . 2014. “Bidirectional Switch of the Valence Associated With a Hippocampal Contextual Memory Engram.” Nature 513: 426–430. 25162525 10.1038/nature13725 PMC4169316

[hipo70030-bib-0117] Reijmers, L. G. , B. L. Perkins , N. Matsuo , and M. Mayford . 2007. “Localization of a Stable Neural Correlate of Associative Memory.” Science 317: 1230–1233. 17761885 10.1126/science.1143839

[hipo70030-bib-0118] Renier, N. , E. L. Adams , C. Kirst , et al. 2016. “Mapping of Brain Activity by Automated Volume Analysis of Immediate Early Genes.” Cell 165: 1789–1802. 27238021 10.1016/j.cell.2016.05.007 PMC4912438

[hipo70030-bib-0119] Robinson, S. , C. E. Poorman , T. J. Marder , and D. J. Bucci . 2012. “Identification of Functional Circuitry Between Retrosplenial and Postrhinal Cortices During Fear Conditioning.” Journal of Neuroscience 32: 12076–12086. 22933791 10.1523/JNEUROSCI.2814-12.2012 PMC3477513

[hipo70030-bib-0120] Rodriguez‐Berdini, L. , G. O. Ferrero , F. Bustos Plonka , A. M. Cardozo Gizzi , C. G. Prucca , et al. 2020. “The Moonlighting Protein c‐Fos Activates Lipid Synthesis in Neurons, an Activity That is Critical for Cellular Differentiation and Cortical Development.” Journal of Biological Chemistry 295: 8808–8818. 32385110 10.1074/jbc.RA119.010129 PMC7324492

[hipo70030-bib-0121] Romano, J. , J. D. Kromrey , J. Coraggio , and J. Skowronek . 2006. “Appropriate Statistics for Ordinal Level Data: Should We Really Be Using *T*‐Test and Cohen's *d* for Evaluating Group Differences on the NSSE and Other Surveys.” Presented at Annual Meeting of the Florida Association of Institutional Research.

[hipo70030-bib-0122] Roy, D. S. , Y. G. Park , M. E. Kim , et al. 2022. “Brain‐Wide Mapping Reveals That Engrams for a Single Memory Are Distributed Across Multiple Brain Regions.” Nature Communications 13: 1799. 10.1038/s41467-022-29384-4 PMC8980018 35379803

[hipo70030-bib-0123] Ryan, T. J. , D. S. Roy , M. Pignatelli , A. Arons , and S. Tonegawa . 2015. “Memory. Engram Cells Retain Memory Under Retrograde Amnesia.” Science 348: 1007–1013. 26023136 10.1126/science.aaa5542 PMC5583719

[hipo70030-bib-0124] Sagar, S. M. , F. R. Sharp , and T. Curran . 1988. “Expression of c‐Fos Protein in Brain: Metabolic Mapping at the Cellular Level.” Science 240: 1328–1331. 3131879 10.1126/science.3131879

[hipo70030-bib-0125] Salery, M. , A. Godino , and E. J. Nestler . 2021. “Drug‐Activated Cells: From Immediate Early Genes to Neuronal Ensembles in Addiction.” Advances in Pharmacology 90: 173–216. 33706932 10.1016/bs.apha.2020.09.006 PMC8439138

[hipo70030-bib-0126] Schiltz, C. A. , Q. Z. Bremer , C. F. Landry , and A. E. Kelley . 2007. “Food‐Associated Cues Alter Forebrain Functional Connectivity as Assessed With Immediate Early Gene and Proenkephalin Expression.” BMC Biology 5: 16. 17462082 10.1186/1741-7007-5-16 PMC1868707

[hipo70030-bib-0127] Schindelin, J. , I. Arganda‐Carreras , E. Frise , et al. 2012. “Fiji: An Open‐Source Platform for Biological‐Image Analysis.” Nature Methods 9: 676–682. 22743772 10.1038/nmeth.2019 PMC3855844

[hipo70030-bib-0128] Sheng, H. Z. , P. X. Lin , and P. G. Nelson . 1995. “Combinatorial Expression of Immediate Early Genes in Single Neurons.” Brain Research. Molecular Brain Research 30: 196–202. 7637571 10.1016/0169-328x(94)00291-l

[hipo70030-bib-0129] Sheng, M. , and M. E. Greenberg . 1990. “The Regulation and Function of c‐Fos and Other Immediate Early Genes in the Nervous System.” Neuron 4: 477–485. 1969743 10.1016/0896-6273(90)90106-p

[hipo70030-bib-0130] Shpokayte, M. , O. McKissick , X. Guan , et al. 2022. “Hippocampal Cells Segregate Positive and Negative Engrams.” Communications Biology 5: 1009. 36163262 10.1038/s42003-022-03906-8 PMC9512908

[hipo70030-bib-0131] Sierra‐Mercado, D. , N. Padilla‐Coreano , and G. J. Quirk . 2011. “Dissociable Roles of Prelimbic and Infralimbic Cortices, Ventral Hippocampus, and Basolateral Amygdala in the Expression and Extinction of Conditioned Fear.” Neuropsychopharmacology 36: 529–538. 20962768 10.1038/npp.2010.184 PMC3005957

[hipo70030-bib-0132] Silva, A. J. , J. H. Kogan , P. W. Frankland , and S. Kida . 1998. “CREB and Memory.” Annual Review of Neuroscience 21: 127–148. 10.1146/annurev.neuro.21.1.127 9530494

[hipo70030-bib-0133] Silva, A. J. , Y. Zhou , T. Rogerson , J. Shobe , and J. Balaji . 2009. “Molecular and Cellular Approaches to Memory Allocation in Neural Circuits.” Science 326: 391–395. 19833959 10.1126/science.1174519 PMC2844777

[hipo70030-bib-0134] Silva, B. A. , A. M. Burns , and J. Graff . 2019. “A c‐Fos Activation Map of Remote Fear Memory Attenuation.” Psychopharmacology 236: 369–381. 30116860 10.1007/s00213-018-5000-y PMC6373197

[hipo70030-bib-0135] Skar, R. , T. H. Larsen , and G. Serck‐Hanssen . 1994. “Regulation of c‐Fos Expression by IGF‐I in Bovine Chromaffin Cells: Desensitization Following Cholinergic Activation.” Molecular and Cellular Endocrinology 106: 213–220. 7895910 10.1016/0303-7207(94)90205-4

[hipo70030-bib-0136] Spiegel, I. , A. R. Mardinly , H. W. Gabel , et al. 2014. “Npas4 Regulates Excitatory‐Inhibitory Balance Within Neural Circuits Through Cell‐Type‐Specific Gene Programs.” Cell 157: 1216–1229. 24855953 10.1016/j.cell.2014.03.058 PMC4089405

[hipo70030-bib-0137] Stern, S. A. , K. R. Doerig , E. P. Azevedo , E. Stoffel , and J. M. Friedman . 2020. “Control of Non‐Homeostatic Feeding in Sated Mice Using Associative Learning of Contextual Food Cues.” Molecular Psychiatry 25: 666–679. 29875477 10.1038/s41380-018-0072-y PMC6281813

[hipo70030-bib-0138] Stone, S. S. , C. M. Teixeira , K. Zaslavsky , et al. 2011. “Functional Convergence of Developmentally and Adult‐Generated Granule Cells in Dentate Gyrus Circuits Supporting Hippocampus‐Dependent Memory.” Hippocampus 21: 1348–1362. 20824726 10.1002/hipo.20845

[hipo70030-bib-0139] Sugar, J. , M. P. Witter , N. van Strien , and N. Cappaert . 2011. “The Retrosplenial Cortex: Intrinsic Connectivity and Connections With the (Para)hippocampal Region in the Rat. An Interactive Connectome.” Frontiers in Neuroinformatics 5: fninf.2011.00007. 10.3389/fninf.2011.00007 PMC3147162 21847380

[hipo70030-bib-0140] Sullivan, K. E. , L. Kraus , M. Kapustina , et al. 2023. “Sharp Cell‐Type‐Identity Changes Differentiate the Retrosplenial Cortex From the Neocortex.” Cell Reports 42: 112206. 36881508 10.1016/j.celrep.2023.112206

[hipo70030-bib-0141] Sun, W. , I. Choi , S. Stoyanov , et al. 2021. “Context Value Updating and Multidimensional Neuronal Encoding in the Retrosplenial Cortex.” Nature Communications 12: 6045. 10.1038/s41467-021-26301-z PMC8523535 34663792

[hipo70030-bib-0142] Sun, X. C. , M. J. Bernstein , M. Z. Meng , S. Y. Rao , A. T. Sorensen , et al. 2020. “Functionally Distinct Neuronal Ensembles Within the Memory Engram.” Cell 181: 410. 32187527 10.1016/j.cell.2020.02.055 PMC7166195

[hipo70030-bib-0143] Sun, X. C. , and Y. X. Lin . 2016. “Npas4: Linking Neuronal Activity to Memory.” Trends in Neurosciences 39: 264–275. 26987258 10.1016/j.tins.2016.02.003 PMC4818711

[hipo70030-bib-0144] Takeuchi, T. , M. Tamura , D. Tse , Y. Kajii , G. Fernández , and R. G. M. Morris . 2022. “Brain Region Networks for the Assimilation of New Associative Memory Into a Schema.” Molecular Brain 15: 24. 35331310 10.1186/s13041-022-00908-9 PMC8943948

[hipo70030-bib-0145] Tanaka, K. Z. , H. He , A. Tomar , K. Niisato , A. J. Y. Huang , and T. J. McHugh . 2018. “The Hippocampal Engram Maps Experience but Not Place.” Science 361: 392–397. 30049878 10.1126/science.aat5397

[hipo70030-bib-0146] Tanimizu, T. , J. W. Kenney , E. Okano , K. Kadoma , P. W. Frankland , and S. Kida . 2017. “Functional Connectivity of Multiple Brain Regions Required for the Consolidation of Social Recognition Memory.” Journal of Neuroscience 37: 4103–4116. 28292834 10.1523/JNEUROSCI.3451-16.2017 PMC6596582

[hipo70030-bib-0147] Tantardini, M. , F. Ieva , L. Tajoli , and C. Piccardi . 2019. “Comparing Methods for Comparing Networks.” Scientific Reports 9: 17557. 31772246 10.1038/s41598-019-53708-y PMC6879644

[hipo70030-bib-0148] Terranova, J. I. , J. Yokose , H. Osanai , W. D. Marks , J. Yamamoto , et al. 2022. “Hippocampal‐Amygdala Memory Circuits Govern Experience‐Dependent Observational Fear.” Neuron 110: 1416–1431. 35139362 10.1016/j.neuron.2022.01.019 PMC9035063

[hipo70030-bib-0149] Terranova, J. I. , J. Yokose , H. Osanai , S. K. Ogawa , and T. Kitamura . 2023. “Systems Consolidation Induces Multiple Memory Engrams for a Flexible Recall Strategy in Observational Fear Memory in Male Mice.” Nature Communications 14: 3976. 10.1038/s41467-023-39718-5 PMC10322999 37407567

[hipo70030-bib-0150] Terstege, D. J. , D. O. Oboh , and J. R. Epp . 2022. “FASTMAP: Open‐Source Flexible Atlas Segmentation Tool for Multi‐Area Processing of Biological Images.” eNeuro 9: ENEURO.0325‐21.2022. 10.1523/ENEURO.0325-21.2022 PMC8938980 35228311

[hipo70030-bib-0151] Thomas, J. R. , W. Salazar , and D. M. Landers . 1991. “What is Missing in P‐Less‐Than‐.05 – Effect Size.” Research Quarterly for Exercise and Sport 62: 344–348. 1925064 10.1080/02701367.1991.10608733

[hipo70030-bib-0152] Thompson, C. L. , J. P. Wisor , C. K. Lee , et al. 2010. “Molecular and Anatomical Signatures of Sleep Deprivation in the Mouse Brain.” Frontiers in Neuroscience 4: 165. 21088695 10.3389/fnins.2010.00165 PMC2981377

[hipo70030-bib-0153] Tonegawa, S. , X. Liu , S. Ramirez , and R. Redondo . 2015. “Memory Engram Cells Have Come of Age.” Neuron 87: 918–931. 26335640 10.1016/j.neuron.2015.08.002

[hipo70030-bib-0154] Trask, S. , and F. J. Helmstetter . 2022. “Unique Roles for the Anterior and Posterior Retrosplenial Cortices in Encoding and Retrieval of Memory for Context.” Cerebral Cortex 32: 3602–3610. 35029643 10.1093/cercor/bhab436 PMC9433420

[hipo70030-bib-0155] Trask, S. , S. E. Pullins , N. C. Ferrara , and F. J. Helmstetter . 2021. “The Anterior Retrosplenial Cortex Encodes Event‐Related Information and the Posterior Retrosplenial Cortex Encodes Context‐Related Information During Memory Formation.” Neuropsychopharmacology 46: 1386–1392. 33580135 10.1038/s41386-021-00959-x PMC8134488

[hipo70030-bib-0156] Tsai, T. C. , T. H. Yu , Y. C. Hung , L. I. Fong , and K. S. Hsu . 2022. “Distinct Contribution of Granular and Agranular Subdivisions of the Retrosplenial Cortex to Remote Contextual Fear Memory Retrieval.” Journal of Neuroscience 42: 877–893. 34876468 10.1523/JNEUROSCI.1303-21.2021 PMC8808736

[hipo70030-bib-0157] Tyssowski, K. M. , N. R. DeStefino , J. H. Cho , C. J. Dunn , R. G. Poston , et al. 2018. “Different Neuronal Activity Patterns Induce Different Gene Expression Programs.” Neuron 98: 530–546. 29681534 10.1016/j.neuron.2018.04.001 PMC5934296

[hipo70030-bib-0158] Uytiepo, M. , Y. Zhu , E. Bushong , et al. 2025. “Synaptic Architecture of a Memory Engram in the Mouse Hippocampus.” Science 387: eado8316. 40112060 10.1126/science.ado8316 PMC12233322

[hipo70030-bib-0159] Vann, S. D. , J. P. Aggleton , and E. A. Maguire . 2009. “What Does the Retrosplenial Cortex Do?” Nature Reviews Neuroscience 10: 792–850. 19812579 10.1038/nrn2733

[hipo70030-bib-0160] Vann, S. D. , L. A. Kristina Wilton , J. L. Muir , and J. P. Aggleton . 2003. “Testing the Importance of the Caudal Retrosplenial Cortex for Spatial Memory in Rats.” Behavioural Brain Research 140: 107–118. 12644284 10.1016/s0166-4328(02)00274-7

[hipo70030-bib-0161] Vaughen, J. P. , E. Theisen , and T. R. Clandinin . 2023. “From Seconds to Days: Neural Plasticity Viewed Through a Lipid Lens.” Current Opinion in Neurobiology 80: 102702. 36965206 10.1016/j.conb.2023.102702

[hipo70030-bib-0162] Vedder, L. C. , A. M. P. Miller , M. B. Harrison , and D. M. Smith . 2017. “Retrosplenial Cortical Neurons Encode Navigational Cues, Trajectories and Reward Locations During Goal Directed Navigation.” Cerebral Cortex 27: 3713–3723. 27473323 10.1093/cercor/bhw192 PMC6059095

[hipo70030-bib-0163] Vetere, G. , J. W. Kenney , L. M. Tran , F. Xia , P. E. Steadman , et al. 2017. “Chemogenetic Interrogation of a Brain‐Wide Fear Memory Network in Mice.” Neuron 94: 363–374. 28426969 10.1016/j.neuron.2017.03.037

[hipo70030-bib-0164] Vetere, G. , L. M. Tran , S. Moberg , et al. 2019. “Memory Formation in the Absence of Experience.” Nature Neuroscience 22: 933–940. 31036944 10.1038/s41593-019-0389-0 PMC7592289

[hipo70030-bib-0165] Vogt, B. , L. Vogt , and N. Farber . 2004. “Cingulate Cortex and Disease Models.” In The Rat Nervous System, edited by G. Paxinos , 705–727. Elsevier.

[hipo70030-bib-0166] Wang, K. H. , A. Majewska , J. Schummers , et al. 2006. “In Vivo Two‐Photon Imaging Reveals a Role of Arc in Enhancing Orientation Specificity in Visual Cortex.” Cell 126: 389–402. 16873068 10.1016/j.cell.2006.06.038

[hipo70030-bib-0167] Wang, T. , G. L. Lu , J. Liu , and P. Yan . 2019. “Graph‐Based Change Detection for Condition Monitoring of Rotating Machines: Techniques for Graph Similarity.” IEEE Transactions on Reliability 68: 1034–1049.

[hipo70030-bib-0168] Weng, F. J. , R. I. Garcia , S. Lutzu , et al. 2018. “Npas4 Is a Critical Regulator of Learning‐Induced Plasticity at Mossy Fiber‐CA3 Synapses During Contextual Memory Formation.” Neuron 97: 1137–1152. 29429933 10.1016/j.neuron.2018.01.026 PMC5843542

[hipo70030-bib-0169] Wheeler, A. L. , C. M. Teixeira , A. H. Wang , et al. 2013. “Identification of a Functional Connectome for Long‐Term Fear Memory in Mice.” PLoS Computational Biology 9: e1002853. 23300432 10.1371/journal.pcbi.1002853 PMC3536620

[hipo70030-bib-0170] Wills, P. , and F. G. Meyer . 2020. “Metrics for Graph Comparison: A Practitioner's Guide.” PLoS One 15: e0228728. 32050004 10.1371/journal.pone.0228728 PMC7015405

[hipo70030-bib-0171] Wisden, W. , M. L. Errington , S. Williams , et al. 1990. “Differential Expression of Immediate Early Genes in the Hippocampus and Spinal Cord.” Neuron 4: 603–614. 2108708 10.1016/0896-6273(90)90118-y

[hipo70030-bib-0172] Worley, P. F. , R. V. Bhat , J. M. Baraban , C. A. Erickson , B. L. McNaughton , and C. A. Barnes . 1993. “Thresholds for Synaptic Activation of Transcription Factors in Hippocampus: Correlation With Long‐Term Enhancement.” Journal of Neuroscience 13: 4776–4786. 8229198 10.1523/JNEUROSCI.13-11-04776.1993 PMC6576344

[hipo70030-bib-0173] Wu, J. , R. S. Petralia , H. Kurushima , H. Patel , M. Y. Jung , et al. 2011. “Arc/Arg3.1 Regulates an Endosomal Pathway Essential for Activity‐Dependent Beta‐Amyloid Generation.” Cell 147: 615–628. 22036569 10.1016/j.cell.2011.09.036 PMC3207263

[hipo70030-bib-0174] Wu, Y. E. , L. Pan , Y. Zuo , X. Li , and W. Hong . 2017. “Detecting Activated Cell Populations Using Single‐Cell RNA‐Seq.” Neuron 96: 313–329. 29024657 10.1016/j.neuron.2017.09.026

[hipo70030-bib-0175] Yamamoto, N. , W. D. Marks , and T. Kitamura . 2021. “Cell‐Type‐Specific Optogenetic Techniques Reveal Neural Circuits Crucial for Episodic Memories.” Advances in Experimental Medicine and Biology 1293: 429–447. 33398831 10.1007/978-981-15-8763-4_28 PMC8612024

[hipo70030-bib-0176] Yang, D. , Y. Wang , T. Qi , X. Zhang , L. Shen , et al. 2024. “Phosphorylation of Pyruvate Dehydrogenase Inversely Associates With Neuronal Activity.” Neuron 112: 959–971. 38266644 10.1016/j.neuron.2023.12.015 PMC11021214

[hipo70030-bib-0177] Yang, J. , P. Serrano , X. Yin , X. Sun , Y. Lin , and S. X. Chen . 2022. “Functionally Distinct NPAS4‐Expressing Somatostatin Interneuron Ensembles Critical for Motor Skill Learning.” Neuron 110: 3339–3355. 36099920 10.1016/j.neuron.2022.08.018

[hipo70030-bib-0178] Yang, Y. , and J. Z. Wang . 2017. “From Structure to Behavior in Basolateral Amygdala‐Hippocampus Circuits.” Frontiers in Neural Circuits 11: 86. 29163066 10.3389/fncir.2017.00086 PMC5671506

[hipo70030-bib-0179] Yao, Z. , C. T. J. van Velthoven , M. Kunst , et al. 2023. “A High‐Resolution Transcriptomic and Spatial Atlas of Cell Types in the Whole Mouse Brain.” Nature 624: 317–332. 38092916 10.1038/s41586-023-06812-z PMC10719114

[hipo70030-bib-0180] Yap, E. L. , and M. E. Greenberg . 2018. “Activity‐Regulated Transcription: Bridging the Gap Between Neural Activity and Behavior.” Neuron 100: 330–348. 30359600 10.1016/j.neuron.2018.10.013 PMC6223657

[hipo70030-bib-0181] Yap, E. L. , N. L. Pettit , C. P. Davis , et al. 2021. “Bidirectional Perisomatic Inhibitory Plasticity of a Fos Neuronal Network.” Nature 590: 115–121. 33299180 10.1038/s41586-020-3031-0 PMC7864877

[hipo70030-bib-0182] Ye, L. , W. E. Allen , K. R. Thompson , et al. 2016. “Wiring and Molecular Features of Prefrontal Ensembles Representing Distinct Experiences.” Cell 165: 1776–1788. 27238022 10.1016/j.cell.2016.05.010 PMC5708551

[hipo70030-bib-0183] Yokose, J. , W. D. Marks , and T. Kitamura . 2024. “Visuotactile Integration Facilitates Mirror‐Induced Self‐Directed Behavior Through Activation of Hippocampal Neuronal Ensembles in Mice.” Neuron 112: 306–318. 38056456 10.1016/j.neuron.2023.10.022

[hipo70030-bib-0184] Yokose, J. , N. Yamamoto , S. K. Ogawa , and T. Kitamura . 2023. “Optogenetic Activation of Dopamine D1 Receptors in Island Cells of Medial Entorhinal Cortex Inhibits Temporal Association Learning.” Molecular Brain 16: 78. 37964372 10.1186/s13041-023-01065-3 PMC10647136

[hipo70030-bib-0185] Zavala, A. R. , S. Biswas , R. E. Harlan , and J. L. Neisewander . 2007. “Fos and Glutamate AMPA Receptor Subunit Coexpression Associated With Cue‐Elicited Cocaine‐Seeking Behavior in Abstinent Rats.” Neuroscience 145: 438–452. 17276011 10.1016/j.neuroscience.2006.12.038 PMC1876753

[hipo70030-bib-0186] Zhang, X. , W. Guan , T. Yang , et al. 2021. “Genetically Identified Amygdala‐Striatal Circuits for Valence‐Specific Behaviors.” Nature Neuroscience 24: 1586–1600. 34663958 10.1038/s41593-021-00927-0 PMC8556347

[hipo70030-bib-0187] Zhang, X. , J. Kim , and S. Tonegawa . 2020. “Amygdala Reward Neurons Form and Store Fear Extinction Memory.” Neuron 105: 1077–1093. 31952856 10.1016/j.neuron.2019.12.025

[hipo70030-bib-0188] Zhang, Y. , and D. S. Roy . 2024. “Memory Storage in Distributed Engram Cell Ensembles.” In Engrams: A Window Into the Memory Trace, edited by J. Gräff and S. Ramirez , 29–43. Springer International Publishing. 10.1007/978-3-031-62983-9_3 39008009

[hipo70030-bib-0189] Zuniga, A. , J. Han , I. Miller‐Crews , L. A. Agee , H. A. Hofmann , and M. R. Drew . 2024. “Extinction Training Suppresses Activity of Fear Memory Ensembles Across the Hippocampus and Alters Transcriptomes of Fear‐Encoding Cells.” Neuropsychopharmacology 49: 1872–1882. 38877180 10.1038/s41386-024-01897-0 PMC11473549

