# Supplementary material for: Combinative Protein Expression of Immediate Early Genes c‐Fos, Arc, and Npas4 Along Aversive and Appetitive Experience‐Related Neural Networks
Source: Hippocampus. 2025 Aug 8;35(5):e70030. doi: 10.1002/hipo.70030 (PMC12333481; doi:10.1002/hipo.70030)
Supplement: Supplementary file 1 — Figure S1: Correlation of automatically and manually detected cell number. Correlation of automatically and manually detected cell number, same with Figure 1C but shown separately. Figure S2: IEG expression in PFC. (A, B) Larger field‐of‐view images of the PL (A) and IL (B). White dashed line indicates the region‐of‐interest (ROI) of each subregion used for automated cell detection analysis. Scale bars, 400 μm. Figure S3: IEG expression in BLA. (A, B) Larger field‐of‐view images of the aBLA (A) and pBLA (B). White dashed line indicates the ROI of each subregion. Scale bars, 200 μm. Figure S4: IEG expression in dDG. Larger field‐of‐view images of the dDG. White dashed line indicates the ROI. Scale bars, 200 μm. Uneven background was observed as the darker background level around subgranular zone of the granule cell layer in the Npas4 and Arc images. Figure S5: IEG expression in vDG. Larger field‐of‐view images of the vDG. White dashed line indicates the ROI. Scale bars, 400 μm. Uneven background was observed in the c‐Fos images of HC and CFC, and the Arc image of RE, as the increased autofluorescence along the granule cell layer. Figure S6: IEG expression in aRSC. (A) Positions of the dorsal and ventral aRSC in the brain atlas (Allen Institute for Brain Science 2004). (B, C) Larger field‐of‐view images of the dorsal aRSC (B) and ventral aRSC (C). White dashed line indicates the ROI of each subregion. Scale bars, 400 μm. Figure S7: IEG expression in pRSC. (A) Positions of the dorsal and ventral pRSC in the brain atlas (Allen Institute for Brain Science 2004). (B, C) Larger field‐of‐view images of the dorsal pRSC (B) and ventral pRSC (C). White dashed line indicates the ROI of each subregion. Scale bars, 400 μm. Figure S8: Cell density and expression level of IEG‐positive cells in PFC and BLA. (A–C), Analysis in the PL. (A) Cell density of c‐Fos, Npas4, and Arc positive cells in HC, CFC, and RE in the PL. (B) Expression level of c‐Fos, Npas4, and Arc positive cells in [file HIPO-35-0-s001.pdf]

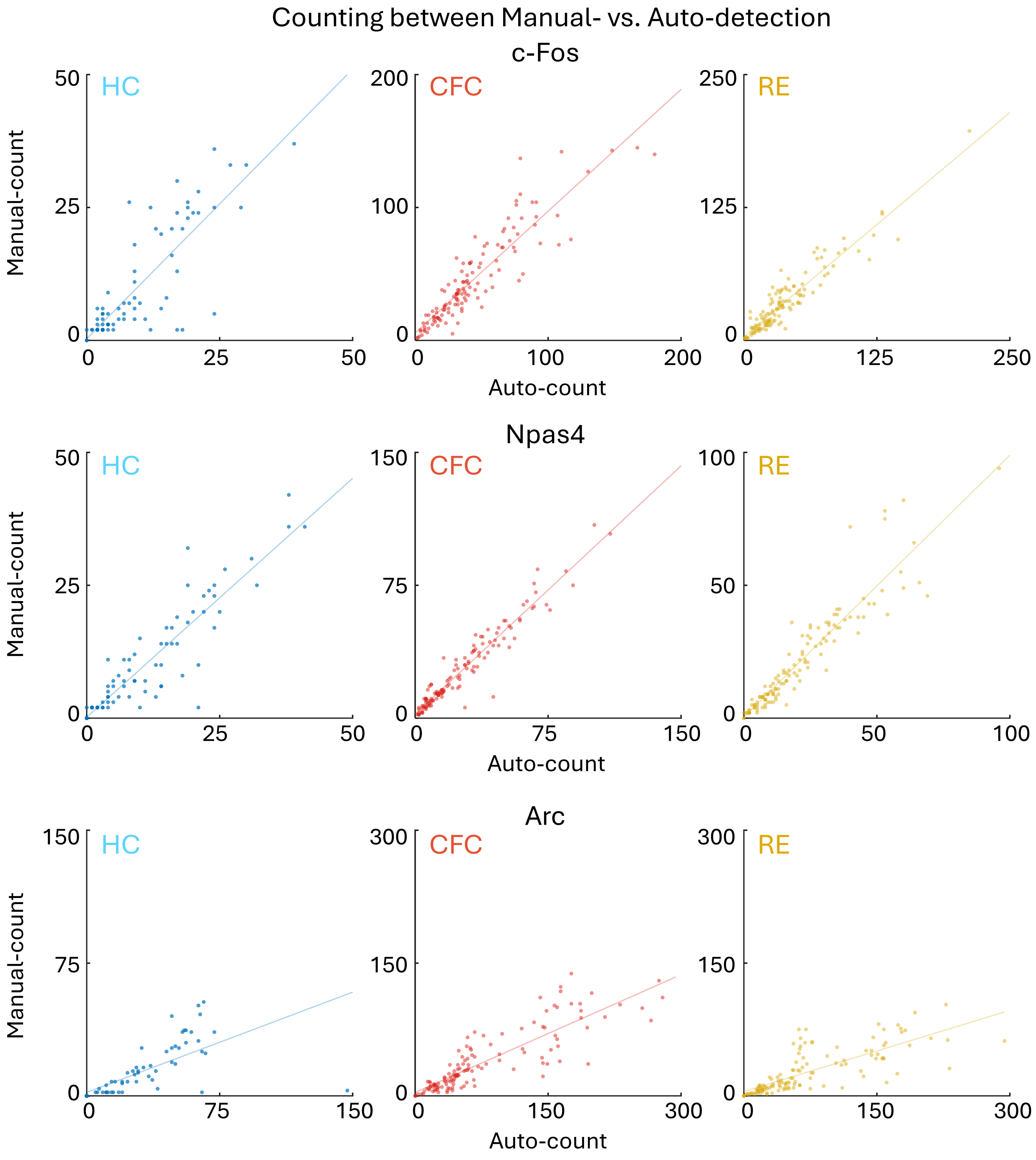

Supp. Figure S1: Correlation of automatically and manually detected cell number

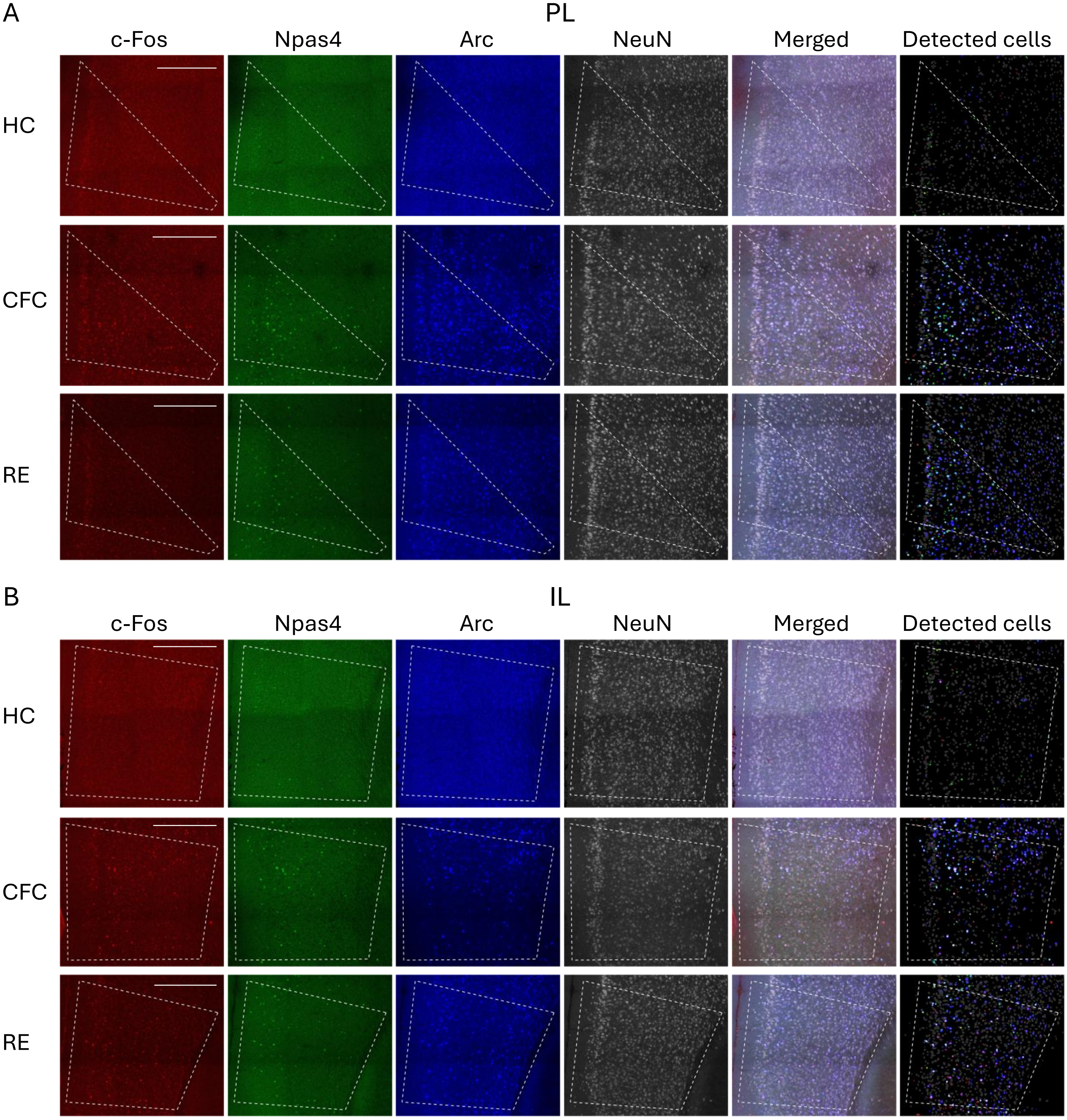

Supp. Figure S2: IEG expression in PFC

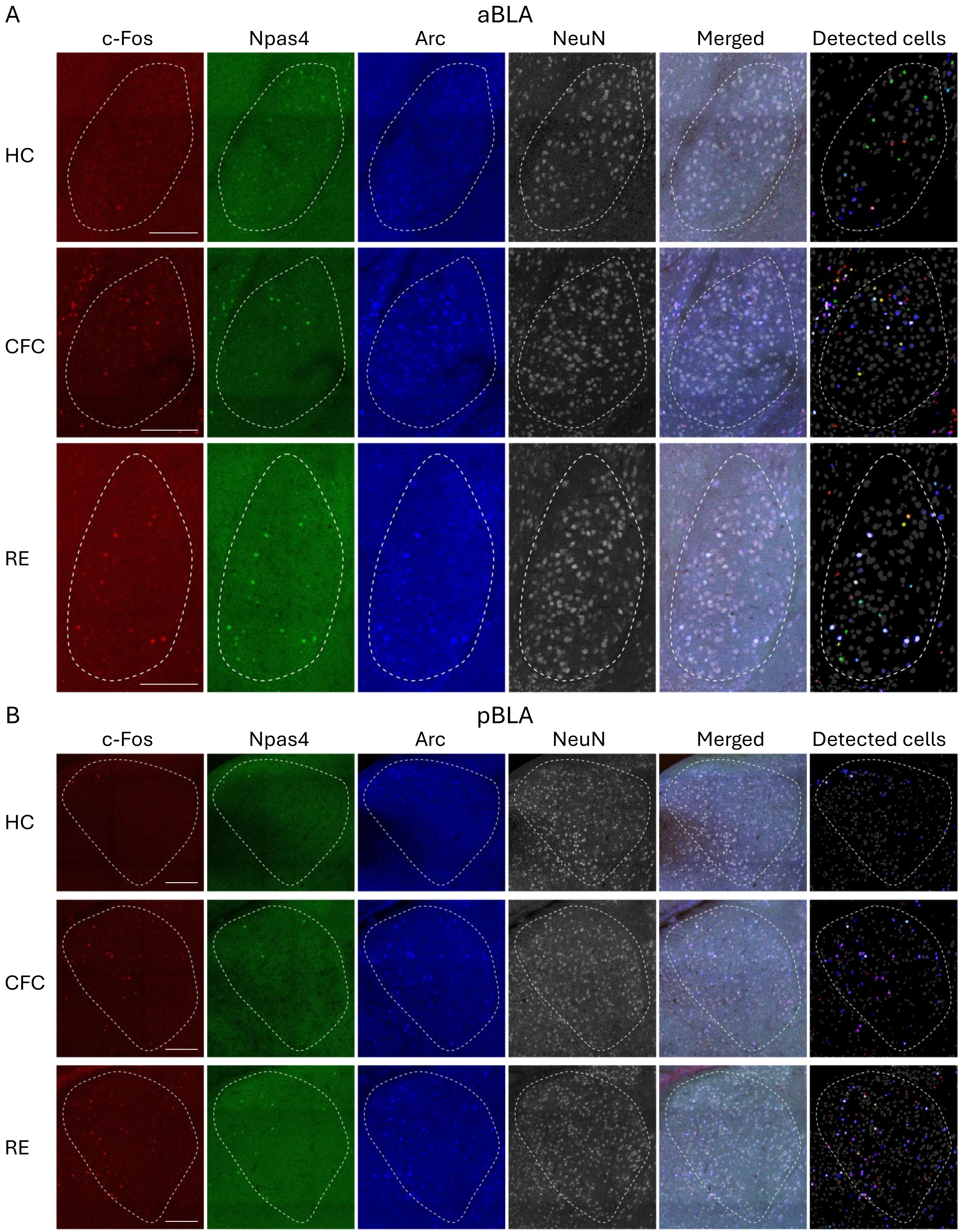

Supp. Figure S3: IEG expression in BLA

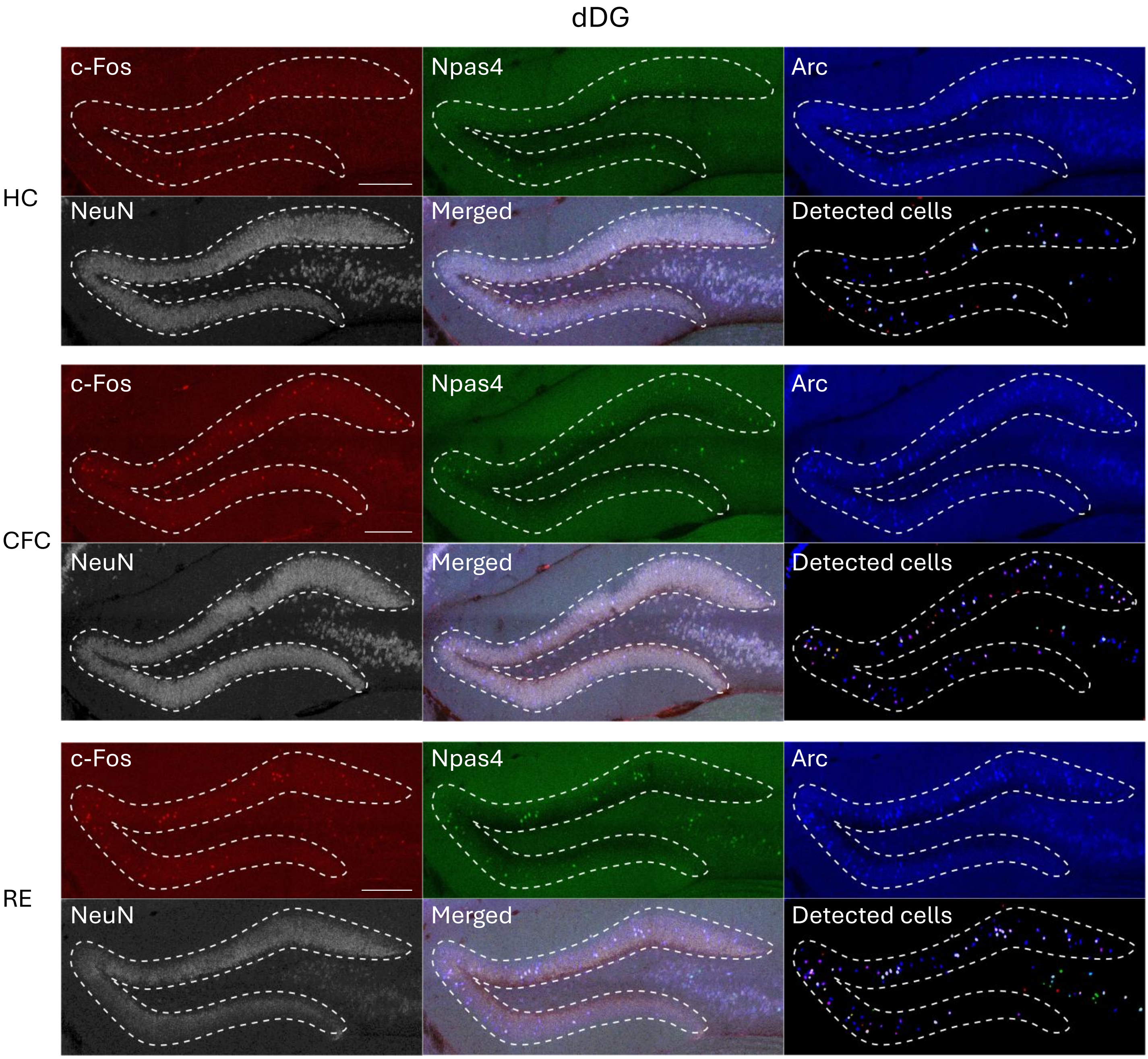

Supp. Figure S4: IEG expression in dDG

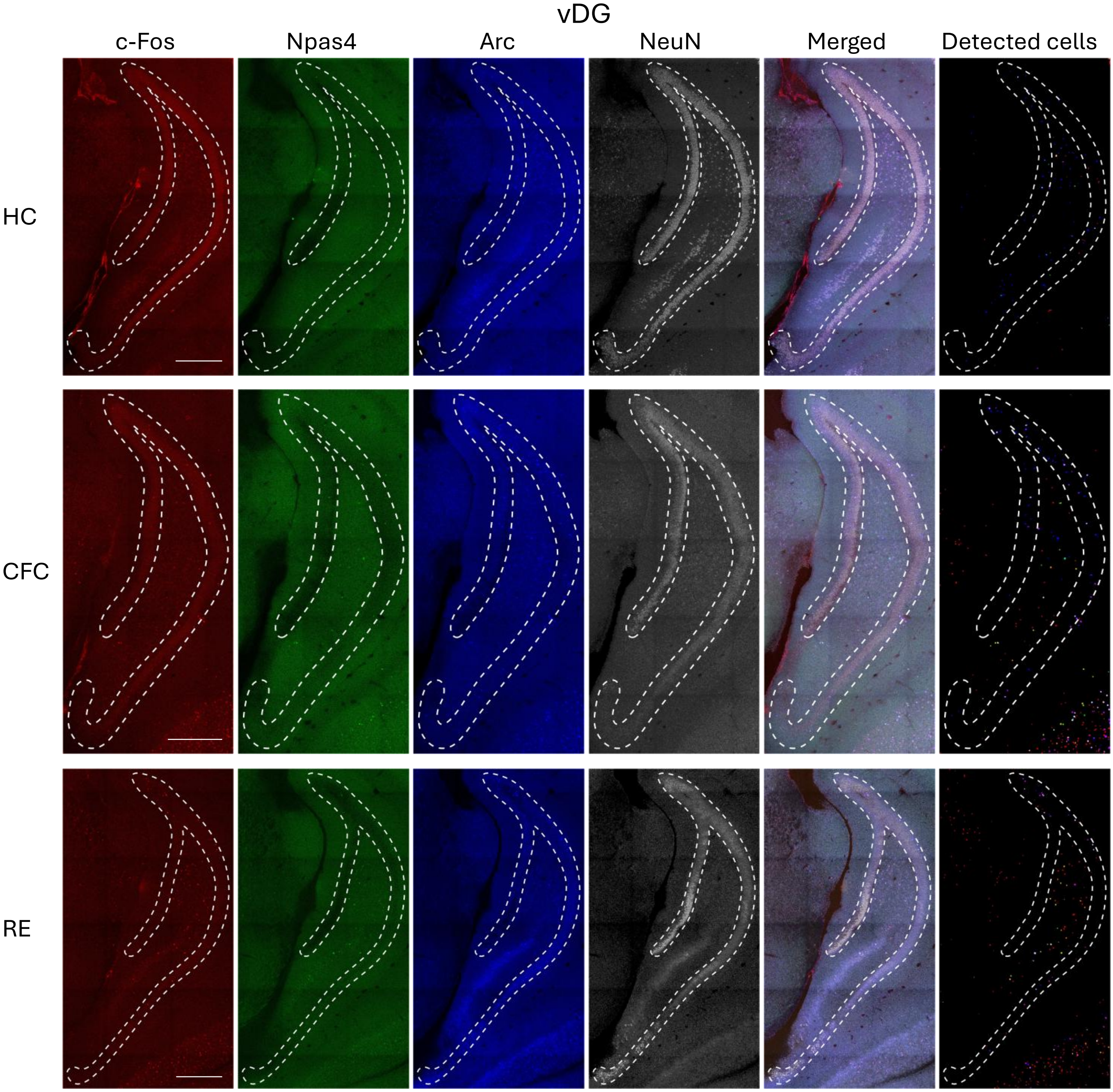

Supp. Figure S5: IEG expression in vDG

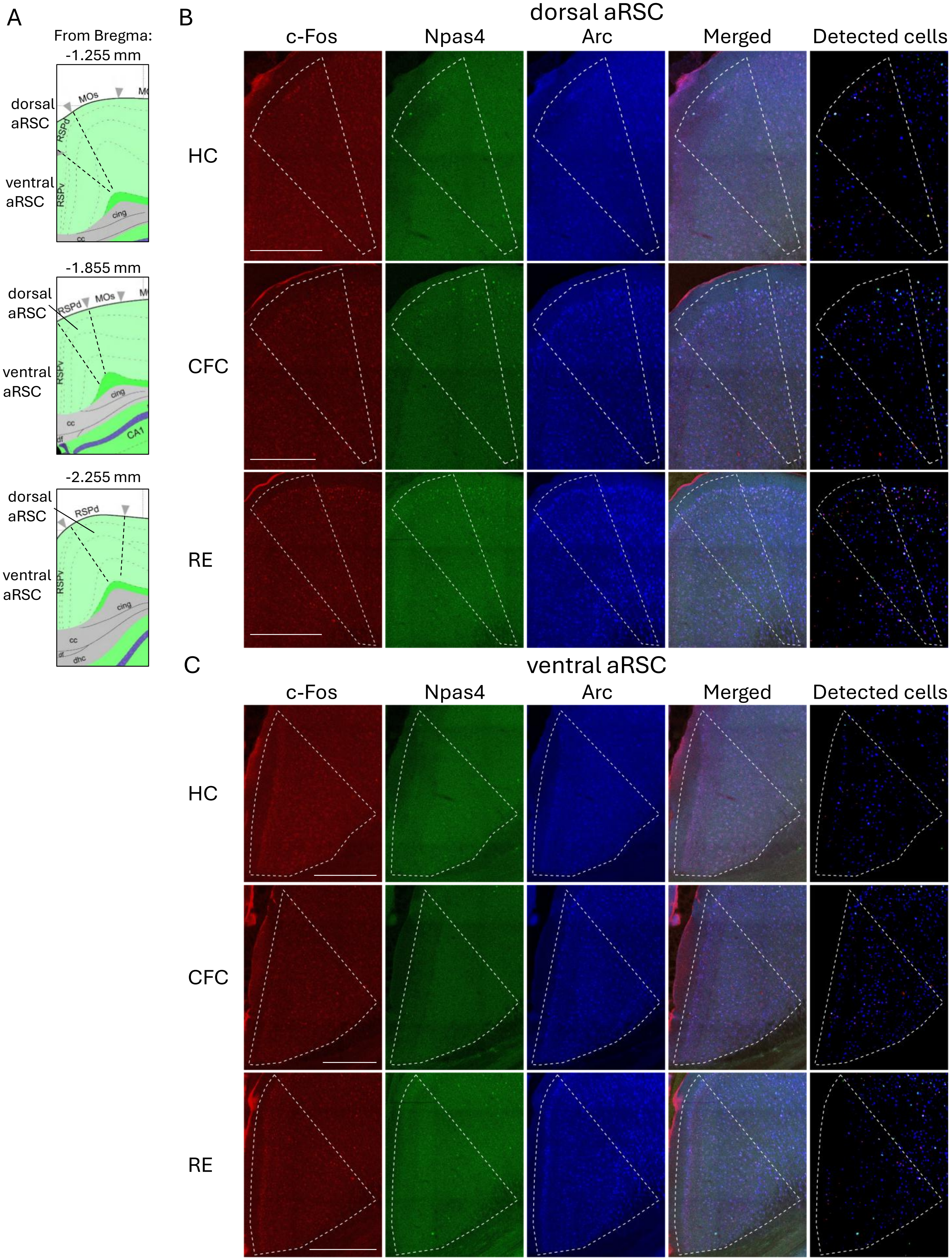

Supp. Figure S6: IEG expression in aRSC

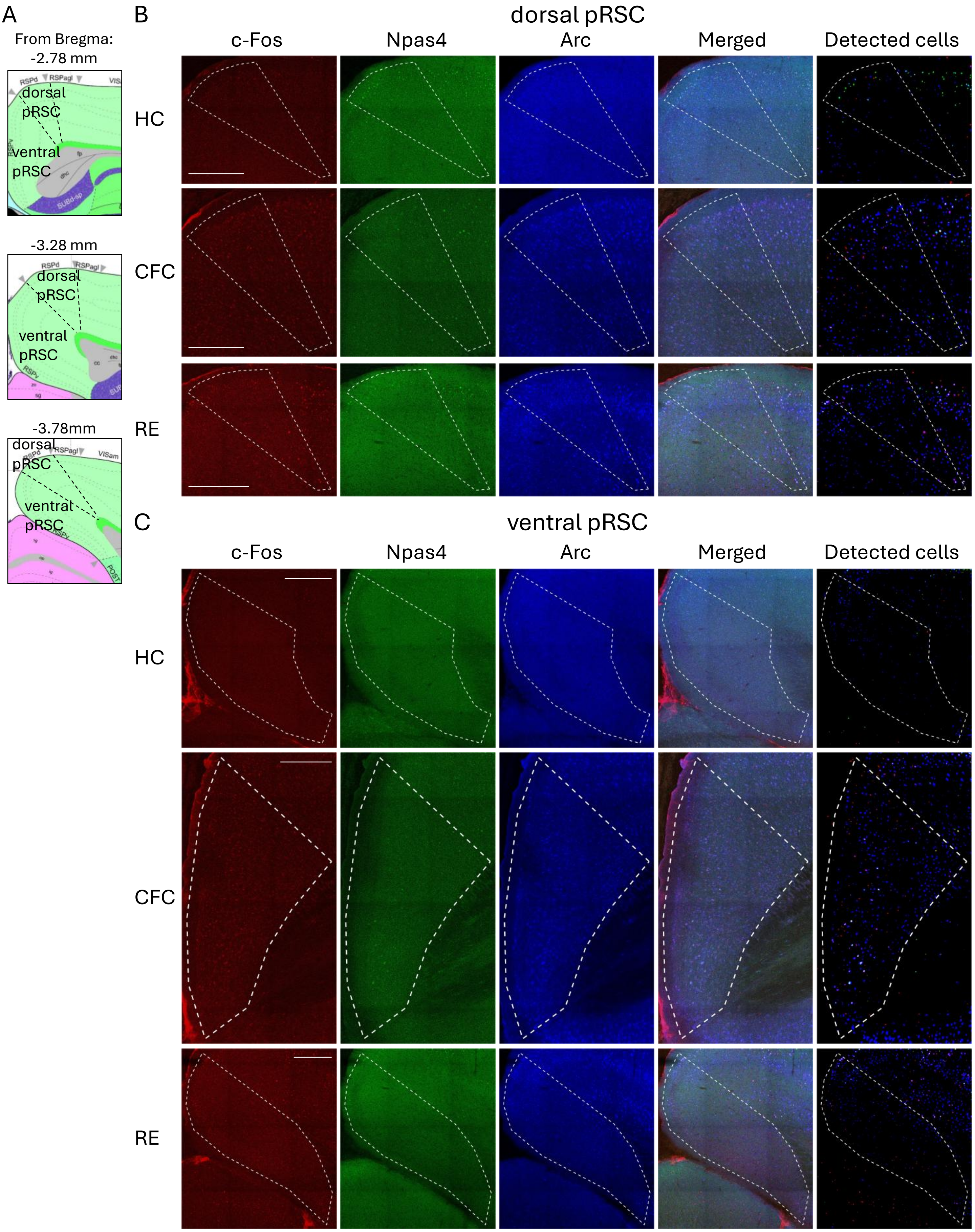

Supp. Figure S7: IEG expression in pRSC

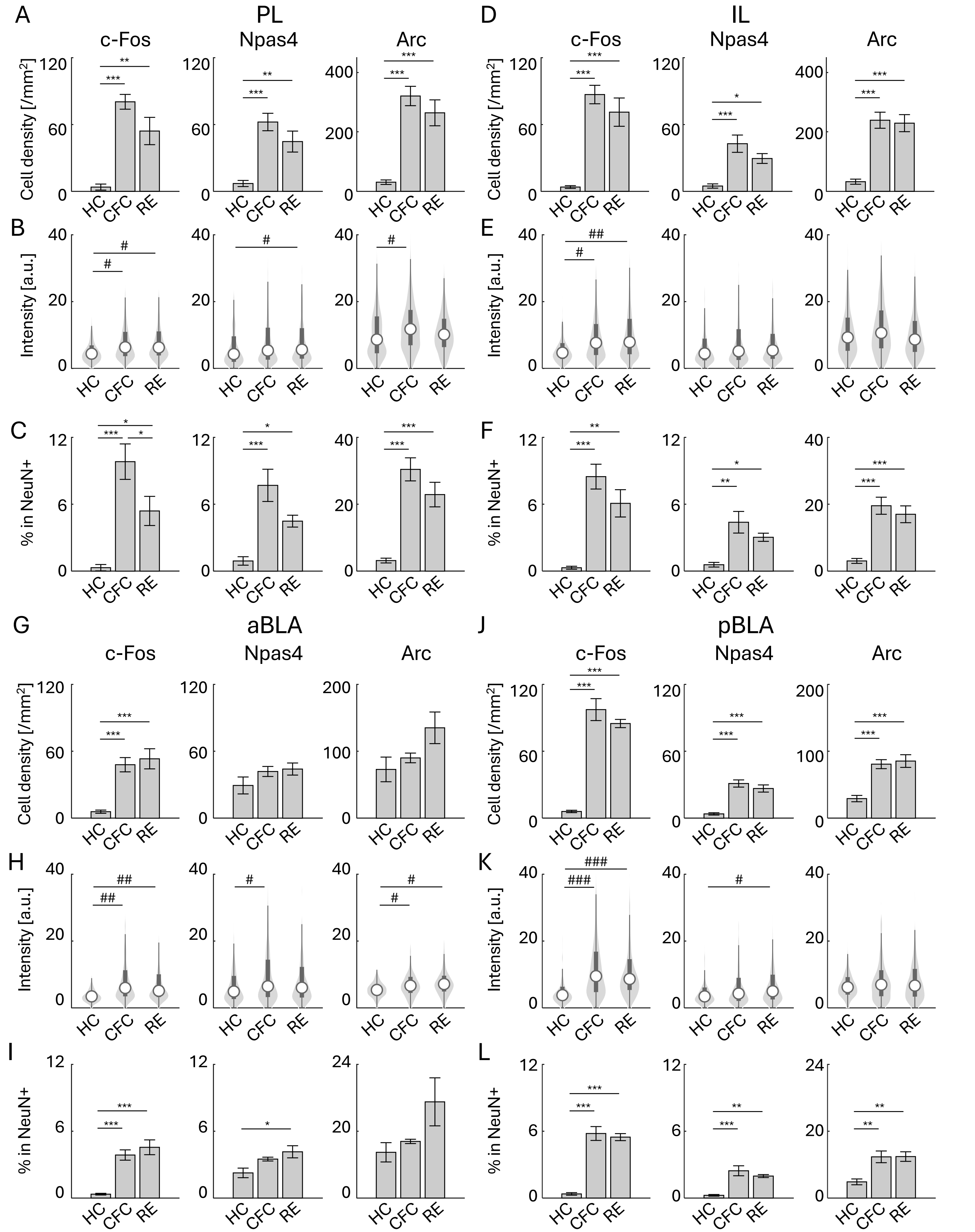

Supp. Figure S8: Cell density and expression level of IEG-positive cells in PFC and BLA

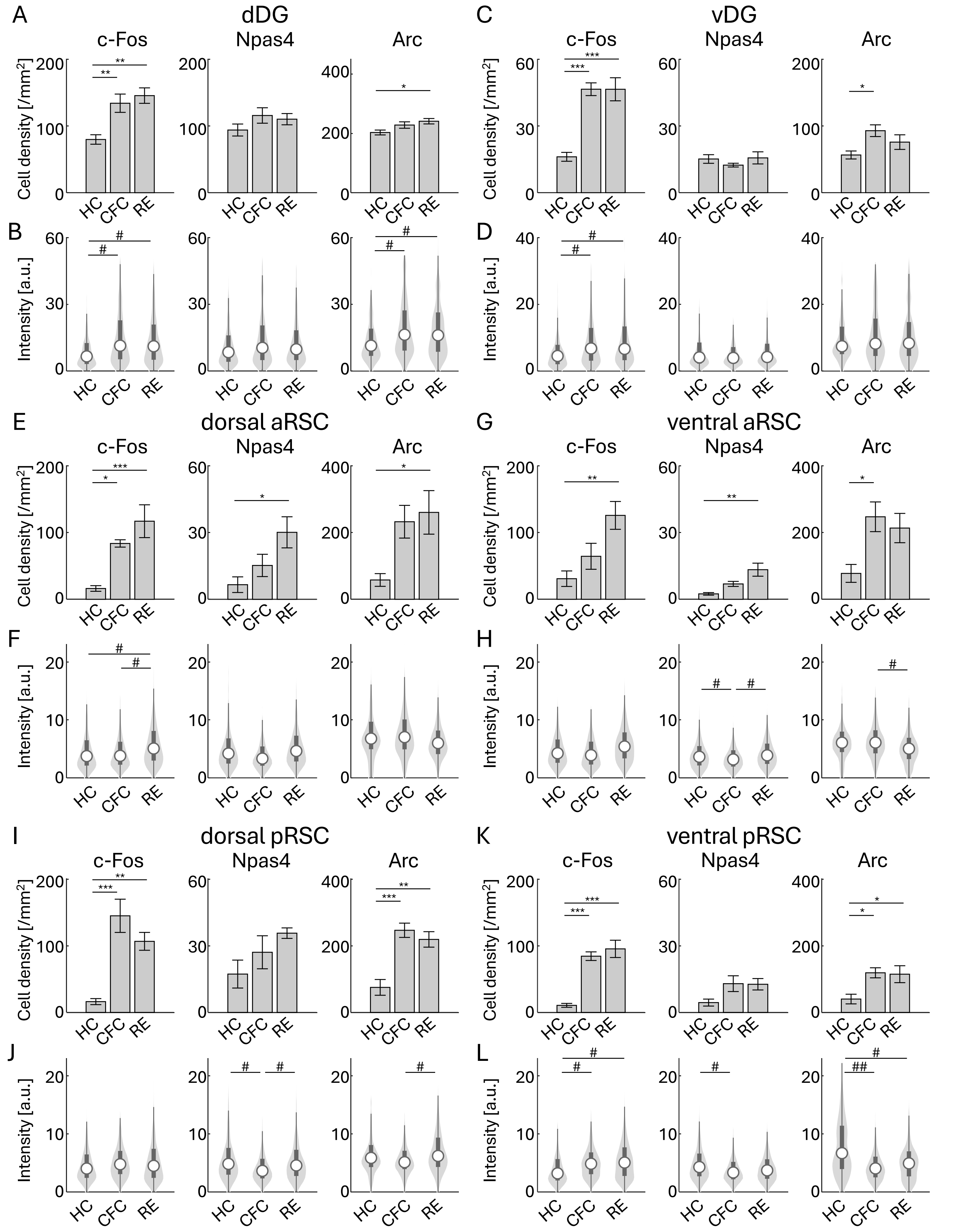

Supp. Figure S9: Cell density and expression of IEG-positive cells in DG and RSC

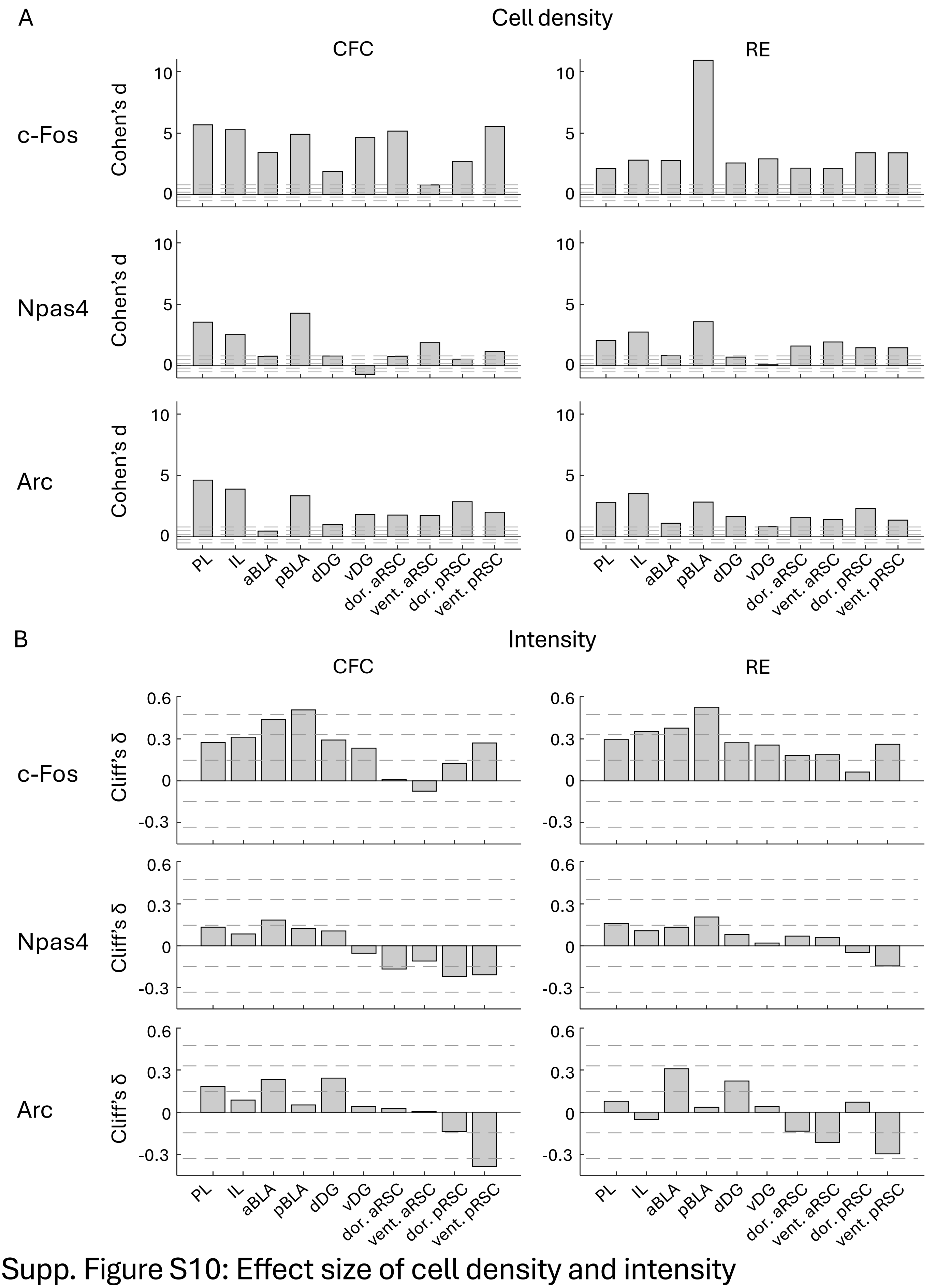

Supp. Figure S10: Effect size of cell density and intensity

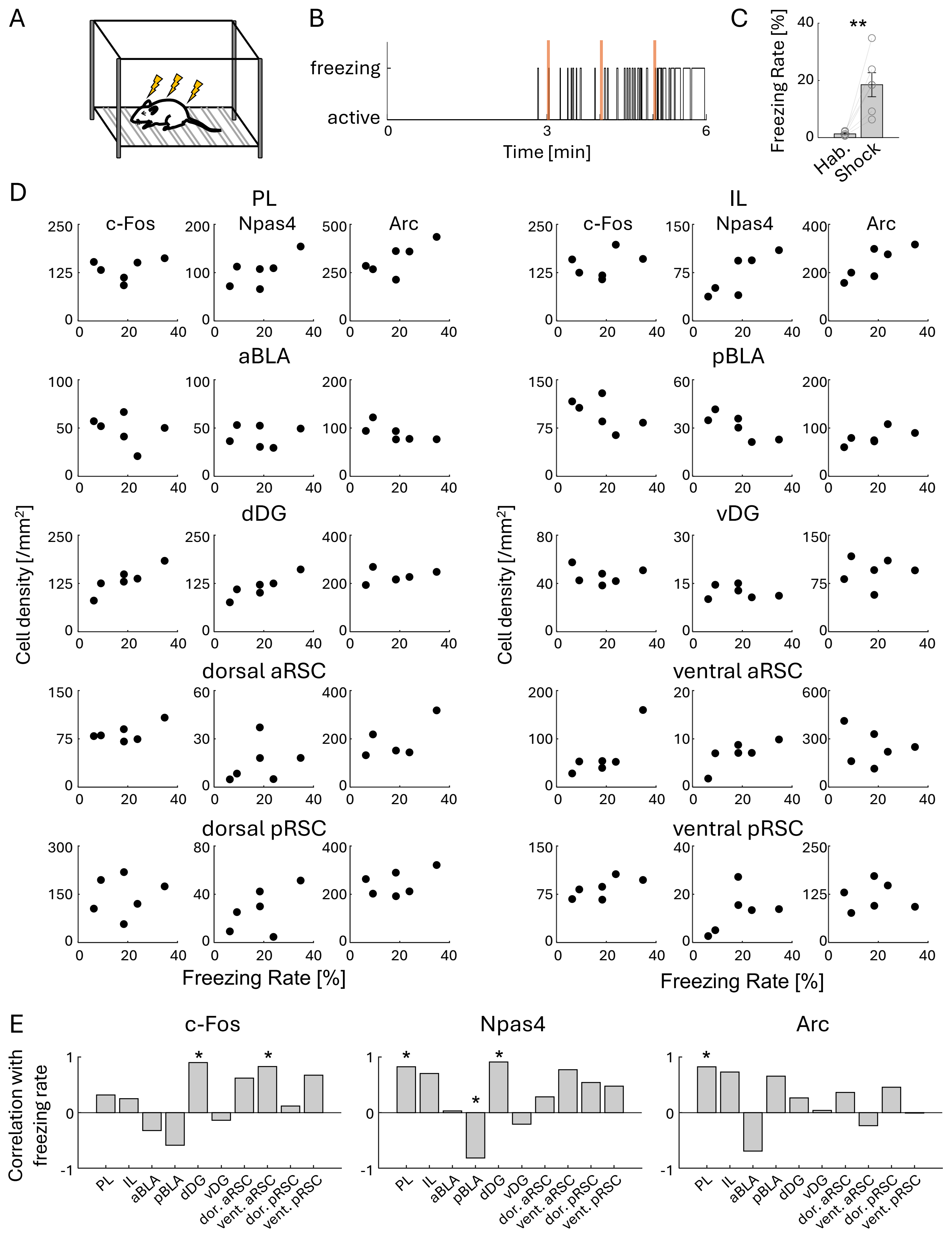

Supp. Figure S11: Correlation between c-Fos-, Npas4-, and Arc-positive cells and freezing behavior in CFC

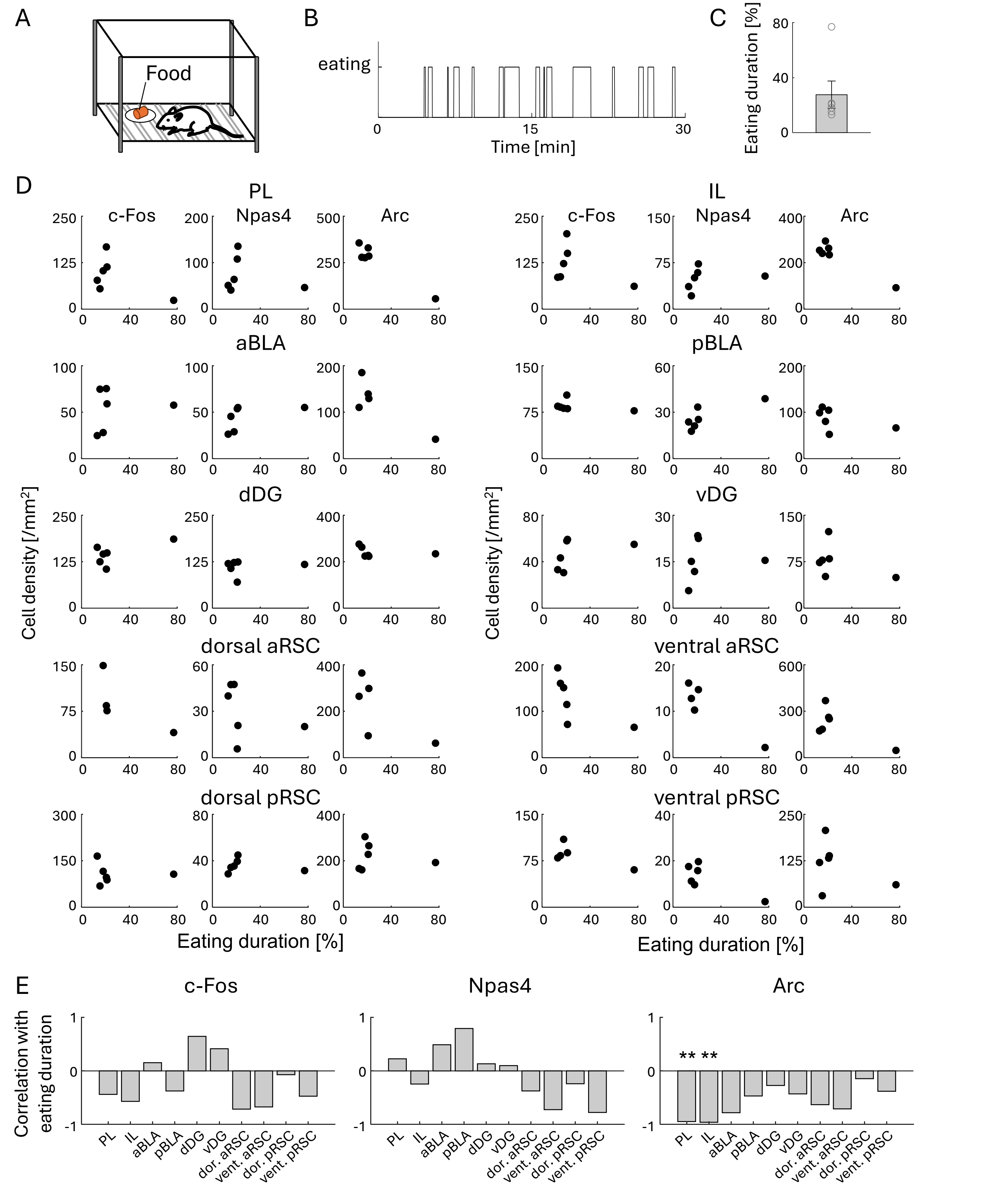

Supp. Figure S12: Correlation between c-Fos-, Npas4-, and Arc-positive cells and eating behavior in RE

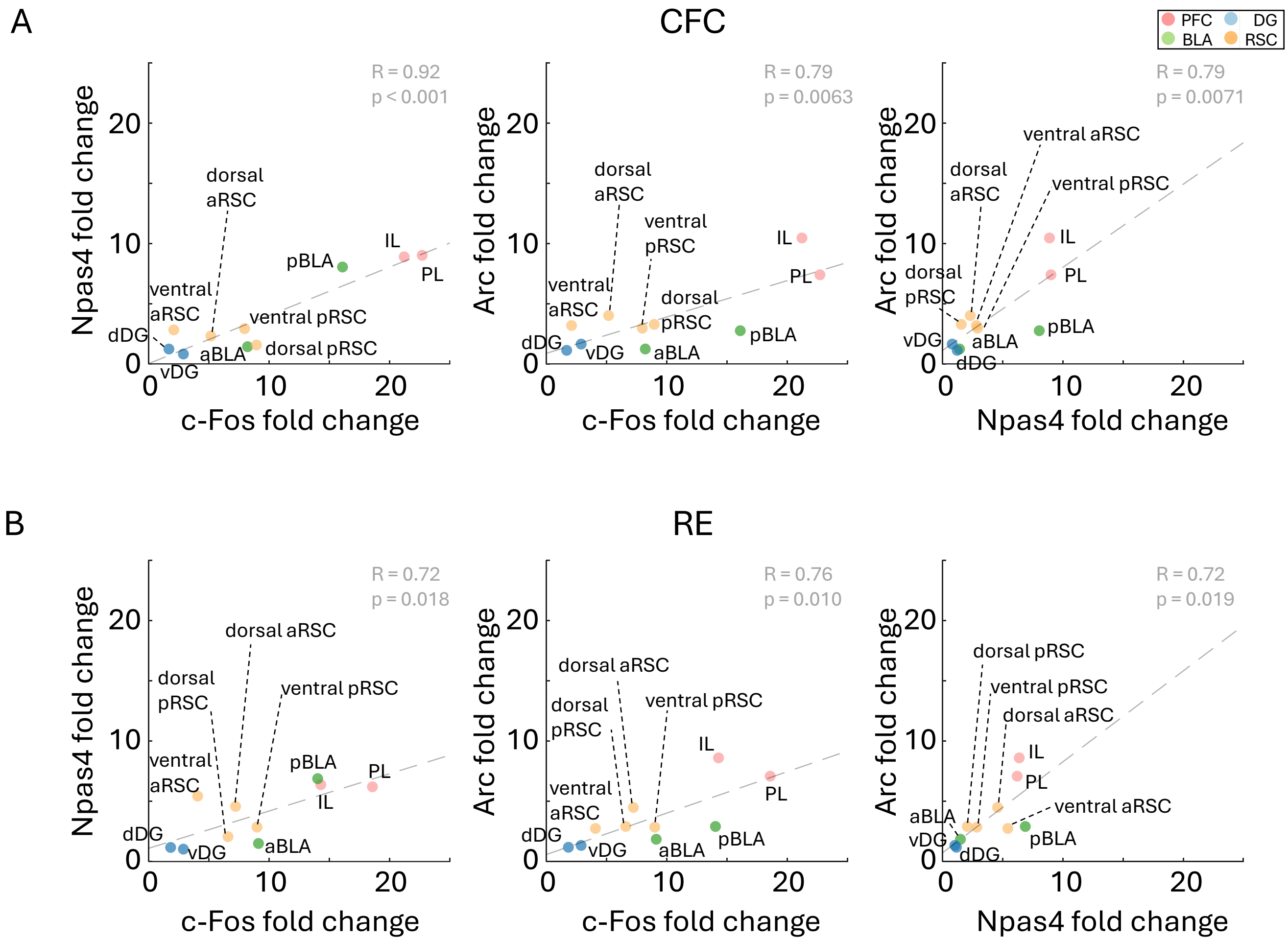

Supp. Figure S13: Cell density changes in each IEG in different brain regions

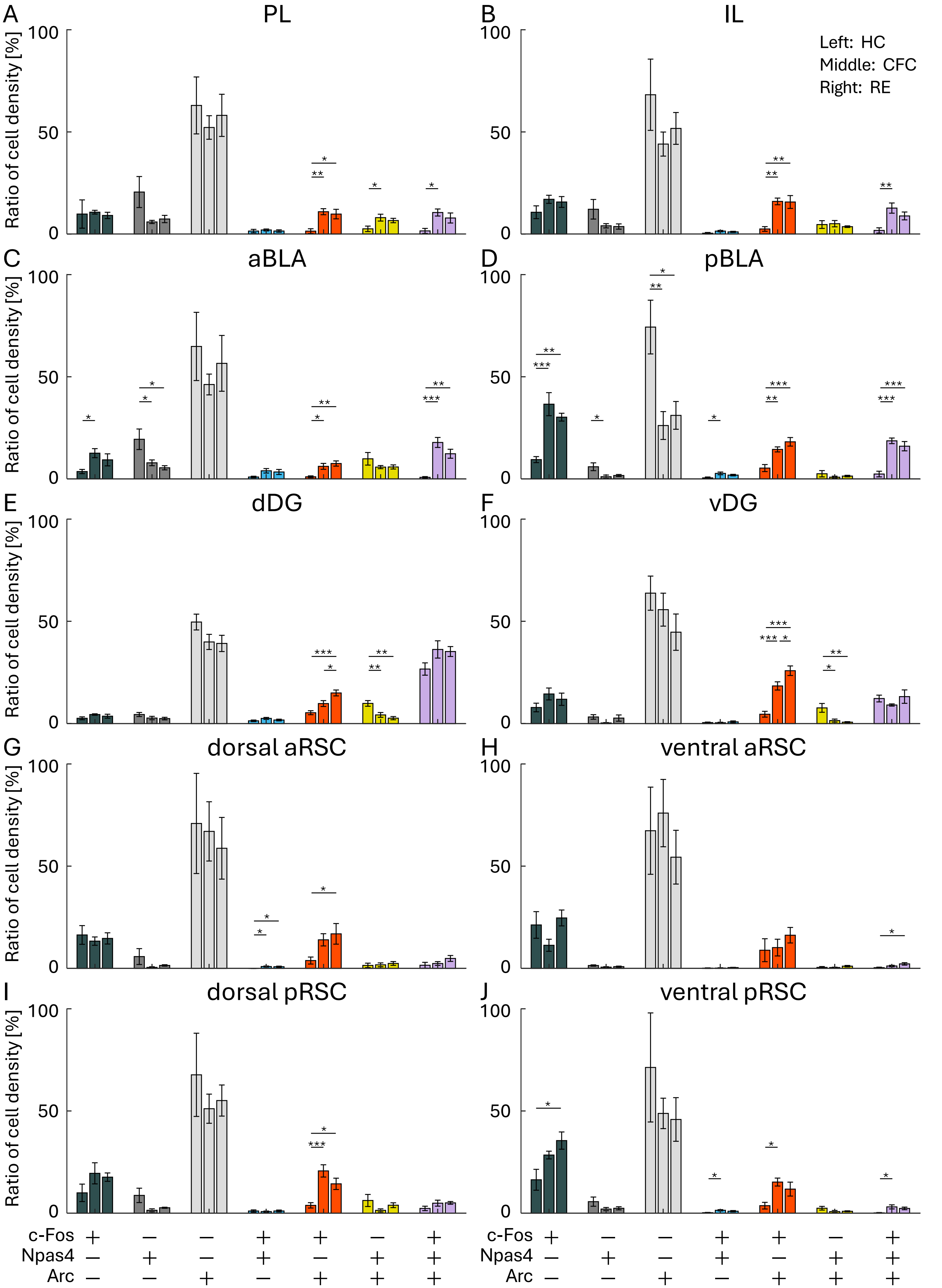

Supp. Fig. S14: Cell density ratio per all IEG-positive cells in each cell group

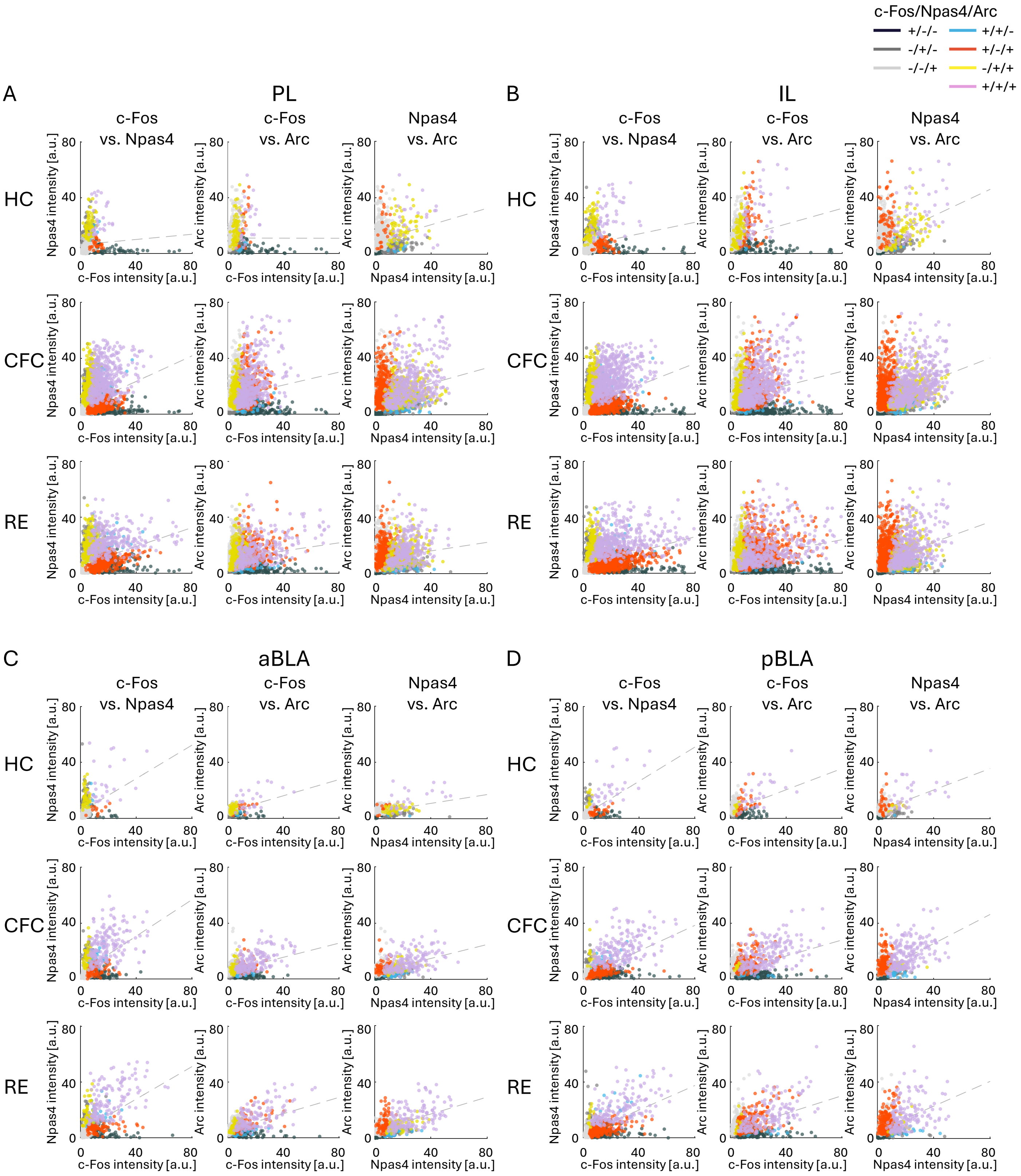

Supp. Figure S15: Intensities of IEGs in individual cells in PFC and BLA

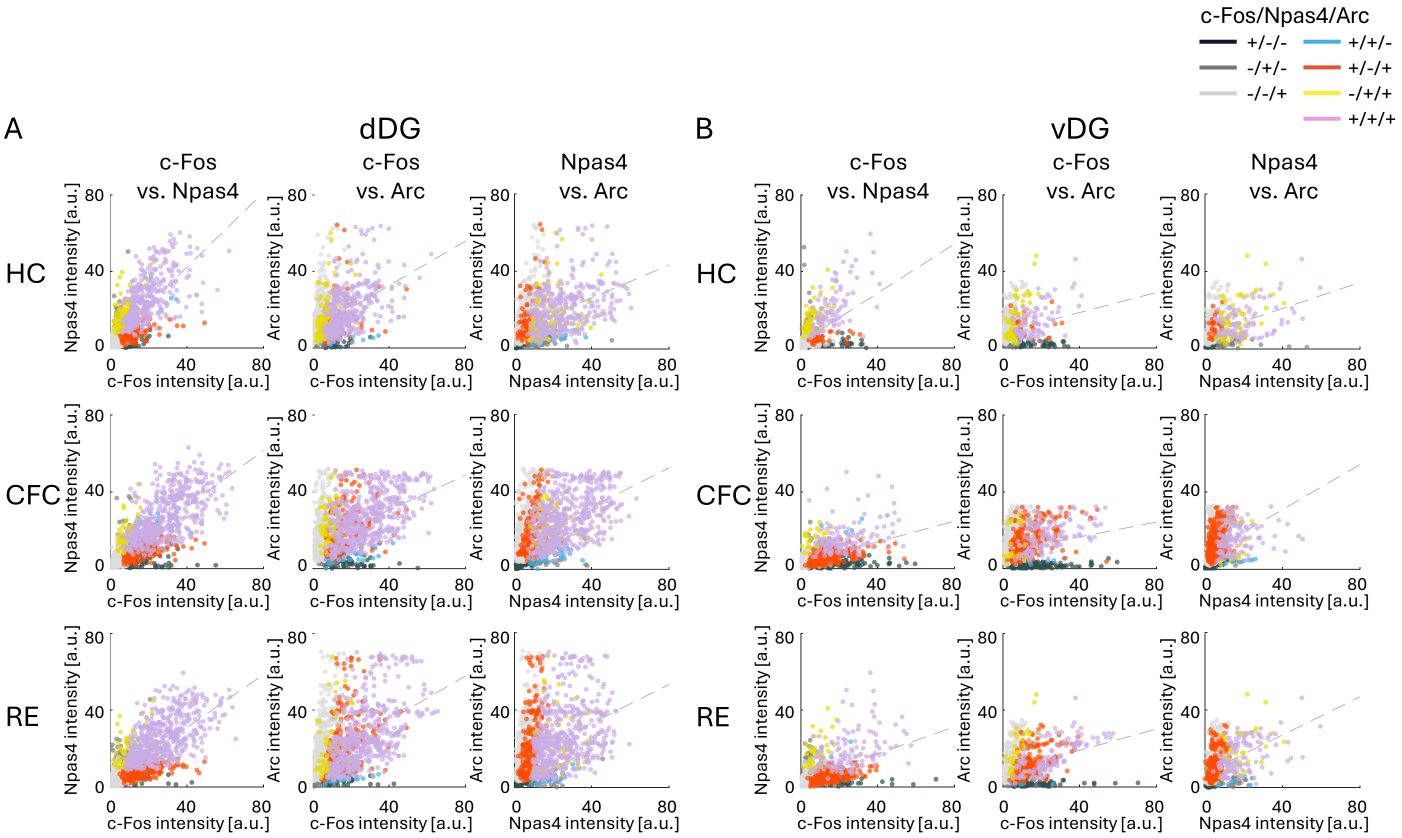

Supp. Figure S16: Intensities of IEGs in individual cells in DG

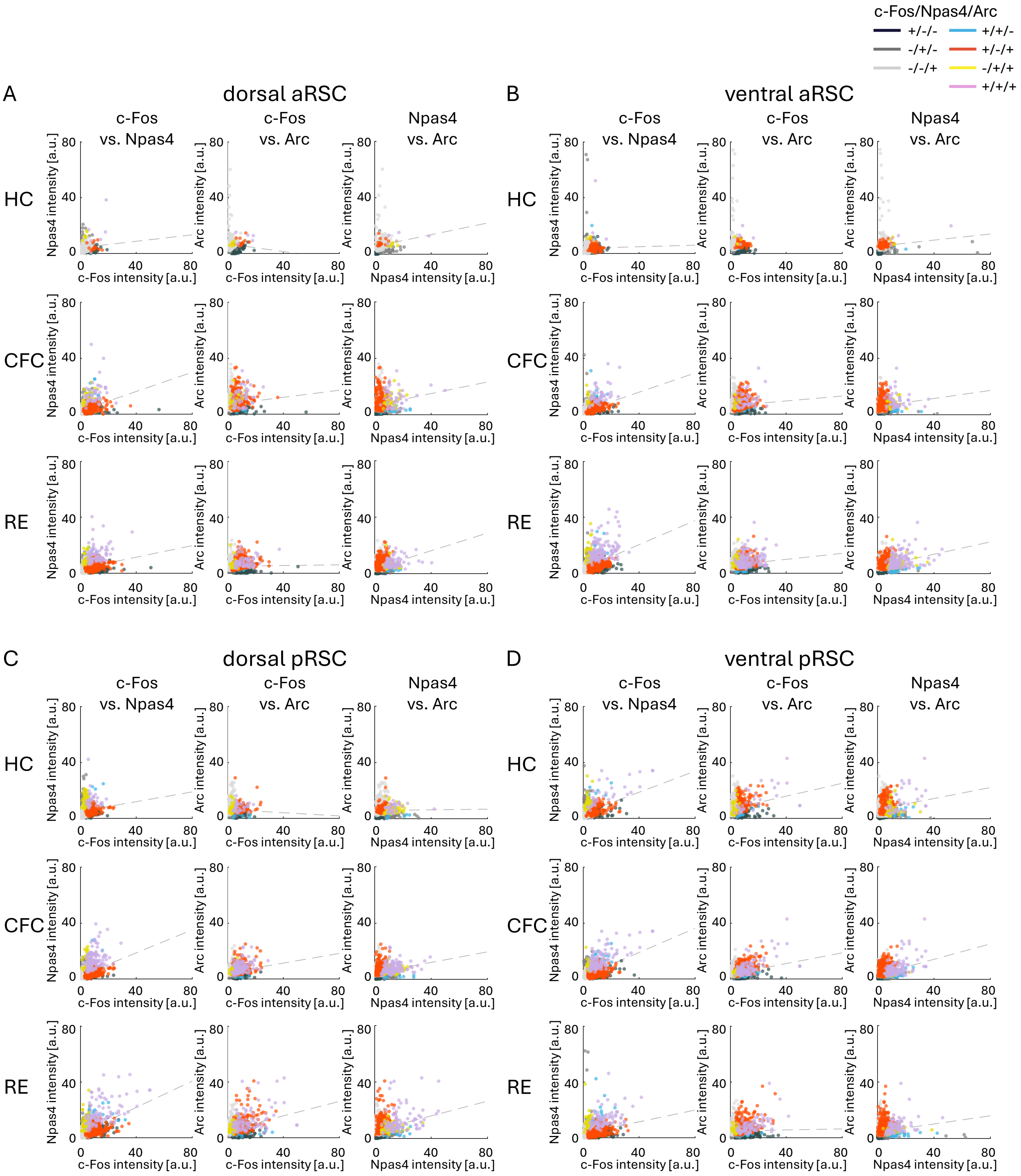

Supp. Figure S17: Intensities of IEGs in individual cells in RSC

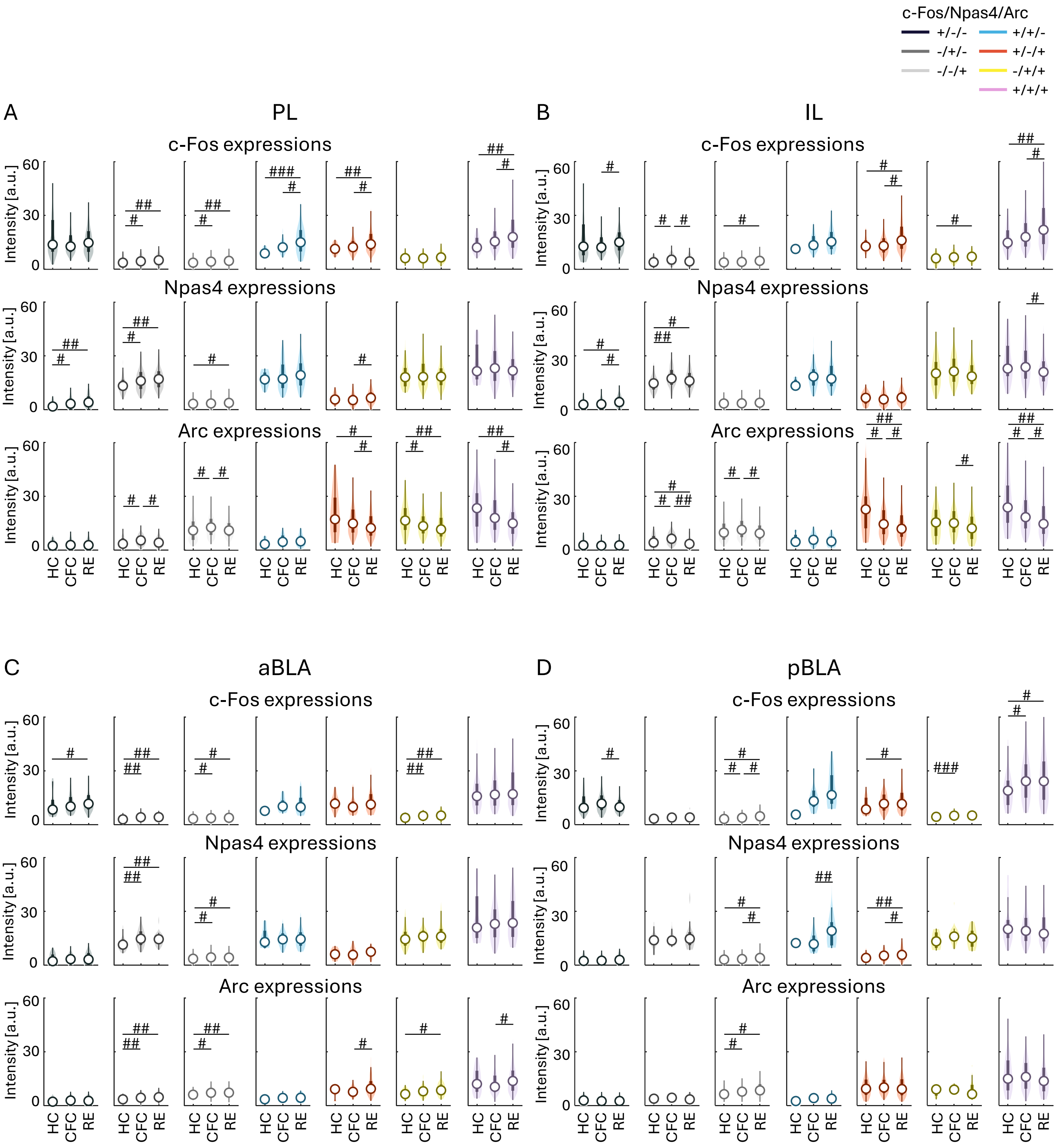

Supp. Figure S18: Intensities of IEGs in each cell group in PFC and BLA

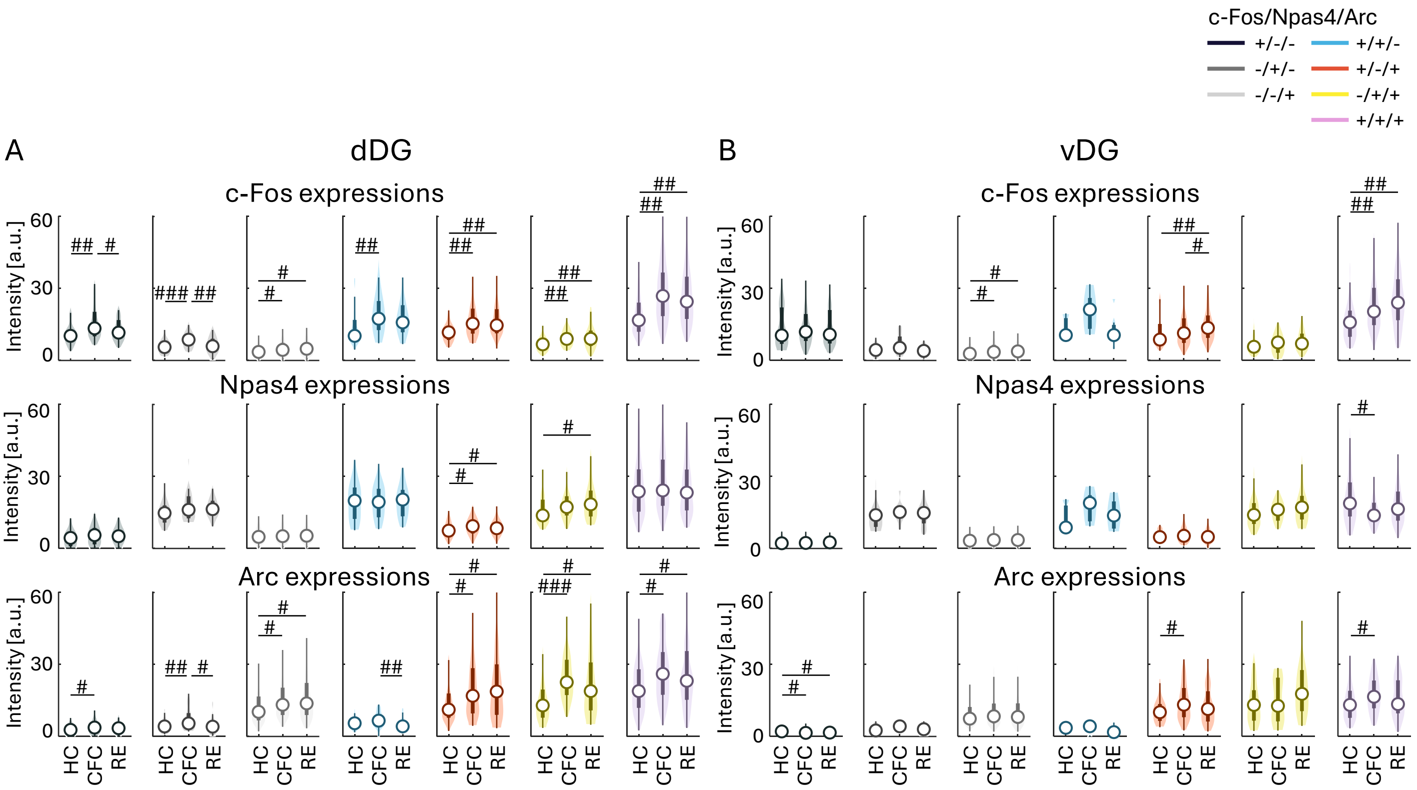

Supp. Figure S19: Intensities of IEGs in each cell group in DG

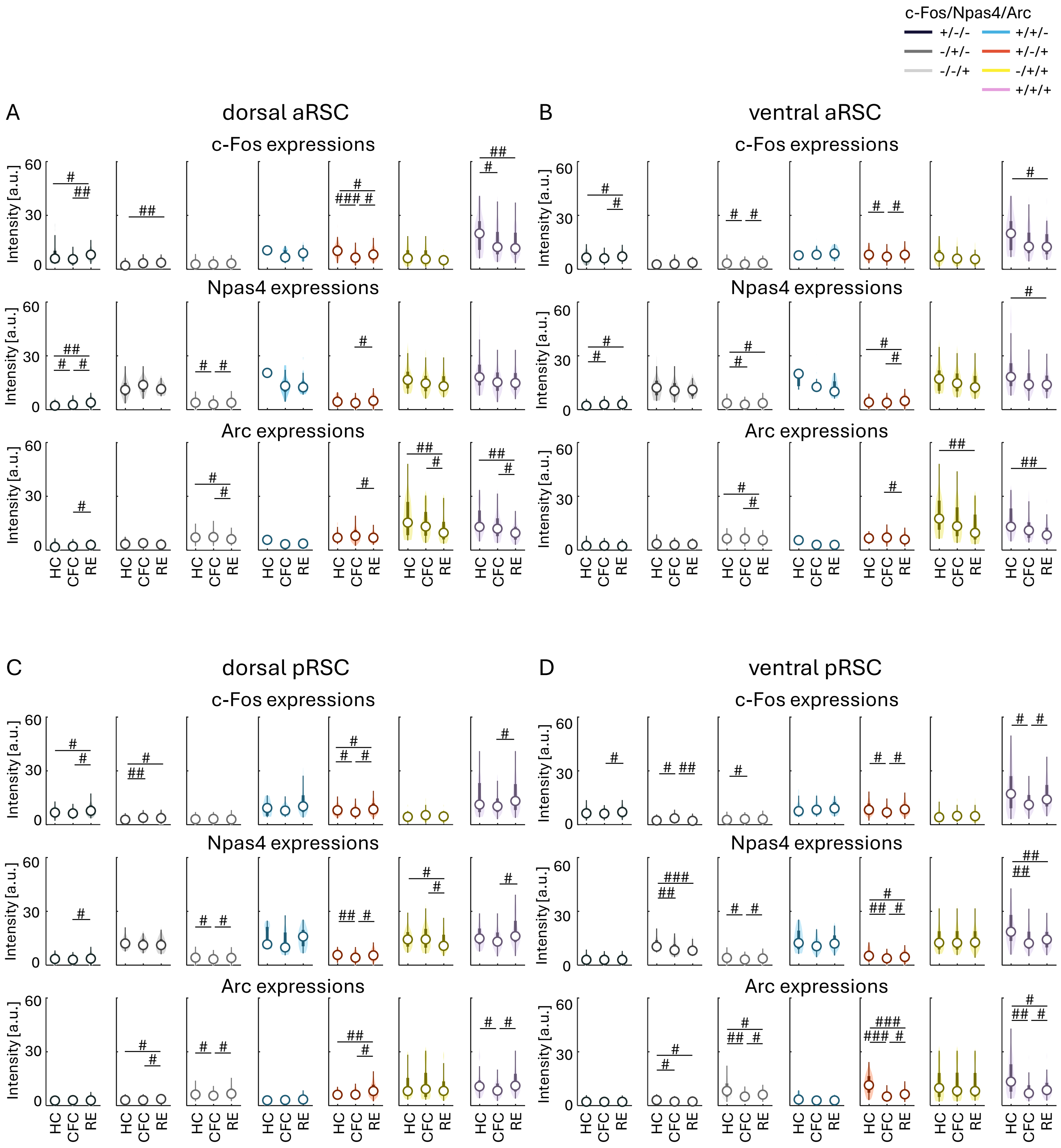

Supp. Figure S20: Intensities of IEGs in each cell group in RSC

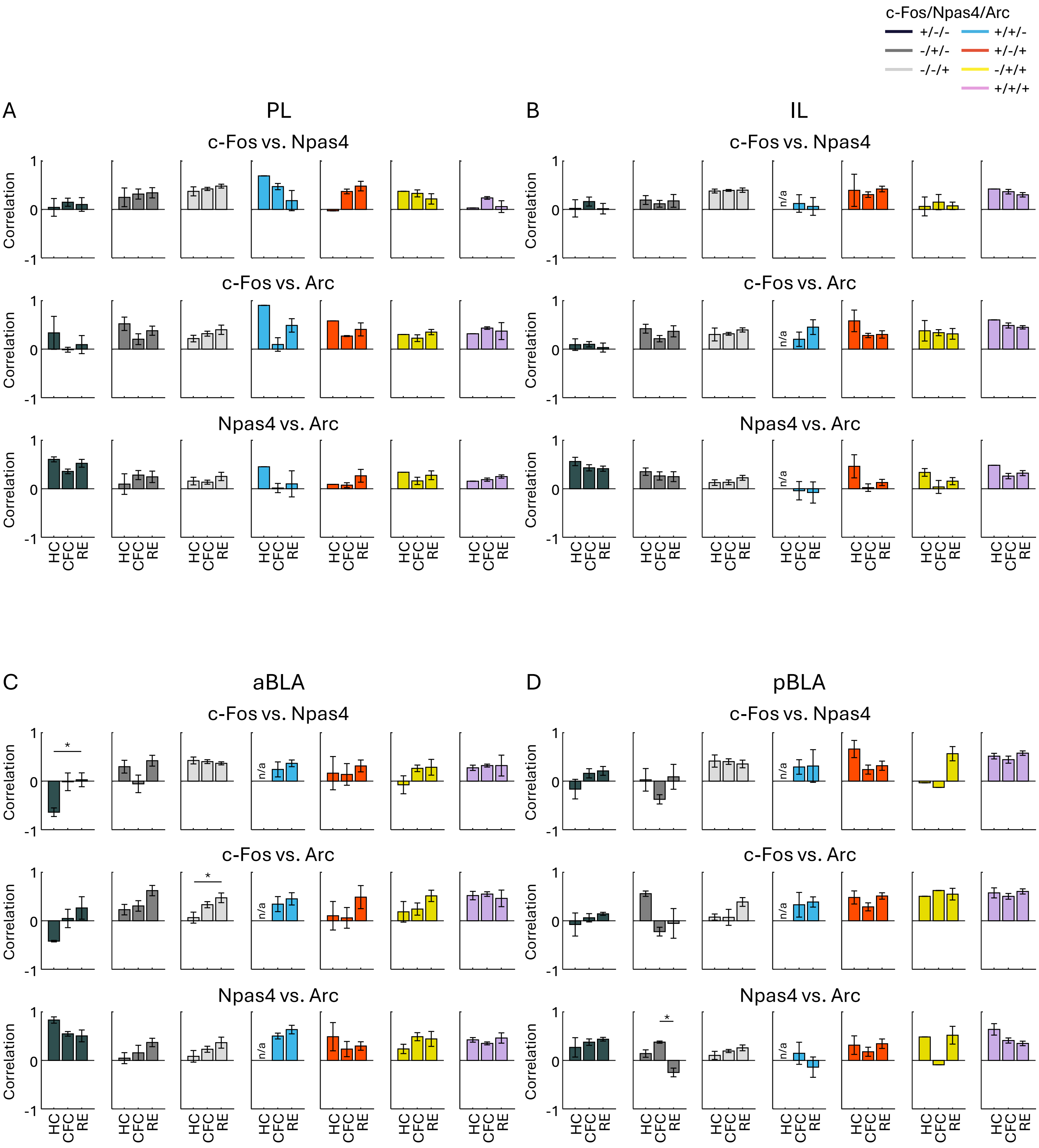

Supp. Figure S21: Intensities correlations in each cell group in PFC and BLA

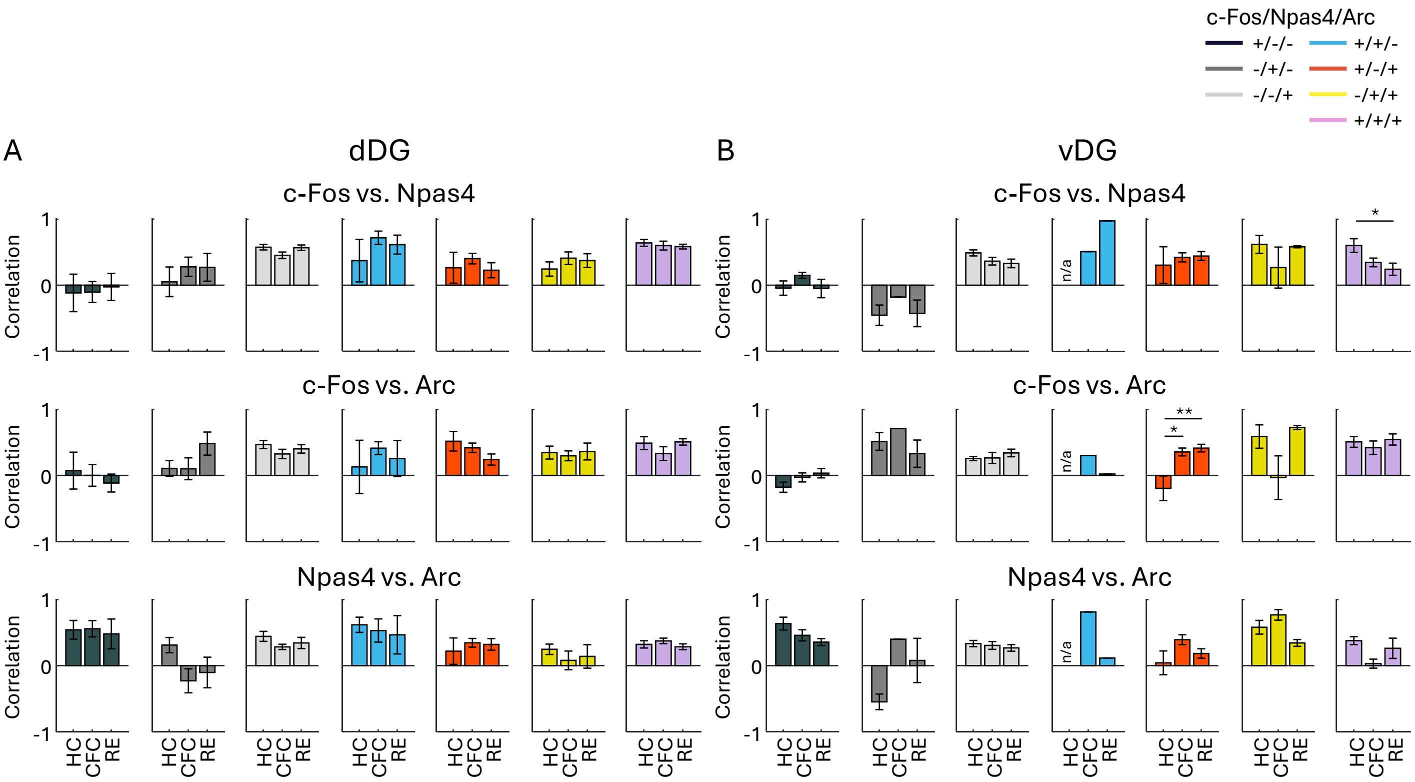

Supp. Figure S22: Intensities correlations in each cell group in DG

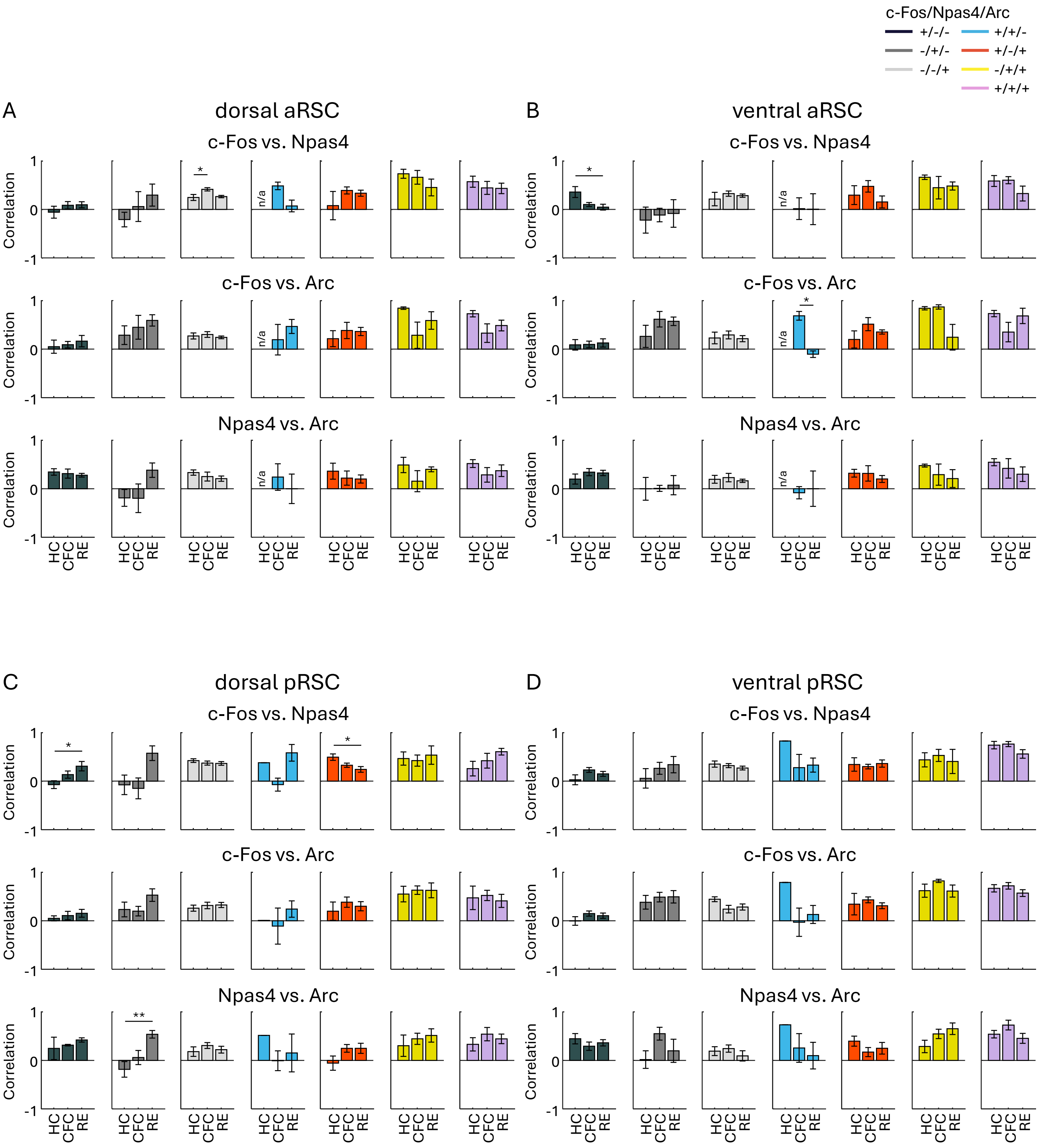

Supp. Figure S23: Intensities correlations in each cell group in RSC

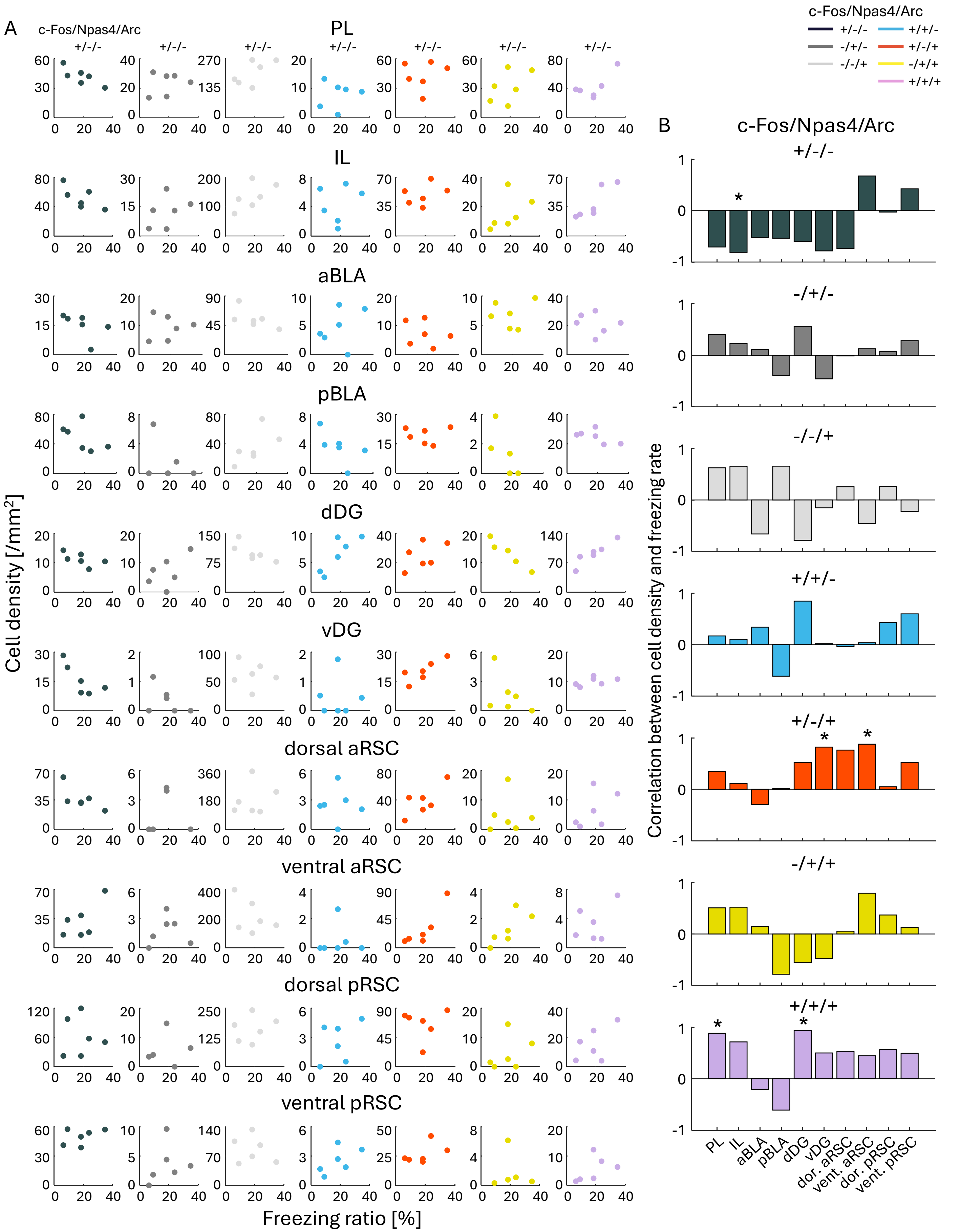

Supp. Figure S24: Correlation between each cell group and freezing behavior

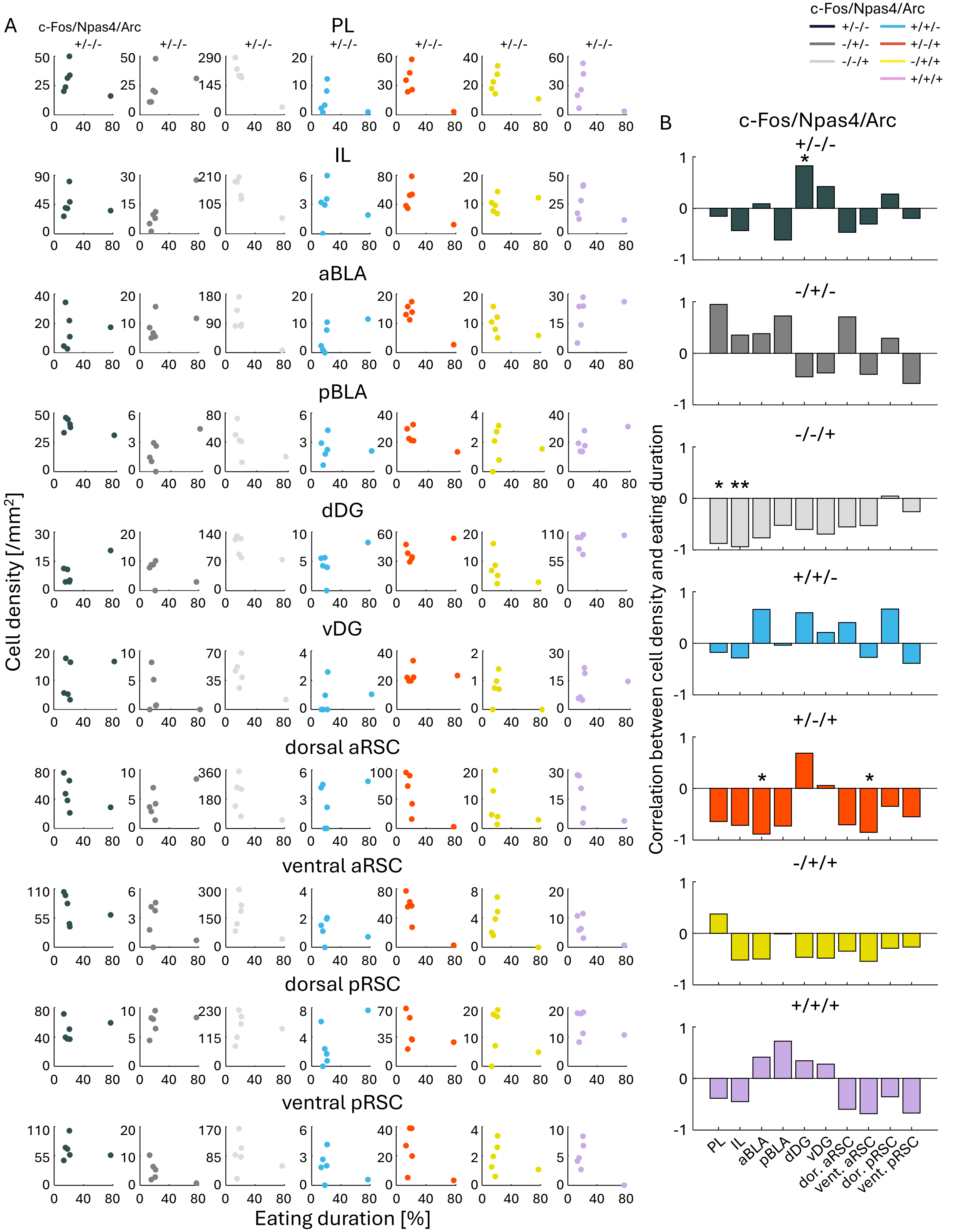

Supp. Figure S25: Correlation of each cell group with eating behavior

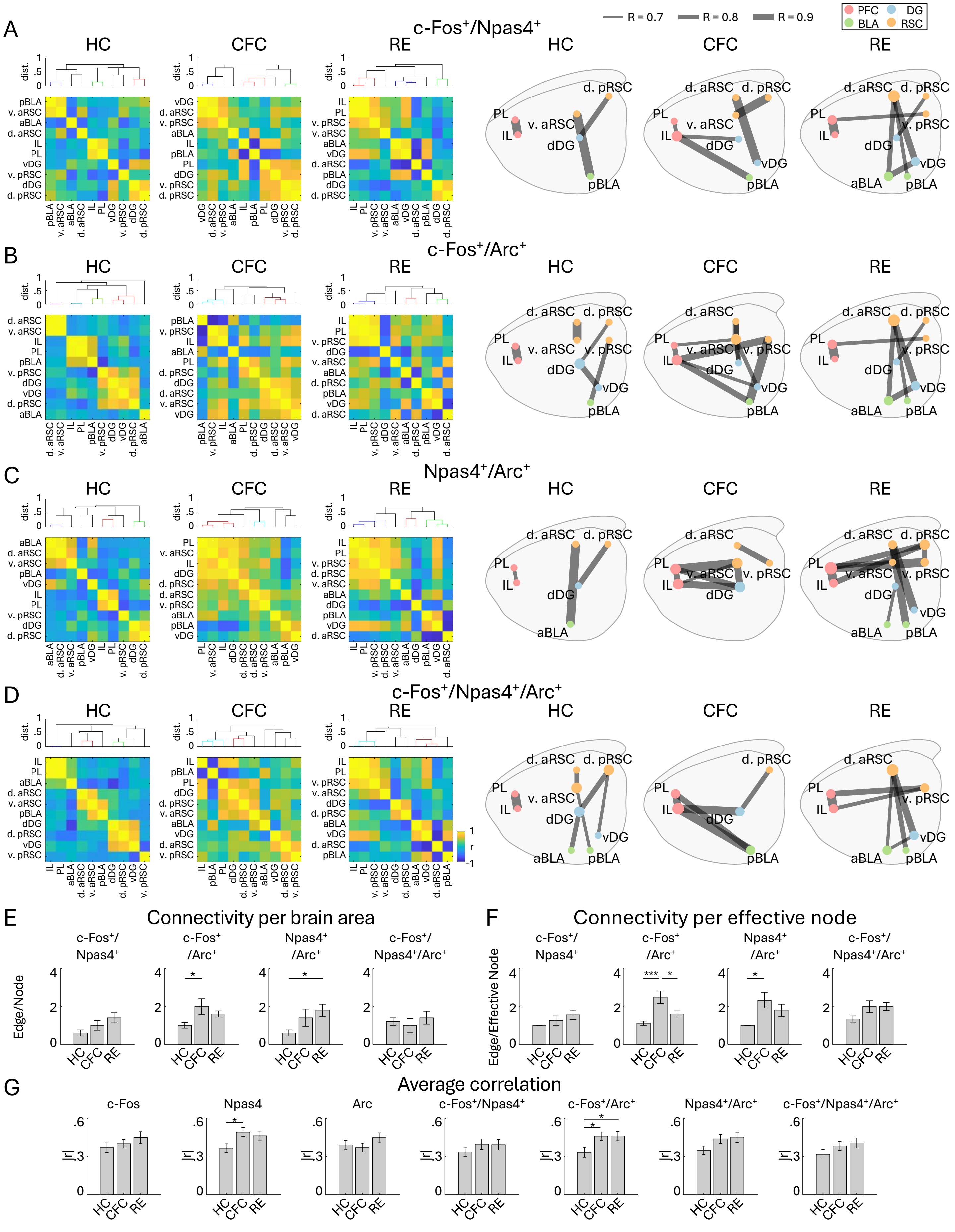

Supp. Figure S26: Functional connectivity network of IEG overlapping cells
